# Manganese‐Based Spinel Cathodes: A Promising Frontier for Solid‐State Lithium‐Ion Batteries

**DOI:** 10.1002/adma.202514126

**Published:** 2025-10-24

**Authors:** Yu Dou, Shiyuan Zhou, Jeremy I. G. Dawkins, Karim Zaghib, Khalil Amine, Gui‐Liang Xu, Sixu Deng

**Affiliations:** ^1^ Department of Chemical and Materials Engineering Concordia University Montreal Quebec H3G 1M8 Canada; ^2^ Chemical Sciences and Engineering Division Argonne National Laboratory Lemont IL 60439 USA; ^3^ Pritzker School of Molecular Engineering The University of Chicago 5801 South Ellis Ave Chicago Illinois 60637 United States

**Keywords:** characterization, manganese, solid‐state batteries, solid‐state electrolytes, spinel cathode

## Abstract

Recently, all‐solid‐state lithium‐ion batteries (ASSLIBs), which exhibit improved safety and enhanced energy density compared to conventional commercialized lithium‐ion batteries (LIBs), thereby have garnered extensive research interest. Among the promising cathode candidates, Mn‐based spinel cathodes LiMn_2_O_4_ (LMO) and LiNi_0.5_Mn_1.5_O_4_ (LNMO), with the unique characteristics of low cost, structural stability, and 3D Li‐ion diffusion channels, have demonstrated excellent performance in LIBs and presented great potential in ASSLIBs applications. However, several challenges, including structural degradations, poor interfacial contact, large interfacial resistance, and Mn‐dissolution/diffusion during the electrochemical cycling, hinder their practical applications and commercialization in the ASSLIBs. Particularly, the high‐voltage LNMO cathodes suffer from the challenge of electrochemical incompatibility with most of the solid‐state electrolytes (SSEs). Herein, the spinel structure, the electrochemical behavior, and the structural degradation of the LMO/LNMO are explored. The characteristics and recent progress of the mitigating strategies to the challenges of various SSEs, including polymer‐, oxide‐, composite‐, sulfide‐, halide‐, and LiPON‐based SSEs, are introduced when paired with LMO/LNMO. Finally, the directions for future research to advance Mn‐based spinel cathodes and fulfill the requirements of the next‐generation ASSLIBs are also discussed.

## Graphical abstract

Mn‐based spinel cathodes LiMn_2_O_4_ (LMO) and LiNi_0.5_Mn_1.5_O_4_ (LNMO), with the unique characteristics of low cost, structural stability, and 3D Li‐ion diffusion channels, have presented great potential in all‐solid‐state batteries. Here, a comprehensive understanding and valuable insights into the rational design and implications for the future development of Mn‐based cathodes are provided.

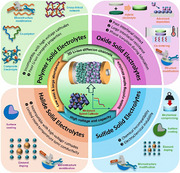

## 1 Introduction

**Figure 1:**
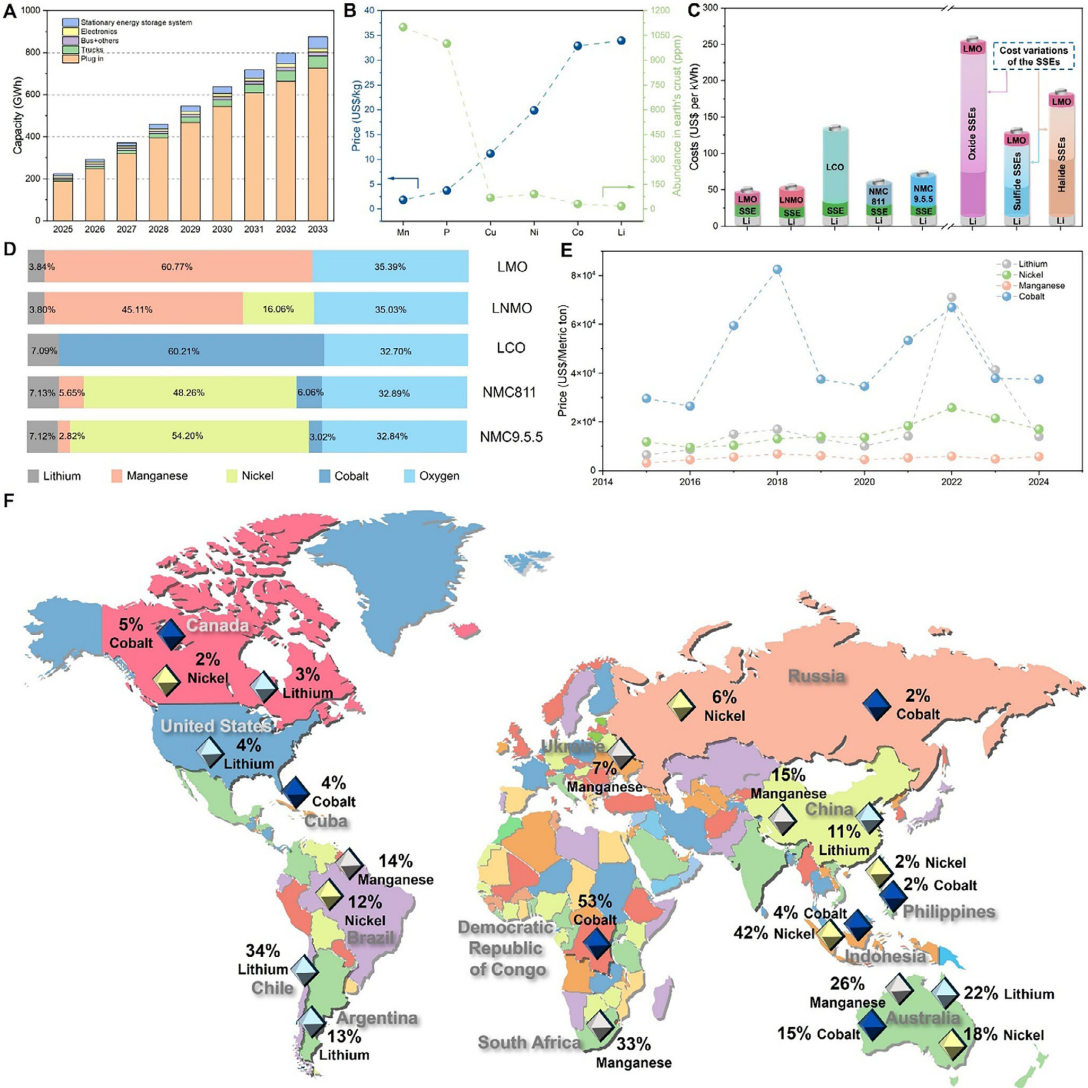
A) Prediction of solid‐state battery addressable market size between 2025 and 2033. Data derived from ref. [7]. B) Prices of selected elements and the abundances in the Earth's crust. Data derived from refs. [28, 42]. C) Material costs estimation of different ASSLIBs chemistries. Calculation on the left based on the SSE at the cost threshold of 50 US$ kg^−1^. Calculation based on the LMO cathode with price ranges of different SSEs is on the right. Data derived from refs. [17, 18, 39, 44]. D) Weight ratio of the elements in typical cathodes. E) Average price of different metals from 2015 to 2024. Data derived from ref. [45]. F) The worldwide distribution of Li/Co/Mn/Ni mineral reserves. Reproduced with permission.^[^ ^46^ ^]^ Copyright 2024, American Chemical Society. Data derived from refs. [45, 47].

Lithium‐ion batteries (LIBs) have become increasingly vital for efficiently storing and delivering electrical energy. With the surging demand for electric vehicles (EVs) to address global energy and environmental challenges, extensive research interests have shifted toward all‐solid‐state lithium‐ion batteries (ASSLIBs).^[^ ^1^, ^2^, ^3^, ^4^, ^5^, ^6^ ^]^ As shown in **Figure** **1**A, the prediction of the addressable market for solid‐state batteries from 2025 to 2033 further illustrates the high demand and necessity of developing ASSLIBs as the next‐generation energy storage technology. In 2033, it is expected to exhibit a market value of US$8 billion for solid‐state batteries.^[^ ^7^ ^]^

Compared to conventional LIBs that use highly flammable, volatile, and toxic organic liquid electrolytes, by employing solid‐state electrolytes (SSEs), the ASSLIBs offer enhanced safety, leak‐free operation, and elevated energy density. These remarkable advancements position the ASSLIBs as promising candidates for next‐generation batteries. Furthermore, the capability to directly utilize Li metal, which possesses a high theoretical specific capacity of 3860 mAh g^−1^ and the lowest electrochemical potential of −3.04 V vs SHE (standard hydrogen electrode) as an anode, further increases the energy density of ASSLIBs.^[^ ^8^, ^9^, ^10^, ^11^, ^12^, ^13^, ^14^ ^]^ Despite these promising features, the high cost of some SSEs remains a significant obstacle. For instance, one of the most widely used sulfide‐based SSEs, Li_6_PS_5_Cl (LPSCl), requires at least 30% of high‐cost Li_2_S of 654.18 US$ kg^−1^,^[^ ^15^ ^]^ thus resulting in the cost of LPSCl over 195 US$ kg^−1^, which significantly exceeded the threshold of material cost for SSEs of 50 US$ kg^−1^ for commercialization.^[^ ^16^ ^]^ Concurrently, when preparing ASSLIBs with oxide‐based SSEs, the requirement of over 1000 °C high‐temperature sintering to obtain sufficient densification and ionic conductivity crucially elevates the fabrication cost.^[^ ^17^ ^]^ Halide‐based SSEs are also remarkably expensive, because the majority of non‐Li‐containing rare earth/indium (In) chloride precursors are extremely expensive (>1000 US$ kg^−1^).^[^ ^15^, ^18^ ^]^ The high cost of the SSEs has become a drastic hurdle in making ASSLIBs‐based EVs financially affordable. Additionally, persistent challenges with ASSLIBs include limited ionic conductivity of SSEs at room temperature, interface instability with cathodes and anodes, and insufficient interfacial solid‐to‐solid contact.^[^ ^18^, ^19^, ^20^, ^21^, ^22^ ^]^ Addressing these challenges through materials innovation and engineering strategies is essential for the widespread adoption of ASSLIBs and their realization as a cornerstone of sustainable energy systems.

At the same time, cathode materials play a critical role in achieving high energy density in ASSLIBs. For example, the commercialized layered cathode material LiCoO_2_ (LCO) offers a practical specific capacity ranging from 140 to 180 mAh g^−1^ depending on the upper cut‐off voltage (4.2–4.45 V). This results in a specific energy density of up to 720 Wh kg^−1^,^[^ ^23^, ^24^ ^]^ offering a distinct advantage in the ASSLIB applications. However, from an economic perspective, cathode materials account for approximately half of the total material cost in conventional liquid LIBs, and the cost of expensive and essential components, such as cobalt (Co), contributes up to 30% of the cathode cost.^[^ ^25^, ^26^, ^27^ ^]^ Therefore, the high cost of LCO cathode, as much as 55 US$ kg^−1^,^[^ ^28^ ^]^ inevitably increases the overall cost of ASSLIBs. In addition, with the escalating concerns over the scarcity and toxicity of Co, the research interest has shifted to the rapidly developed high‐performance and low‐Co layered LiNi_1‐x‐y_Co_x_Mn_y_O_2_ (NCM).^[^ ^29^, ^30^ ^]^ Notably, Ni‐rich NCM cathodes, for example, NCM811, with Ni contents higher than 80% among the TMs, possess a practical specific capacity of >200 mAh g^−1^ and an energy density of 780 Wh kg^−1^.^[^ ^31^, ^32^, ^33^ ^]^ Its high discharge capacity positions it as the leading cathode material in current ASSLIBs.^[^ ^30^, ^34^, ^35^ ^]^ Notably, the higher Ni content in NCM cathodes, while beneficial for energy capacity, not only poses additional challenges related to thermal stability and degradation under high‐voltage cycling but also remarkably increases material costs.^[^ ^27^, ^36^, ^37^, ^38^ ^]^ Nevertheless, the pursuit of cathode materials with higher energy density also drives up the overall cost of the ASSLIBs.^[^ ^39^ ^]^ Therefore, to realize the cost‐effectiveness of the ASSLIBs, it is essential to spur renewed interest in Co‐free and low‐Ni cathode materials with Mn as the dominant element.^[^ ^40^ ^]^ According to a report published in 2016,^[^ ^41^ ^]^ Mn is significantly more abundant in the Earth's crust, with a concentration of 1100 ppm, compared to Ni at 90 ppm and Co at 30 ppm, resulting in higher annual mine production and lower associated costs for Mn‐based products. Furthermore, the cost of Mn metallurgical ore is only 1.81 US$ kg^−1^, less than ca. 20 US$ kg^−1^ of Ni and ca. 33 US$ kg^−1^ of Co metallurgical ore (Figure 1B).^[^ ^42^ ^]^ Consequently, the material cost for LiMn_2_O_4_ (LMO) and LiNi_0.5_Mn_1.5_O_4_ (LNMO) is 12 US$ kg^−1^ and 21 US$ kg^−1^, respectively, in contrast to LCO (55 US$ kg^−1^).^[^ ^28^ ^]^ A comparison of the material cost estimation required for ASSLIBs is calculated and shown in Figure 1C. By idealizing the cost of the SSEs as the threshold of 50 US$ kg^−1^,^[^ ^15^, ^39^, ^43^ ^]^ the total material costs of ASSLIBs using the typical spinel and layered cathodes are shown on the left (excluding the overhead, engineering, processing, and miscellaneous costs). The total materials cost of LCO‐based ASSLIBs is ≈123 US$ kWh^−1^, which is more than double the price of LMO‐ and LNMO‐based ASSLIBs, at the cost of 40 and 48$ kWh^−1^, respectively. The high Ni layered cathode‐based ASSLIBs, for example, NMC 9.5.5, are estimated to be more than 60$ kWh^−1^, which is noticeably higher than spinel cathode counterparts. In addition, the corresponding material costs of three typical SSEs: oxide‐, sulfide‐, and halide‐based ASSLIBs with LMO cathodes are presented on the right in Figure 1C, where different colors for SSEs indicate the range of cost variations of each SSEs. With LMO as the cathode, the total cost of the ASSLIBs is significantly escalated due to the cost‐intensive nature of the SSEs. Even with the most cost‐effective sulfide SSEs, which exhibit the price ranges of 45–106 US$ kWh^−1^,^[^ ^15^ ^]^ the cost of the ASSLIBs ranges from 80 to 140 US$ kWh^−1^. Therefore, reducing the cost of the cathode is nevertheless important and necessary in accelerating the commercialization of the ASSLIBs. Additionally, the weight ratio of each element in typical cathodes is further compared as shown in Figure 1D. A high Co content of as high as 60.21 wt.% in the LCO cathode elucidated the root cause of its high material costs. In NMC 811 and NMC 9.5.5 cathodes, even with the competitive and low weight percentage of Co and Mn, the high Ni (≈50 wt.%) drives the prices noticeably higher than those of spinel cathodes. Moreover, the critical challenges persist, including the dramatically increased demands of the necessary elements, the limited metal resources, and the elevated mineral prices. As shown in Figure 1E, the price of manganese mineral gradually increased from 0.32 × 10^4^ US$/metric ton in 2015 to 0.58 × 10^4^ US$/metric ton in 2024. In contrast, the price of cobalt and nickel minerals dramatically elevated from 2.96 × 10^4^ US$/metric ton and 1.18 × 10^4^ US$/metric ton to 3.75 × 10^4^ US$/metric ton and 1.70 × 10^4^ US$/metric ton from 2015 to 2024, respectively. Concomitantly, the uneven worldwide distribution of the mineral reserves, as presented in Figure 1F, shows that 53% and 15% of cobalt reserves are located in the Democratic Republic of Congo and Australia, respectively. Whereas manganese mineral reserves are more dispersed across South Africa, Australia, China, and Brazil. Those abovementioned challenges have posed extreme threats to the feasibility and availability of cobalt mineral, thus further underscoring the urgent need to transition from Co‐contained cathodes to sustainable and cost‐effective Mn‐based spinel cathodes.

In addition, because of their unique spinel structure, low cost, abundance, and high discharge capacity, LMO and high‐voltage LNMO cathodes have garnered significant interest as promising candidates. Spinel Mn‐based cathodes feature 3D fast Li‐ion transport channels and exhibit exceptional thermal and electrochemical stability, which has already demonstrated excellent performance in liquid LIBs.^[^ ^48^, ^49^, ^50^, ^51^ ^]^ For instance, a porous LMO nanorod cathode demonstrated excellent fast‐charging capability, delivering an initial discharge capacity of 105 mAh g^−1^ under 10C, with a capacity retention of 86% after 500 cycles.^[^ ^52^ ^]^ A crystallographic‐site structure modified LNMO cathode delivered excellent long cycling stability with a capacity retention of 72.4% after 3000 cycles under 1C.^[^ ^53^ ^]^ These inspiring results reaffirmed the promising capability of Mn‐based cathode materials. Despite their distinguished potential, several challenges hinder the practical application of Mn‐based cathodes in ASSLIBs, limiting their ability to achieve optimized electrochemical performance. Key issues include transition metal (TM) dissolution and diffusion, inadequate solid‐solid interfacial contact, and side reactions at the cathode/SSEs interface.^[^ ^54^, ^55^, ^56^, ^57^ ^]^ Particularly, the incompatibility between SSEs with a narrow electrochemical stability window and spinel Mn‐based cathodes with high operating voltages remains a significant obstacle. Addressing these critical challenges is imperative to promote the electrochemical performance of Mn‐based cathodes in ASSLIBs.

**Figure 2:**
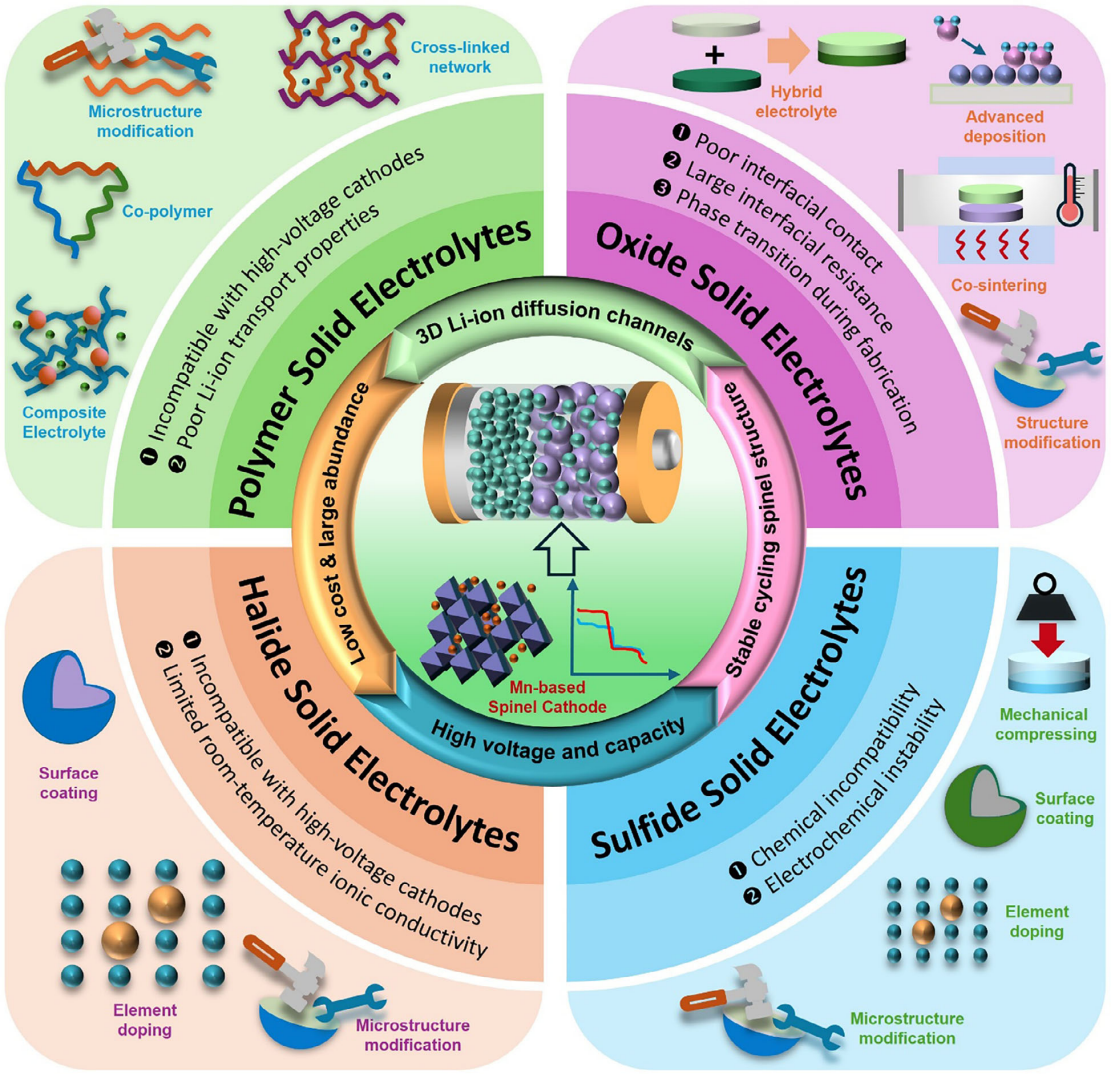
The challenges of Mn‐based spinel cathodes with various solid‐state electrolytes in the applications of ASSLIBs and their corresponding mitigation strategies.

To gain a comprehensive understanding and provide valuable insights into the rational design and implications for the future development of Mn‐based cathodes in ASSLIBs, it is indispensable to summarize their challenges, recent advancements, and future perspectives. This work begins by introducing the characteristics of Mn‐based spinel structures along with their electrochemical behaviors and structural degradation mechanisms. Subsequently, the current advancements and the challenges associated with Mn‐based cathodes paired with various types of SSEs are discussed, including polymer, oxide, polymer/oxide composite, sulfide, halide, and LiPON thin film SSEs. The strategies to mitigate the corresponding challenges are thoroughly examined. The aforementioned challenges of each type of SSEs when paired with LMO/LNMO cathodes and the strategies to mitigate them were exclusively summarized in **Figure** **2**. In the final section, a comprehensive conclusion is provided, along with perspectives on potential strategies to further optimize Mn‐based spinel cathodes for next‐generation ASSLIBs applications.

## 2 Mn‐Based Spinel Cathodes: Structure, Performance, and Degradation

### 2.1 Spinel LiMn_2_O_4_ Cathodes

As early as 1981, for the first time, Thackeray et al.^[^ ^58^, ^59^ ^]^ studied the electrochemical behavior of the spinel structure Mn_3_O_4_ at room temperature and reported that the Li‐ions could be chemically and electrochemically inserted into the spinel structure Li(M_2_)O_4_ (M = Fe or Mn) while maintaining the spinel structure unperturbed. In a subsequent study,^[^ ^60^ ^]^ Thackeray and Goodenough found that Li(Mn_2‐x_M_x_)O_4_ (x < 0.1, M = Li^+^, Ni^+^, and/or Al^3+^) cathodes could undergo a reversible lithium extraction at 4.0 V vs Li/Li^+^ while retaining the spinel structure, which further confirmed the successful extraction of lithium from LiMn_2_O_4_. These results indicated the potential of utilizing spinel structure materials as cathodes in LIBs. Attracted by the fast and facile 3D Li‐ion diffusion channels within the spinel framework, Goodenough suggested further and deeper research on lithium manganese oxide (LiMn_2_O_4_/LMO).^[^ ^60^, ^61^ ^]^

The electrochemical properties and the cycling behavior of LiMn_2_O_4_ in a full battery were first tested by Tarascon and coworkers in 1991.^[^ ^62^ ^]^ At the average voltage of 4.1 V, a reversible 0.4 Li‐ion insertion per Mn for LiMn_2_O_4_ or Li‐leached LiMn_2_O_4_—λ‐MnO_2_ was observed. The battery retained over 90% of its capacity after 50 cycles and delivered an energy density of 480 Wh kg^−1^.

#### 2.1.1 Key Features of the Spinel Structure

Typically, LMO possesses a cubic spinel structure belonging to the space group of $\mathrm{Fd}\bar{3}m$. Li‐ions reside at 8a tetrahedral sites and fill 1/8 of the sites. Oxygen atoms are located at 32e sites in a face‐centered cubic (FCC) close‐packed arrangement, and Mn‐ions are arranged at the octahedral 16d sites while leaving the other half of the octahedral 16c sites unoccupied. This octahedral void at 16c sites is located halfway between face‐shared (LiO_4_)‐centered tetrahedral 8a sites and corner‐shared (MnO_6_)‐centered 16d octahedral sites, which provides a strong bonded 3D pathway of 8a‐16c‐8a for fast Li‐ions transportation.^[^ ^34^, ^63^, ^64^, ^65^ ^]^ It is also suggested that this corner‐shared neighboring structure provides longer Li‐TM distances and is, hence, more energetically stable due to the reduced cationic Coulombic repulsion.^[^ ^66^ ^]^

**Figure 3:**
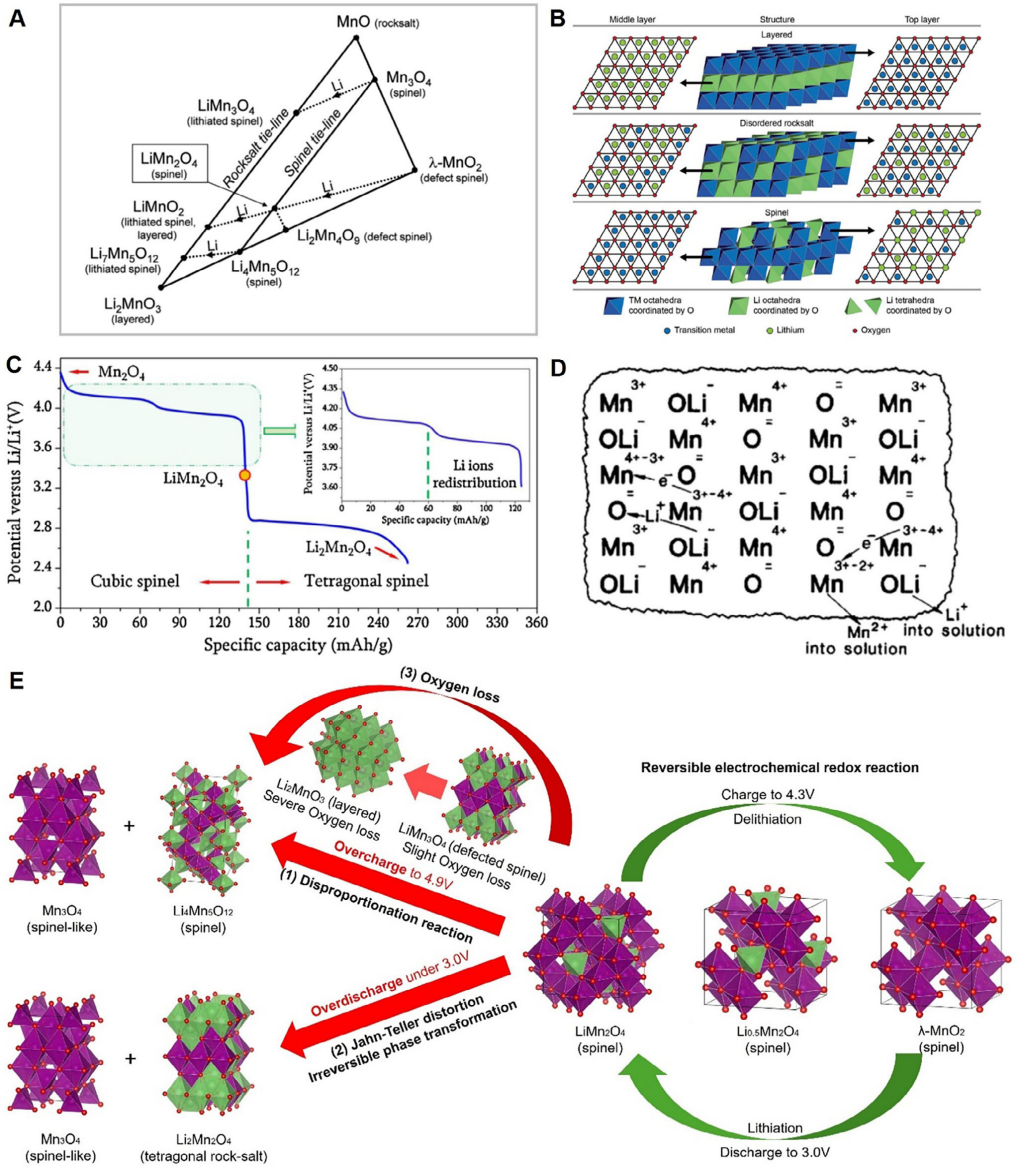
A) Phase diagram of different L‐M‐O compositions. Reproduced with permission.^[^ ^68^ ^]^ Copyright 2018, The Royal Society of Chemistry. B) Schematic illustrations of the relationship between spinel, disordered rocksalt, and layered structures. Reproduced with permission.^[^ ^71^ ^]^ Copyright 2017, Wiley. C) LMO discharge profile from 4.3 V to 2.4 V. Reproduced with permission.^[^ ^134^ ^]^ Copyright 2015, Elsevier. D) Schematic representation of LMO to MnO2 conversion in acidic aqueous solution. Reproduced with permission.^[^ ^99^ ^]^ Copyright 1981, Elsevier. E) Schematic of LMO charge‐discharge mechanism and the irreversible phase transformation during overcharge/overdischarge.

The Mn‐ion typically exhibits +4 and +7 oxidation states, whereas in LiMn_2_O_4_, an equal proportion of Mn^3+^ and Mn^4+^ resulted in an average valence of +3.5. Lithium‐manganese‐oxide (Li‐Mn‐O) compounds could possess various phases, as demonstrated in the phase diagram in **Figure** **3**A.^[^ ^67^, ^68^ ^]^ Spinel LiMn_2_O_4_ lies on the “spinel tie line” and at the center of the triangle phase diagram. Depending on the content of Li and the valence of Mn, LiMn_2_O_4_ can transform into other spinel‐related structures. For example, the dashed line that connects λ‐MnO_2_, LiMn_2_O_4_, and LiMnO_2_ illustrates the transformation of Li_x_Mn_2_O_4_ (0 < x < 2) during the entire Li‐ion extraction and insertion process. The arrows on the dashed line indicate the complete process of Li‐ions insertion, where λ‐MnO_2_ is converted to LiMn_2_O_4_ and eventually to LiMnO_2_. Consequently, the completely lithiated LiMn_2_O_4_ results in either a tetragonal rock‐salt or a monoclinic layered structure, which is also known as Li_2_Mn_2_O_4_, also referred to as LiMnO_2_.^[^ ^59^, ^68^, ^69^, ^70^ ^]^

It is worth noting that “spinel” and “rock‐salt” structures possess similarities, such as sharing the same face‐centered cubic sublattice. However, the main difference between these two structures is the cation occupation: in spinel structures, half of the cations occupy octahedral sites and the other half occupy tetrahedral sites, whereas in rock‐salt structures, the cations only occupy the octahedral sites (Figure 3B).^[^ ^34^, ^71^ ^]^ This partially irreversible phase transformation between spinel and rock‐salt structures is the origin of Mn‐dissolution and will be discussed in detail in the following section. Moreover, to construct a stable and structured spinel cathode material, the ratio between the transition metal (Mn) and O plays a decisive role in determining stability. Within the spinel cathode material, the higher the ratio of TM:O, the poorer the electrochemical performance, especially when the ratio is larger than 0.5, such as in Mn_3_O_4_ (MnMn_2_O_4_). This phenomenon originates from oxygen evolution at high voltages, lower average Mn valency (<+3), and the occupation of Mn in the tetrahedral sites in addition to the octahedral sites, which hinders Li‐ion diffusion and transfer. In contrast, LiMn_2_O_4_ and LiNi_0.5_Mn_1.5_O_4_, with a robust spinel structure with a TM:O ratio of less than 0.5, demonstrate promising stable structures during electrochemical cycling in ASSLIBs.^[^ ^55^ ^]^

##### Electrochemical Behaviors

During the half‐delithiation process (i.e., 1 > x > 0.5), Li_x_Mn_2_O_4_ possesses a single homogeneous cubic phase with continuously increasing lattice parameters up to the voltage of 3.95 V. In an ideal situation, half of the lithium ions, which reside in the 8a tetrahedral sites, would participate electrochemically, leaving the other half to yield Li_0.5_Mn_2_O_4_.^[^ ^60^, ^72^, ^73^, ^74^ ^]^ The detail of the electrochemical characteristics at different Li‐ion concentrations is listed in **Table** **1**. In the practical applications, the spinel cathodes Li_x_Mn_2_O_4_ are generally presented in the status of x = 1 (LiMn_2_O_4_) as a pristine cathode. The typical LiMn_2_O_4_ cathode‐based ASSLIBs are electrochemically tested with charging as the first step, and this matches the reported literature. Therefore, the first emerged cubic phase within 1 > x > 0.5 during the charging process is defined as phase **(I)**. Accordingly, the following observed cubic phase is defined as phase **(II)**. Theoretically, during this half‐delithiation, the Li_x_Mn_2_O_4_ exhibits a single cubic phase **(I)** and could provide half of its theoretical specific capacity (148 mAh g^−1^), thus delivering 74 mAh g^−1^. When x falls between 0.5 > x > 0.3, while maintaining the cubic phase **(I)**, another cubic phase, which is recognized as phase **(II),** emerges. Therefore, Li_x_Mn_2_O_4_ exhibits two identical cubic phases (**I + II**) with different lattice parameters, which normally occurs at approximately 4.11 V. Eventually, only one cubic phase with a smaller lattice, phase **(II)**, is observed in the region where 0.3 > x > 0.^[^ ^75^, ^76^ ^]^ The lithiation of Li_x_Mn_2_O_4_ (x from 0 to 1) is considered a reversible delithiation process. Kuwata's group utilized in situ Raman^[^ ^77^, ^78^ ^]^ to study the structural change and Li‐ion transport within LMO all‐solid‐state thin‐film batteries.^[^ ^79^ ^]^ A reversible phase transition between α (LiMn_2_O_4_), β (Li_0.5_Mn_2_O_4_), and λ (Mn_2_O_4_) phases^[^ ^80^ ^]^ during the charging and discharging processes was characterized. During the charging process, the Li‐ions de‐intercalated from LMO and migrated to the Li anode. It was observed that the phase transitions from α to β occurred when the potential was increased to 4.3 V. Further charging to 4.4 V facilitated the phase transition from β to λ. The discharging process was the reverse of the charging process, but with a faster rate of phase change compared to the charging process. The corresponding Li‐ion chemical diffusion coefficients that were derived from the electrochemical measurements also aligned with the diffusion simulations. It is extremely important to note that Li_x_Mn_2_O_4_ could maintain a stable cubic structure throughout the entire lithium insertion and extraction process, highlighting its structural stability during cycling in ASSLIBs.

**Table 1:** Electrochemical characteristics as a function of Li‐ion concentration x in Li_x_Mn_2_O_4_ (0 ≤ x ≤ 2) at room temperature.

x Range	0–0.3	0.3–0.5	0.5–1	1–2
Voltage [V] vs Li/Li^+^	4.11	4.00	3.95	2.96
Phase	1 cubic phase (II) $\mathrm{Fd}\bar{3}m$	2 cubic phases (I + II) $\mathrm{Fd}\bar{3}m$	1 cubic phase (I) $\mathrm{Fd}\bar{3}m$	cubic + tetragonal phases $\mathrm{Fd}\bar{3}m+14_{1}/\mathrm{amd}$
Capacity [mAh g^−1^]	74		74	100

Generally, the operating voltage window of LMO‐based ASSLIBs is restricted between 3.0 V and 4.3 V to avoid the Jahn‐Teller distortions (which will be discussed in detail in the following section) and maintain structural stability.^[^ ^81^, ^82^ ^]^ In a typical electrochemical behavior of the LiMn_2_O_4_ plot during the charging and discharging process, the observed 2 plateaus in the charging process correspond to the 2 half‐lithiations at the voltage of approximately 4.04 V and 4.16 V, respectively. Alternatively, the two plateaus occur at around 4.0 V and 4.11 V during the discharging process, reflecting the Mn reduction reactions. The differential capacity graph and CV plots^[^ ^83^, ^84^, ^85^ ^]^ further evidence the occurrence of these reactions, as the phase transitions of Li_x_Mn_2_O_4_ during the oxidation and reduction are clearly indicated at the corresponding peaks.

Furthermore, when continuously inserting Li‐ions into Li_x_Mn_2_O_4_, the lithiation process (i.e., 1 < x < 2) induced LMO reduction reactions to occur at 2.96 V; meanwhile, full occupation of Li‐ions at the 8a sites, and insertion into 16c sites were observed. During this two‐phase reaction, the co‐existence of spinel cubic phase LiMn_2_O_4_ and rock‐salt tetragonal Li_2_Mn_2_O_4_ is commonly observed.^[^ ^74^ ^]^ Meanwhile, the typical discharge profile during the whole Li intercalation process (0 < x < 2) is illustrated in Figure 3C. It could also be observed that two voltage drops (0.15 V and >1 V) were identified between the three plateaus, which were more pronounced between LiMn_2_O_4_ and Li_x_Mn_2_O_4_ (1 < x < 2). This phenomenon originated from the high sensitivity of the redox couple Mn^4+^/Mn^3+^ to Li^+^ ordering.

##### Structural Degradation Mechanism

The electrochemical performance and the cycle life of the ASSLIBs closely and critically rely on the structural integrity of cathodes.^[^ ^86^ ^]^ Even after decades of tremendous efforts aimed at understanding the structural degradation mechanism of LMO, the electrochemical performance of LMO‐based ASSLIBs remains significantly hindered by these challenges. The Mn dissolution/diffusion that causes the loss of Mn during battery cycling is a well‐discerned challenge for Mn‐based spinel cathodes.^[^ ^87^ ^]^ Typically, when paired with polymer‐based SSEs, Mn dissolution begins with the Mn‐ions dissolving from the cathode active material, followed by migrating through the working SSE(s) and depositing onto the paired anode.^[^ ^88^, ^89^, ^90^, ^91^, ^92^ ^]^ Meanwhile, when coupled with inorganic‐based SSEs encompassing oxide^[^ ^20^, ^93^ ^]^ and sulfide,^[^ ^94^ ^]^ instead of dissolution, the interdiffusion between the TMs more severely deteriorates the performance of the ASSLIBs. The associated battery degradation and capacity fading after prolonged cycling are not only derived from the active material lost but also from the increased interfacial impedance. The underlying mechanism of structural degradation could be outlined by 1) disproportionation reaction, 2) interface instability at high voltage, and 3) undesirable phase transformation.^[^ ^95^, ^96^, ^97^, ^98^ ^]^ Here, we will discuss these three primary challenges contributing to the structural degradation of LiMn_2_O_4_ in ASSLIBs.

##### Disproportionation Reaction

The Mn disproportionation reaction mechanism was proposed by Hunter^[^ ^99^ ^]^ even prior to LiMn_2_O_4_ being applied in the LIBs. Previous studies have delineated that during the LiMn_2_O_4_ chemical‐delithiation process in acidic solutions, the removal of the Li^+^ and Mn^2+^ from the surface resulted in the increasing number of Li‐ions diffusing from the bulk to the surface and associated with electron hopping. Consequently, the bulk LMO turned into λ‐MnO_2_ with all the Mn maintained as +4, as shown in Figure 3D. This process was interpreted as 2Mn^3+^ = Mn^2+^ + Mn^4+^. Later on, Thackeray et al.^[^ ^60^ ^]^ modified the disproportionation reaction to Mn^3+^_surface_ + Mn^3+^_bulk_ = Mn^2+^_surface_ + Mn^4+^_bulk_ based on the room‐temperature lattice diffusivity of the Li‐ion and electrons. Truly, the phenomenon of the oxidation state Mn^2+^ dissolved in the electrolyte was observed and confirmed experimentally with differential pulse polarography^[^ ^100^ ^]^ and high‐sensitivity X‐ray absorption fine structure spectroscopy.^[^ ^101^ ^]^

Interestingly, some current studies have raised the controversy of whether Mn^2+^ or Mn^3+^ is the main dissolution species. Halalay et al.^[^ ^102^, ^103^ ^]^ quantitatively analyzed the Mn dissolution within LMO‐based LIBs with a combination of advanced characterization techniques (EPR, AAS, ICP, and XANES). From the observation of the powder‐dissolution experiments under 50 °C, 70–75% of the dissolved Mn from LMO was Mn^3+^. The findings substantiated that the Mn^3+^‐ions were the dominating soluble species dissolved from LMO instead of the conventional Mn^2+^‐ion. This observation was attempted to be explained by Mn^3+^ exhibiting greater solvation stability than Mn^2+^ during the slow disproportionation reactions. This finding still requires further research to delve into the intricacy of the Mn dissolution mechanism.

In addition, some experimental observations still possess discrepancies with the conventional disproportionation reaction mechanism and premise. According to the disproportionation mechanism, the Mn dissolution should be more severe with the decrease of the Mn valence. Therefore, it should be perceived that the Mn dissolution is exacerbated at the end of the discharging process, where Mn possesses the lowest valence. Meanwhile, when x ≈ 0, the fully delithiated Li_x_Mn_2_O_4_ (λ‐MnO_2_) should suffer minimally from Mn dissolution, since all the Mn possesses a 4+ valence. However, when compared with previous experimental results,^[^ ^100^, ^101^, ^104^, ^105^, ^106^, ^107^ ^]^ it was underlined that Mn dissolution was more pronounced at both the high voltage above 4.1 V and the low voltage below 3.1 V.^[^ ^108^ ^]^ From the AC impedance study, Oh et al.^[^ ^100^ ^]^ revealed that Mn dissolution was not the major source responsible for the capacity loss in LiMn_2_O_4_/Li LIBs. It was found that Mn‐dissolution was responsible for approximately 20%∼30% of the overall capacity loss. In contrast, the largely developed and accumulated resistance within the cathode severely reduced the interfacial contact area and deteriorated the electrochemical performance. This was also reaffirmed by simulation^[^ ^109^ ^]^ and through experimental results.^[^ ^106^, ^108^ ^]^ Therefore, when thoroughly dissecting the root cause and mechanism of Mn dissolution, it should be discerned that the disproportionation reaction of Mn may not be the only cause.

##### Interface Instability

Mn diffusion/TM interdiffusion originates from the interface instability‐derived decomposition reactions. However, due to the inherent thermodynamic and chemical properties of the SSEs, the Mn diffusion/TM interdiffusion triggering conditions are diverse. When coupled with kinetic‐inhibited oxide‐based SSEs, the interface is relatively stable at room temperature since the interactions with oxide SSEs do not stimulate the decomposition reactions by simply mixing, due to insufficient kinetic energy and/or interfacial contact area.^[^ ^20^, ^110^, ^111^, ^112^ ^]^ Therefore, the intrinsic rigid mechanical properties of oxide SSEs require high‐temperature cosintering or treatments to achieve intimate contact with cathodes. The TM interdiffusions become significant at high temperatures and are more pronounced with the elevation of the temperature. The decomposition reactions at the interface form thermodynamically favorable Li‐free by‐products that could hinder Li‐ion transport and charge transfer, subsequently increasing the interfacial impedance and deteriorating the performance of ASSLIBs. Meanwhile, the mutual diffusion phenomenon occurs at room temperature when paired with sulfide electrolytes.^[^ ^21^, ^94^, ^113^, ^114^ ^]^ The side reactions lead to the decomposition of cathodes and/or sulfide SSEs and generate insulating by‐products that continuously thicken the interphases and amplify the impedance.

From the structural perspective, the delithiated LMO cathodes are more likely to lead to interface instability and induce anion‐redox reactions that result in oxygen loss and oxygen global mobility, the reduction of TMs, and side reactions with electrolytes.^[^ ^115^ ^]^ In general, at high voltages (>4.3 V vs Li/Li^+^), the oxidation reaction of oxygen O^2−^ accelerates the mobility of oxygen and the rates of oxygen escaping from LMO. Ben et al.^[^ ^72^ ^]^ investigated the electrochemically cycled LMO cathode and observed a 5–6 nm thick distorted layered‐like composition at the surface regions. The formation of the surface layer was triggered by oxygen loss during the charge‐discharge cycling, which resulted in the valence of Mn being reduced from +4 to +3 due to charge compensation, thus leading to the structural transformation of Mn migration to lithium tetrahedral sites and formed defect‐spinel LiMn_3_O_4_ (when the amount of oxygen loss was small) and the continuous migration eventually derived to layered Li_2_MnO_3_. Both surface structure distortion and oxygen loss contributed to the Mn dissolution from the cathode to the electrolyte, severely hindering Li‐ion transportation and increasing the impedance. Consequently, the oxygen vacancies, TM migration, and structural degradations result in capacity decay and electrolyte decomposition.

Through surface‐sensitive XPS and HR‐EELS/STEM, Tang et al.^[^ ^83^ ^]^ observed that the Mn‐ions at the surface of the LMO electrode reduced their valence during the charging process and increased when discharging, which was in contrast to the behavior of the Mn‐ions in bulk. The chemical and structural evolution of the LMO at the surface was elaborated by the oxygen loss during cycling, and accordingly, formed the Mn_3_O_4_ phase. Such a phase contained 1/3 tetrahedral Mn^2+^ ions, which significantly contributed to the Mn dissolution into the electrolyte and the loss of the cathode active material. It was also observed that the fraction of Mn_3_O_4_ reached a maximum at the end of charge, gradually decreased during discharge, and was negligible when discharged to 3.0 V vs Li/Li^+^. The mechanism of this phenomenon was unclear in this study; however, the reversible reactions from Mn_3_O_4_ back to Li_x_Mn_2_O_4_ were considered impossible due to the loss of oxygen. Thus, the decomposition reactions into either electrolyte‐soluble components or λ‐MnO_2_ were adopted to attempt to explain this observation.

In addition, the charge compensation of Li‐ion removal forms localized electron holes on the Mn^4+^ and Li^+^ coordinated oxygen atoms. The ionic interactions between O and Mn/Li promote the localization of O instead of the formation of O_2_^2−^‐peroxide species.^[^ ^116^ ^]^ It was confirmed that the surface instability of Mn‐based spinel cathodes was further aggravated under highly delithiated states.^[^ ^83^, ^117^, ^118^, ^119^ ^]^ Thus, the composition reconstruction was observed to be more pronounced at the surface than at the bulk. Notably, the redox couples such as Co^4+^/Co^3+^ in LiCoO_2_ are more prone to surface instability due to the oxygen being pinned at the top of the 2p bands. The susceptibility to the surface structural instability of LMO strongly suggests that anion‐redox‐induced oxygen loss, TM dissolution, and surface reconstruction occurred regardless of the material's structure, but is more likely under high voltage.

##### Undesirable Phase Transformation

Phase transformation is another remarkable challenge that hinders the electrochemical performance of the LMO‐based ASSLIBs. The increased impedance is unfavorable for fast Li‐ion transport and consequently induces capacity fading.

During the lithiation process from LiMn_2_O_4_ to lithiated Li_1+x_Mn_2_O_4_ (with x > 0), the equal proportioned redox couple Mn^3+^/Mn^4+^ transformed into (1+x) Mn^3+^ and (1‐x) Mn^4+^. The Mn^3+^ with an electronic configuration of t_2g_^3^e_g_^1^ (high‐spin complexes) was considered a Jahn‐Teller ion. Meanwhile, the low‐spin complex Mn^4+^ with t_2g_^3^e_g_^0^ electronic configuration is not. Therefore, the continuous lithiating leads to the accumulation of Mn^3+^ exceeding ½ of the total Mn (also recognized as Mn disproportionation). This electrochemical lithium insertion process involves a two‐phase reaction associated with an anisotropic distortion, especially more pronounced when the average valency of manganese drops below 3.5+. Consequently, the Li‐ions that originally occupied the tetrahedral sites within the cubic structure LiMn_2_O_4_ are relocated to the octahedral sites in the newly formed ordered rock‐salt Li_2_Mn_2_O_4_. Notably, the partially irreversible phase transformation from cubic to tetragonal rock‐salt occurred at the end of the charging and discharging process, which is more significant under fast charging/discharging and in non‐equilibrium conditions, inducing the generation of Mn_3_O_4_ and Li_2_Mn_2_O_4_. These formed by‐products severely hinder the transport of Li‐ions and dramatically increase the impedance. On the other hand, when LiMn_2_O_4_ experiences delithiation in the charging process, the migration of Li‐ions from cathode to anode induces the valence of Mn^3+^ to increase to Mn^4+^ and forms λ‐Mn_2_O_4_. Even though the solid‐solution reaction could maintain the stable cubic structure without distortions, and Mn^4+^ is not a Jahn‐Teller ion as mentioned above, the oxygen escape promotes the formation of Mn_3_O_4_ at the surface and thus accelerates Mn dissolution.^[^ ^120^, ^121^, ^122^, ^123^, ^124^, ^125^, ^126^ ^]^

To mitigate the degradation pathway, the operating voltage window is generally maintained between 3.0 V and 4.3 V (vs Li/Li^+^) in the LiMn_2_O_4_‐based LIBs, to avoid the unfavorable Jahn–Teller distortion‐induced phase transformation. The stable reversible electrochemical redox reaction of LMO cathodes is shown in Figure 3E. The associated challenges and phase transformations at overcharge/overdischarge when the operating voltage is up to 4.9 V/under 3.0 V (vs Li/Li^+^) are also demonstrated. However, even within this potential window, phase transformation is still inevitable. Thackeray et al.^[^ ^123^ ^]^ systematically unraveled the phase transformation‐induced structural changes through high‐resolution electron diffraction and imaging for discharged 4.0 V vs Li/Li^+^ Li_x_Mn_2_O_4_/Li cells. At the surface of the cells, a rock‐salt structure of Li_2_Mn_2_O_4_ was observed. In Ferreira's work,^[^ ^127^ ^]^ a stable and thin Mn^2+^(Mn_2_^3+^)O_4_ (Mn_3_O_4_) phase was observed at the surface of the uncycled LMO; meanwhile, another Li_1+x_Mn_2_O_4_ (0 < x < 1) phase was formed at the sub‐surface of the bulk LMO.

To conclude, the challenges discussed above undoubtedly negatively impact the electrochemical performance of the LiMn_2_O_4_‐based ASSLIBs. It should be noted that Mn dissolution is also profoundly affected by various other factors, such as particle size, specific contact area,^[^ ^100^, ^105^ ^]^ temperature,^[^ ^104^, ^107^, ^123^ ^]^ oxygen vacancies, lithium defects, and manganese antisites,^[^ ^128^, ^129^, ^130^, ^131^, ^132^, ^133^ ^]^ etc. These research findings provide extremely valuable insights into the rational design of LiMn_2_O_4_‐based ASSLIBs. Constructing a phase‐stable surface is the pragmatic strategy to suppress the above‐mentioned challenges; therefore, endeavoring to achieve superior electrochemical performance.

### 2.2 High‐Voltage LiNi_0.5_Mn_1.5_O_4_ Cathodes

LiNi_0.5_Mn_1.5_O_4_ (LNMO), a Ni‐doped LMO spinel structure material, which was first reported by Blasse in 1964,^[^ ^135^ ^]^ has garnered significant interest for its high operating voltage. Within the LNMO structure, the Ni‐ions possess a +2 valence, inducing all the Mn‐ions to exhibit a +4 valence. The difference in charge and ionic radii between Ni^2+^ (69 pm) and Mn^4+^ (53 pm) results in the occupation of a 1:3 order on octahedral sites. Therefore, instead of a Ni‐Ni structure, each Ni^2+^‐ion connects and is surrounded by Mn^4+^‐ions, resulting in a lower lattice enthalpy superstructure. Hence, the paramagnetism of the Ni^2+^ and Mn^4+^ at octahedral sites, along with the crystallographic ordering and cation ordering between ions, leads to the LNMO ferrimagnetic behavior.^[^ ^135^, ^136^ ^]^ In 1991, beyond the electrochemical properties study of LMO, Tarascon et al.^[^ ^62^ ^]^ systematically tested and analyzed the materials with different degrees of cation substitution in Li_x_M_y_Mn_2‐y_O_4_ (M = Ti, Ni, Zn, etc.) in the applications of LIBs. When discharged under 3.0 V, the Ni‐substituted materials demonstrated one 2.8 V plateau, the same as the unsubstituted LMO material, and one additional 2.2 V discharge plateau. The two plateaus distinguished the cubic to tetragonal phase transitions from 2.8 V to 2.2 V. In addition, there was no observation of increasing capacity with the Ni substitution.^[^ ^137^, ^138^, ^139^ ^]^ In the later work of Amine et al.,^[^ ^140^ ^]^ it was revealed that during the charge and discharge process between Li_1+x_Ni_0.5_Mn_1.5_O_4_ (0 < x < 1) and LiNi_0.5_Mn_1.5_O_4_, the demonstrated cycling stability at 3 V was attributed to the presence of Ni, which enhanced the bulk structure and the stable cubic spinel structure without structural transitions. More significantly, the Ni‐doped spinel cathode possessed Ni^2+,^ resulting in a higher Mn^4+^ content than LMO. The disproportionation reaction derived from Mn‐dissolution was subsequently suppressed since the observed dissolution was much slower than the LMO cathode material. In the work of Dahn et al.,^[^ ^141^ ^]^ the removal of Li^+^ within LiNi_0.5_Mn_1.5_O_4_ at the voltage of 4.7 V was demonstrated. By comparing the different compositions of LiNi_x_Mn_2‐x_O_4_ (0 < x < 0.5), it was determined that the increasing capacity of the 4.7 V plateau, which corresponded to Ni^2+^ to Ni^4+^ oxidation, was at the cost of a decrease in the 4.1 V Mn^3+^ to Mn^4+^ plateau. Consequently, the total capacity remained consistent with LMO, which was aligned with the previous result of Tarascon et al.^[^ ^62^ ^]^ discussed above. Additionally, from the differential capacity studies, when the maximum of x (x = 0.5) was achieved, the emerged double peaks centered at 4.7 V replaced the peak at 4.1 V completely, which profoundly emphasized the dominance of Ni^2+^/Ni^4+^ redox reaction within LNMO. These works significantly suggested the potential of realizing a high‐voltage spinel cathode. All these findings and intensive research from previous studies significantly pushed the understanding and development of LNMO to a rapid pace.

#### 2.2.1 Ordered and Disordered Spinel Structures

Generally, for the spinel structure LNMO, there are two different crystal polymorphs: cation‐ordered phase (O‐LNMO) and cation‐disordered phase (D‐LNMO). The cation‐ordered phase, which belongs to space group P4_3_32, possesses Ni and Mn ions in 4a and 12d octahedral sites, respectively. The Li‐ions are located at 8c sites, while O‐ions occupy 8c and 24e sites. In contrast, within the space group $\mathrm{Fd}\bar{3}m$, the cation‐disordered phase randomly accommodates Mn and Ni in 16d octahedral sites, and Li‐ions and O‐ions are located at 8a and 32e sites, respectively.^[^ ^54^, ^142^, ^143^ ^]^ Moreover, in contrast to the cubic P4_3_32 phase, within the high symmetry face‐centered $\mathrm{Fd}\bar{3}m$ cubic phase, the extinction of (100), (110), (320), (510), and (522) peaks in the micro‐region were remarkably observed in the XRD patterns, which originated from the phase transformation from O‐LNMO to D‐LNMO.^[^ ^144^ ^]^ Under high‐resolution TEM (HRTEM), the fast Fourier transform (FFT) pattern demonstrates distinctive images where the (110) spots were only distinguished in the O‐LNMO.

Theoretical analysis has thoroughly elucidated the crystal structure difference and the distinct charge‐discharge curves between the O‐LNMO and D‐LNMO. However, accurately identifying and classifying LNMO samples between them as either type or determining how much of each portion within the sample remains a significant challenge. Furthermore, the LNMO samples normally demonstrate in‐between characteristics when measured under characterization techniques such as NMR, FTIR, and Raman.^[^ ^145^, ^146^, ^147^, ^148^ ^]^ The co‐existence of the ordered and disordered structure was reported by Kim et al.^[^ ^149^ ^]^ and Zhu et al.^[^ ^150^ ^]^ separately. In other words, as described by Zhu et al.,^[^ ^54^ ^]^ the ideally ordered LNMOs possess consistent structures between the global and local structures, while the disordered structure exhibits mysterious local structures with a long‐range (>1000 Å) cation arrangement.

Several studies have indicated that the ordered and disordered LNMO are derived from different synthesis conditions, especially annealing temperatures. When annealed under a relatively low temperature of around 700 °C, the enthalpy effect delivers an ordered structure.^[^ ^147^, ^151^ ^]^ The high‐temperature sintering (>730 °C), followed by a fast quenching or cooling process, forms kinetically favored highly disordered LNMO due to the entropy effect.^[^ ^145^, ^152^ ^]^ However, for the disordered LNMO, the high‐temperature synthesis process not only accelerates the oxygen release (LiNi_0.5_Mn_1.5_O_4‐δ_ or LiNi^2+^_0.5_Mn^4+^_1.5‐2δ_Mn^3+^_2δ_O_4‐δ_)^[^ ^144^, ^151^, ^153^ ^]^ and leads to partial irreversible rock‐salt phase transformation when the calcination temperature is beyond 750 °C, but also induces the transformation of Mn^4+^ to Mn^3+^.^[^ ^141^ ^]^ The redox reactions at 4.0 V provide concrete evidence of the existence of Mn^3+^ in the LNMO materials.^[^ ^152^ ^]^

Three controversial explanations were adopted for structural defects after oxygen release during the high‐temperature (>700 °C) synthesizing process:

1) An oxygen‐deficient spinel structure LiNi_0.5_Mn_1.5_O_4‐δ,_ partially decomposed into a rock‐salt phase, which possesses lithium migration capability.^[^ ^144^, ^153^, ^154^, ^155^ ^]^
2) The formation of oxygen vacancies (V_O_) reduced the adjacent Mn^4+^ to Mn^3+^ and lowered the lattice energy.^[^ ^156^, ^157^, ^158^ ^]^
3) Extra Li, Ni, or Mn occupied the vacant octahedral sites and were theoretically calculated with lower defect formation energies, which was also recognized as a metal‐excess model.^[^ ^154^ ^]^

Very recently, Zhang et al.^[^ ^159^ ^]^ reported a new strategy that could potentially realize an approximately 1.5 times increment of the energy density of LNMO‐based LIBs from 600 to 900 Wh kg^−1^ by extending the low cut‐off voltage of LNMO to 2.0 V and employing an extra prolonged plateau length at 2.7 V. From this work, it was discerned that D‐LNMO possessed a longer 2.7 V plateau (capacity of 100 mAh g^−1^) than O‐LNMO (capacity of 60 mAh g^−1^). It was intriguing to remark that such observation exhibited an opposite result to the previous studies,^[^ ^160^, ^161^, ^162^ ^]^ that the ordered structure possessed a longer 2.7 V plateau than the disordered structure. Thus, it was depicted that the ordering of Ni/Mn had a negligible influence on determining the 2.7 V capacity. The authors probed the root causes and emphasized that the occupation of Ni‐ions at the 16c octahedral site was the structurally critical determining factor of the mechanisms at the 2.7 V plateau. A shorter 2.7 V plateau originating from the 16c octahedral site occupied Ni^2+^‐ions with the much stronger electrostatic repulsion pushing the neighboring 8a tetrahedral sites Li‐ion to the empty 16c site, resulting in an advanced phase transition. Consequently, the accumulation of Li‐ions occupation in the 16c sites up to 40% induced the phase transformation from cubic to tetrahedral. Conversely, if the 16c octahedral site was originally empty without Ni‐ions occupations, more Li‐ions would be capable of insertions, due to the relatively small repulsion force, until 70% occupation is reached (at 2.7 V plateau) and followed by the abovementioned phase transition. Eventually, the D‐LNMO could deliver energy density of 900 Wh kg^−1^, whereas ≈800 Wh kg^−1^ was achieved by O‐LNMO with 5.4% Ni at the 16c site.

#### 2.2.2 Electrochemical Behavior

In LNMO, the 3D diffusion channel comprises tetrahedral 8a sites, 48f sites, as well as octahedral 16c sites, which are highly favorable for Li‐ion transportation. During electrochemical cycling, the TM redox couples within LNMO spinel structures undergo redox reactions at different potentials. For instance, at high voltage, the Ni‐ion redox couple undergoes dominating redox reactions at 4.8 V and 4.6 V (vs Li/Li^+^) with a small gap for Ni^4+^/Ni^3+^ and Ni^3+^/Ni^2+^, respectively. While at approximately 4.0 V, the Mn^4+^/Mn^3+^ redox couple proceeds with redox reactions.^[^ ^163^ ^]^

For all ASSLIBs, a fast Li‐ion pathway is always desired to achieve exceptionally high energy density. In general, for a cathode material with a close‐packed crystalline structure, a face‐centered‐cubic anion arrangement is the most favorable. The facile Li‐ion transportation of the ordered LNMO spinel material is attributed to the face‐sharing tetrahedral 8a sites and octahedral 12d sites. Meanwhile, for the disordered LNMO spinel structure, Ni and Mn are distributed into the 16c sites. With the help of a high Li/TM ratio, the fast Li‐ion transport pathways allow fast galvanostatic charge‐discharge rates.^[^ ^34^ ^]^ Therefore, the LNMO cathode material with a face‐centered disordered structure ($\mathrm{Fd}\bar{3}m$) exhibits a better rate capability and electrochemical performance than those of the cubic ordered structure (P4_3_32).^[^ ^164^, ^165^ ^]^

During the charging process, the Li‐ions, which are extracted from the cathode, migrate from the 8a sites through the 8a‐16c‐8a channel to the anode. At the same time, the TMs in the cathode undergo oxidation reactions, such as Ni^2+^ is oxidized to Ni^4+^, a small portion of Mn^3+^ is oxidized to Mn^4+^, while Mn^4+^ remains unreacted. Once all the lithium ions are completely extracted from LNMO, a stable spinel structure‐Ni_0.5_Mn_1.5_O_4_ is eventually formed.^[^ ^166^ ^]^

From the perspective of the parental lattice changes in the lithium insertion and extraction process, the structural changes of the lattice reflect different reactions. For instance, in a single‐phase reaction, the parental lattice only experiences the size changes, but without a phase transformation. Meanwhile, the reaction with the destruction of the original lattice and the formation of the new structure is recognized as a two‐phase reaction. Typically, the two‐phase reaction involves nucleation, growth, and the movement of grain boundaries, which creates a sluggishness of Li‐ion transport.

O‐Li_x_Ni_0.5_Mn_1.5_O_4_ experiences 2 first‐order phase transitions during the whole electrochemical cycling process. From 0 < x < 1, the changes of the lattice indicate the phase evolution between LNMO/L_0.5_NMO and L_0.5_NMO/NMO.^[^ ^167^ ^]^ Within the 0.5 < x < 1 range, the D‐Li_x_Ni_0.5_Mn_1.5_O_4_ undergoes a single first‐order phase Ni^2+^/Ni^3+^ redox reaction. When x < 0.5, the redox couples Ni^3+^/Ni^4+^ are perceived and associated with the D‐Li_x_NMO, gradually shrinking the lattice. Consequently, D‐LNMO suffers a two‐phase reaction forming spinel Li_0.5_Ni_0.5_Mn_1.5_O_4_ and rock‐salt Ni_0.25_Mn_0.75_O_2_.^[^ ^149^, ^168^, ^169^ ^]^ These phase transformations are clearly indicated in the CV and differential capacity graph,^[^ ^170^, ^171^, ^172^ ^]^ and the peaks correspond to the structural changes of the phases. Bhatia et al.^[^ ^173^ ^]^ employed Raman spectroscopy^[^ ^174^ ^]^ to determine the valence changes of the Ni‐ion in the D‐LNMO during the charging and discharging process. During the half delithiation process (0.5 < x < 1) within D‐Li_x_Ni_0.5_Mn_1.5_O_4_, at the 4.7 V plateau, the Ni^2+^ decreased from 97% to 15%, and accordingly, the Ni^3+^ increased from 3% to 85% without the formation of Ni^4+^. In the following second‐half delithiation process (0 < x < 0.5), all the Ni^3+^ was gradually oxidized to Ni^4+^ at the 4.75 V voltage plateau. During the electrochemical redox reactions, the changes in the Ni valence (Ni^2+^/Ni^3+^/Ni^4+^), which were reflected as the Ni‐O bond strength, could be precisely observed by Raman spectra.

It is important to realize that the ordered and disordered LNMO exhibit different electrochemical behaviors during the charging and discharging cycles.^[^ ^160^, ^169^, ^175^, ^176^, ^177^, ^178^ ^]^ For a typical O‐LNMO, no obvious plateau is observed at 4.0 V, suggesting a minimal amount of Mn^3+^ in O‐LNMO. The single long flat plateau at 4.7 V with a voltage gap of around 20 mV indicates the redox reaction of Ni^2+^/Ni^4+^ without the intermediate of Ni^3+^. Meanwhile, for a typical D‐LNMO cathode, the first conspicuous plateau at 4.0 V originates from the Mn^3+^/Mn^4+^ redox reactions. The following two long plateaus at 4.6 V and 4.8 V correspond to the redox reactions of Ni^2+^/Ni^3+^ and Ni^3+^/Ni^4+^, respectively. Typically, an approximate voltage gap of 60 mV emerges between the two plateaus.

The crystal structure of LNMO may vary between ordered and disordered due to the different synthesis conditions, which are affected by oxygen content and particle morphology. Attributed to the distinct structures, the D‐LNMO delivered better electrochemical performance than O‐LNMO, especially at higher (dis)charge rates.^[^ ^147^, ^151^, ^179^ ^]^ For the reason of containing a certain amount of disordered phase and Mn^3+^, the former possesses higher Li‐ion diffusion and about 2 orders of magnitude higher electronic conductivity than the latter. Brandell's group^[^ ^180^ ^]^ prepared O‐LNMO and D‐LNMO samples with the same properties while maintaining the different degrees of cation orders to exclusively compare and analyze the electrochemical performance of the ordered and disordered LNMOs without being perturbed by other factors. In the initial cycle, the D‐LNMO and O‐LNMO demonstrated a similar discharge capacity of 110–120 mAh g^−1^. In the following long cycling tests, the D‐LNMO exhibited excellent capacity retention, while the O‐LNMO showed gradual capacity decay. Zhang's group fundamentally investigated the effectiveness of the disordered phase and the Mn^3+^ contents through in‐situ XRD to gain a comprehensive understanding of how much disordered phases and Mn^3+^ content in D‐LNMO is optimal in delivering favorable electrochemical performance.^[^ ^179^ ^]^ The content of the disordered phase was controlled by the cooling rates after synthesis. A faster cooling rate resulted in higher disordered phase concentrations, due to the oxygen deficiency. Moreover, it was observed that during the electrochemical cycling process, a relatively higher Mn^3+^ content (10.8%) resulted in merging the first 2‐phase transition into a solid solution reaction, which was also highly favored for lithium transport and promoted enhanced Li‐ion diffusivity, leading to improved rate capabilities. The samples with optimized disordered phase (cooling rate at 5 °C min^−1^) delivered a capacity of 96 mAh g^−1^ at 10 C than samples with a 0.5 °C min^−1^ cooling rate (54 mAh g^−1^). The optimized disordered samples also demonstrated superior long‐cycling stability with a capacity retention of 94.8% after 300 cycles, compared to 78% for the 0.5 °C min^−1^ cooling rate samples. Therefore, an appropriate amount of disordered phase/Mn^3+^ in D‐LNMO leads to superior electrochemical performance.

#### 2.2.3 Structural Degradation Mechanism

Structural degradation of the cathodes remains a critical challenge in advancing the practical applications of ASSLIBs. Even though LNMO cathodes are mostly structurally stable during the charge and discharge cycling, their structural degradation at high voltage is still deleterious, even at room temperature.

##### Transition Metal Migration

Transition metal migration is considered the origin of structural degradation. For instance, Kim's group observed faint (002) spots when the cathode delithiation was almost complete (Li_0.04_) at the high state of charge (SOC).^[^ ^144^ ^]^ Huang et al.^[^ ^181^ ^]^ exclusively probed the structural change of LNMO in the first cycle and reported the observation of TM ion migration into tetrahedral lithium sites within 2 nm of the surface, resulting in the formation of a Mn_3_O_4_‐like structure. The STEM‐HAADF and STEM‐EDS images on the subsurface regions revealed that the formation of the rock‐salt structure originated from the migrated TM ions occupying empty octahedral sites. This TM ions' migration behavior significantly contributed to the structural degradation and poor initial Coulombic efficiency. Song et al.^[^ ^155^ ^]^ revealed the surface structural revolution of LNMO when discharged to 1.5 V. From the observation of HRTEM images of discharged LNMO, it was noticed that the distinct crystalline grains indicated the co‐existence of multiple phases.

It is plausible to anticipate that the impedance phases resulting from irreversible structural degradation during the first cycle continue to accumulate over the following cycles, especially over the prolonged cycles. Itoh and Imai investigated the local structure changes of D‐LNMO during the first cycle and the 28th cycle through synchrotron X‐ray analyses.^[^ ^182^ ^]^ The D‐LNMO remained $\mathrm{Fd}\bar{3}m$ throughout full electrochemical cycling. The lattice parameter changes between the first cycle and the 28th cycle indicated structural evolutions were present, including cation mixing, loss of Li‐ions in the cathode after long cycling, (Ni, Mn)‐O_6_ octahedral distortion, Ni and Mn valences increasing, and (Ni, Mn)‐O angle departures. It was also observed that up to 0.6 wt.% of NiO was formed in the cathode, which resulted in the reduction of LNMO and was considered directly related to the degradation of the capacity. Brandell's group^[^ ^180^ ^]^ revealed that the O‐LNMO exhibited delayed Ni–O band oxidation and the increased thickness of the interface after ten cycles, which evidenced the occurrence of side reactions. Additionally, O‐LNMO showed ordering structural degradation along with the phase transformation toward the rock‐salt structure at the subsurface.

##### Li/Ni Disorders

Fu et al.^[^ ^183^ ^]^ discovered that the disorder of Li^+^ and Ni^2+^ was also considered another factor that affects the kinetics of Li‐ion intercalation/de‐intercalation and crystal structural stability. Similar to Mn dissolution, the origin of Li^+^/Ni^2+^ disorder was the different energies between Ni 3d and O 2p orbitals, subsequently resulting in weak Ni^2+^ and O orbital interactions.^[^ ^184^, ^185^ ^]^ Weak orbital interactions resulted in Ni^2+^ ions at the 16d position being unstable and migrating to the 8a position to maintain crystal structure stability. Consequently, the Ni^2+^ migrations hindered the Li‐ion 8a to 16d diffusion pathway.

##### High Mn^3+^ Concentration Induced Jahn–Teller Distortion

The two‐phase separation at the high‐voltage state affected the kinetics of Li‐ion transport and the distortion of the crystal structures.^[^ ^186^, ^187^ ^]^ Therefore, exploiting the optimized Mn^3+^ content in LNMO could facilitate the enhancement of composition‐structure‐properties correlations because it was considered that the increased concentration of Mn^3+^ within D‐LNMO was more prone to facilitate solid‐state transition than the two‐phase transition. Hence, the lattice strain caused by the two‐phase transition could be sufficiently suppressed. However, the higher contents of Mn^3+^ also promoted the Jahn–Teller effect, resulting in lattice distortion and structural instability. In addition, the local and microscopic distortion of Ni^3+^ was also affected by Jahn–Teller distortion since the covalency bond between TM and O subsequently contributed to the dynamic lattice instability. Therefore, the ratio of the ordered and disordered phases plays an imperative role in determining the electrochemical performance of the LNMO‐based ASSLIBs. Ouyang et al.^[^ ^186^ ^]^ developed a multi‐faceted polyhedral structure of LNMO in contrast to the conventional single‐crystal particle octahedral with 1–2 sets of crystal facet structure. The optimized single‐crystal secondary particles, which were produced from the multifaceted primary particles, were confirmed to possess 55% ordered phase and 45% disordered phase. The improvements were attributed to the smaller primary particle sizes with reduced Li‐ion diffusion path and transportation variability between crystal faces, promoting an efficient cycling process and suppressing two‐phase separation. In other words, by controlling the synthesis conditions and parameters, optimized composition‐structure‐properties correlations could be achieved with superior LNMO performance.

##### Irreversible Rock‐Salt Phase Transformations

Similar to the challenge that was associated with the rock‐salt phase transformation during the LMO annealing process, different LNMO synthesis conditions led to deviations and discrepancies from the theoretical stoichiometry, and observations of excess Mn were widely reported.^[^ ^144^, ^145^, ^164^ ^]^ However, previous studies suggested that this phenomenon was caused by the oxygen vacancies and low oxygen‐metal ratio that resulted in structural disorder and formation of the rock‐salt phase.^[^ ^188^, ^189^ ^]^ Some studies manifested the Mn^3+^ observation from the sample with full oxygen occupancy, indicating the non‐direct relationship between oxygen loss and Mn reduction.^[^ ^190^ ^]^ However, there still exists a complicated relationship between structure‐composition‐properties. Cabana et al.^[^ ^191^ ^]^ first reported, under specific synthesis conditions, rather than oxygen vacancies, an excess amount of Mn resulted in the formation of Mn^3+^ and hence, a secondary rock‐salt phase due to charge compensation. This rock‐salt phase encompassed an over‐stoichiometric amount of Mn (ca. 2:1 rather than 3:1) and originated from the extrusion of Ni out of the spinel structure. Moreover, from the insights of Ni/Mn arrangements in LiNi_0.5_Mn_1.5_O_4_ ordering and disordering schemes, the ordered scheme did not necessarily possess perfect Ni and Mn ordering; meanwhile, the disordered scheme also showed some degree of preferred Ni/Mn distributions.

Even though several works have experimentally reaffirmed the explanation discussed above, it is still unclear whether the electrochemical performance is affected by one factor or a combination.^[^ ^192^, ^193^ ^]^ A holistic understanding of the LNMO structural degradation mechanism is paramount to achieving an optimized electrochemical performance in ASSLIBs.

##### Volume Changes

During the ASSLIBs cycling process, the volume change of the cathode and the associated formation of voids at the interphases are another challenge that hinders the Li‐ion transport between the LMO/LNMO cathodes and SSEs. The cubic‐cubic two‐phase transformation, especially at high voltage, induces significant lattice volume change. Such volume change leads to severe lattice strain, sluggish Li‐ion transport, structural distortion, and cracking, which was also suggested to contribute to the rapid capacity decay of the LMO/LNMO‐based LIBs.^[^ ^164^, ^167^, ^194^ ^]^

In liquid‐state batteries, benefiting from the fluidity of the electrolytes, Li‐ions can maintain excellent transportation within the batteries regardless of the cathode volume change. Within ASSLIBs, any geometrical change at the interfaces (cathode/CEI, CEI/electrolyte) creates insulating voids that prevent both Li‐ion and electrons from diffusing through, thus forming a steadily cumulating interfacial resistance.^[^ ^195^, ^196^ ^]^ For instance, within the voltage range of 3.5 V–5.0 V, the LNMO cathodes undergo a solid‐solution reaction with Ni^2+^/Ni^3+^ redox reactions and form LixNi_0.5_Mn_1.5_O_4_ and a two‐phase reaction during Ni^3+^/Ni^4+^ redox to Ni_0.25_Mn_0.75_O_2_ during electrochemical cycling. Both the solid‐solution reaction and two‐phase reaction are associated with a lattice change of 3 vol.% and subsequently result in a total volume shrinkage of 5.0–6.0 vol.%.^[^ ^167^, ^168^ ^]^ Similarly, at the voltage of 3.96 V and 4.07 V vs Li/Li^+^, spinel LMO cathodes experience two cubic phase transitions. As a result, it is observed that the volume shrinks as much as 6.8 vol.% and the structure changes into λ‐Mn_2_O_4_.^[^ ^197^ ^]^ As a consequence, the CEI on the CAM side could experience interfacial delamination or cracks. This phenomenon is not severe when paired with polymer SSEs due to their excellent flexibility. It is more pronounced to observe the loss of contact between the CAM and the rigid inorganic SSEs. As a result, this process will lead to severe mechanical failure and result in high interfacial impedance and low‐capacity retention.^[^ ^195^, ^196^ ^]^

**Figure 4:**
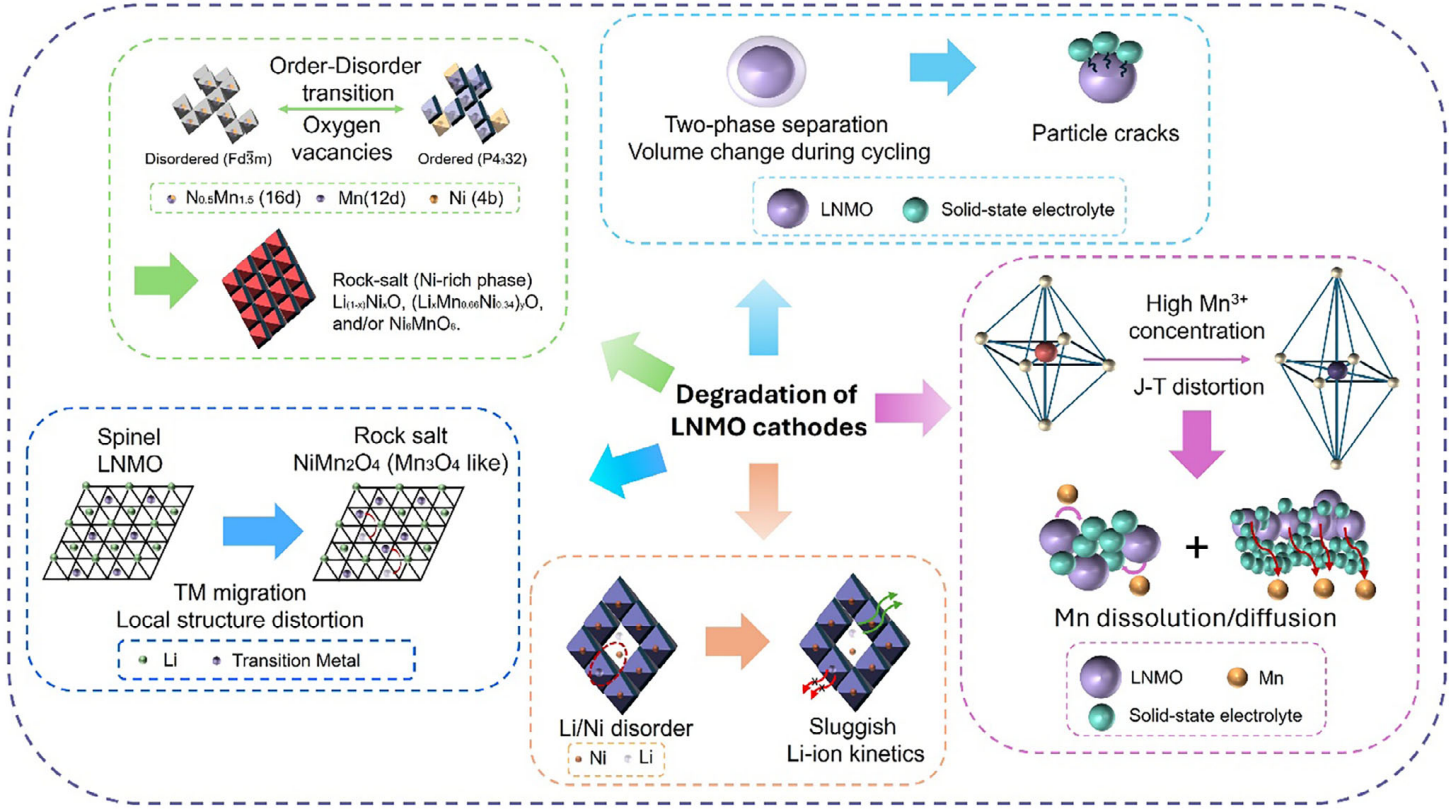
Degradation mechanism of LNMO cathodes.

The degradation mechanism of LNMO cathodes is summarized as shown in **Figure** **4**.

## 3 Mn‐Based Spinel Cathode in All‐Solid‐State Batteries

### 3.1 Polymer Solid‐State Electrolytes

#### 3.1.1 Introduction of Polymer Solid‐State Electrolytes

Polymer solid‐state electrolytes were first introduced by Fenton's group as early as 1973.^[^ ^198^ ^]^ The conductivity of alkali metal salts, lithium iodide (LiI), dissolved in poly(ethylene oxide) (PEO), substantiated the feasibility of deliberating polymer (PEO) as electrolytes. Over decades of probing, development, and enhancement in polymer solid‐state electrolytes, the applications of polymer solid‐state electrolytes in ASSLIBs have become increasingly widespread.

In ASSLIBs, it is ubiquitous that a stable, robust, and conformal interface contact between the electrolytes and cathodes plays an imperative role in determining the rates of Li‐ion transport and is always highly demanded. In contrast to most rigid inorganic SSEs, polymer solid‐state electrolytes possess a unique and intrinsic soft and flexible nature, which is drastically favorable in constructing tight interfacial contact.^[^ ^199^, ^200^, ^201^, ^202^ ^]^

In general, polymer solid‐state electrolytes comprise polymers as the solid matrix host and alkali metal salts as solutes. Therefore, both dielectric constants and ionic conductivity fulfill critical functions in determining efficient Li‐ion transport. The higher dielectric constants and donor numbers of the matrix host lead to stronger lithium‐salt solvate capabilities, improved dissociated Li‐ion concentrations, and an increasing lithium transference number. Thus, the augmented ionic conductivity of the solid polymer electrolyte contributes to facilitating facile and efficient Li‐ions migration pathways within ASSLIBs.^[^ ^203^, ^204^, ^205^, ^206^ ^]^ In the application of polymer solid‐state electrolytes, there is a constant drive to pursue higher dielectric constants and ionic conductivities.

Depending on the solid matrix host, polymer solid‐state electrolytes are categorized into different types. Due to the structure variations, each type of solid polymer electrolyte possesses inherent chemical, electrochemical, and mechanical properties. Beyond the typical PEO‐based polymer solid‐state electrolytes, other polymer solid‐state electrolytes that are based on PAN, PVDF, and PMMA are also scrutinized.

**Figure 5:**
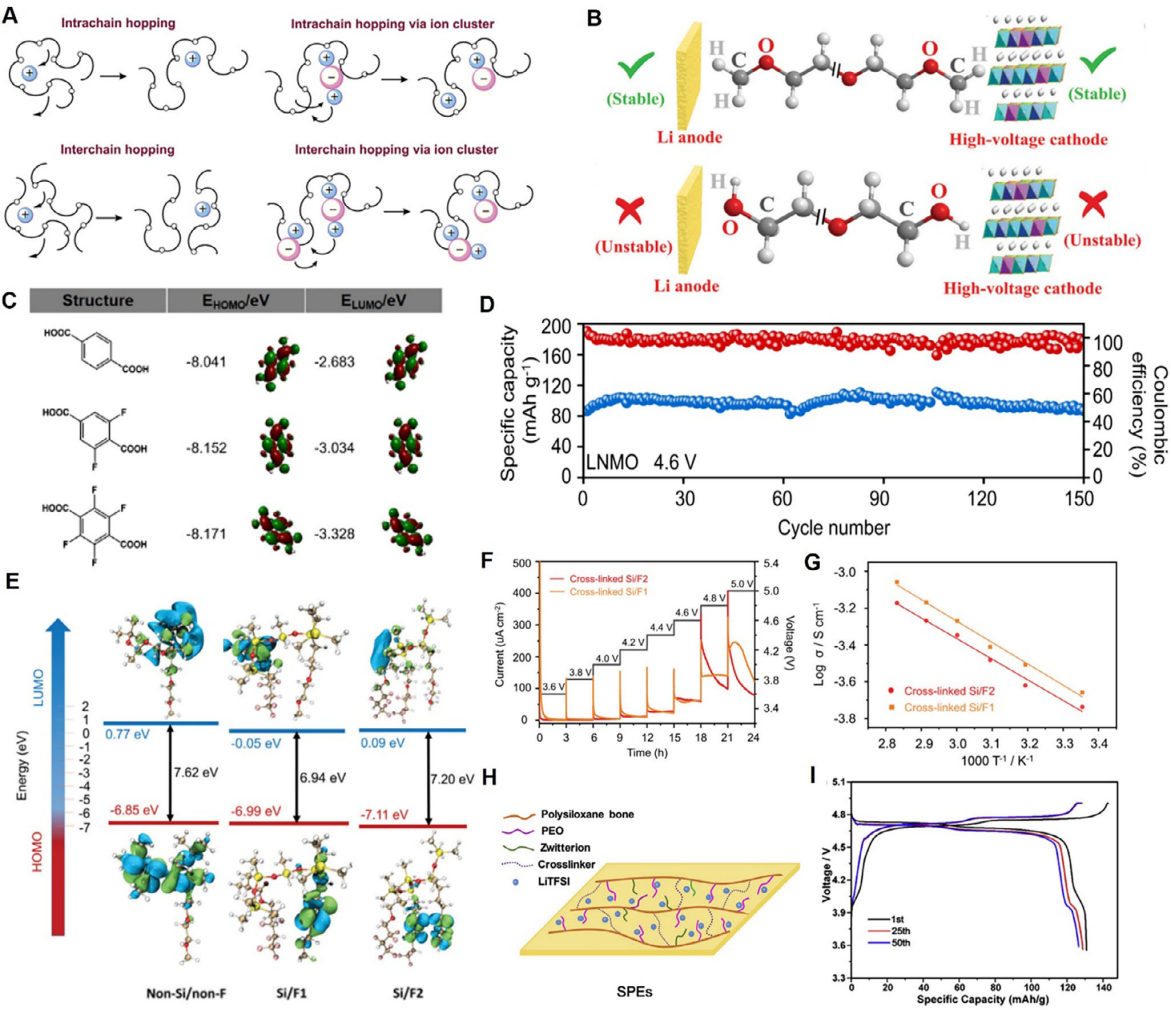
A) Schematic of intrachain and interchain ion hopping mechanism in PEO. Reproduced with permission.^[^ ^208^ ^]^ Copyright 2015, The Royal Society of Chemistry. B) Schematic illustration of terminal group effect in the electrochemical stability window of PEGDME (top) and PEG (bottom). Reproduced with permission.^[^ ^233^ ^]^ Copyright 2020, The Royal Society of Chemistry. C) DFT calculation of fluoride modifications and corresponding HOMO and LUMO energy levels. D) Cycling electrochemical performance of LNMO//50% 4F‐MOF/PEO//Li ASSLIBs to 4.6 V at 0.1C and 60 °C. Reproduced with permission.^[^ ^249^ ^]^ Copyright 2024, Wiley. E) Calculated HOMO and LUMO energy levels of polymer precursors (left) and polymer with silicone and fluorine modifications (right). F) Potentiostatic hold tests for cross‐linked Si/F2 and Si/F1 from 3.6 V to 5.0 V at 60 °C. G) Ionic conductivity of cross‐linked Si/F2 and Si/F1 in the range of temperature 25 °C to 80 °C. Reproduced with permission.^[^ ^251^ ^]^ Copyright 2024, Wiley. H) Schematic of zwitterion and PEO synergistic polysiloxane‐based solid polymer electrolyte. I) Typical charge‐discharge profiles of LNMO cathodes and cross‐linked polysiloxane‐based polymer solid‐state electrolytes between 3.5 V and 4.9 V at 30 °C. Reproduced with permission.^[^ ^226^ ^]^ Copyright 2018, Elsevier.

PEO‐based electrolytes, regarded as the benchmark among polymer solid‐state electrolytes, have the most extensive applications in ASSLIBs. The PEO‐based polymer solid‐state electrolytes exhibit outstanding lithium salt solubility due to the high Li^+^ donor number, which contributes to ionic conductivity by increasing the concentration and the mobility of the charge carriers.^[^ ^207^ ^]^ A schematic of the intrachain and interchain ion transport mechanism within PEO polymer solid‐state electrolytes is illustrated in **Figure** **5**A.^[^ ^208^ ^]^ Notably, due to the unique ion transport mechanism and crystallinity, PEO‐based polymer solid‐state electrolytes exhibit ionic conductivity as low as 10^−5^ S cm^−1^ at room temperature, which dramatically restricts their potential for harnessing in ASSLIBs.^[^ ^209^, ^210^, ^211^ ^]^

PAN‐based polymer solid‐state electrolytes possess unique “─C≡N” terminals within the polymer matrix structure. This polar terminal could facilitate expeditious Li‐ion pathways during lithium migrations and high compatibility with large amounts of plasticizers. Therefore, the PAN‐based polymer solid‐state electrolytes demonstrate a relatively similar room‐temperature ionic conductivity of 10^−6^ S cm^−1^ as PEO‐based polymer solid‐state electrolytes. The low HOMO energy levels provide excellent antioxidation capability, which could attain upper end of the electrochemical stability window higher than 5.0 V vs Li/Li^+^. The formed chelation between the transition metal ions (Mn for LMO and Mn/Ni for LNMO) and the terminal functioned in capacity retention and suppressed the unfavored TM dissolution.^[^ ^173^, ^212^, ^213^, ^214^, ^215^ ^]^ Additionally, the rigid bonds between the C and N atoms act as a double‐edged sword. While the inherent resilient strength of the bond is beneficial in maintaining a stable structure, it also leads to difficulties in structural rotations, subsequently resulting in deteriorating brittleness.^[^ ^216^ ^]^

PVDF‐based polymer solid‐state electrolytes are also remarkable because of their outstanding electrochemical antioxidation stability, great thermal stability, and excellent mechanical properties. The functional group “─CF” in PVDF undertakes vital tasks in determining the electrochemical properties of PVDF‐based polymer solid‐state electrolytes. For example, the intrinsic high polarization and dielectric constant properties could promote lithium salt dissociation.^[^ ^217^, ^218^, ^219^, ^220^ ^]^ However, similar to the PEO‐based polymer solid‐state electrolytes, the high crystallinity of the PVDF results in limited ionic conductivity.^[^ ^221^ ^]^

Due to the amorphous crystallinity of the PMMA, the PMMA‐based polymer solid‐state electrolytes usually possess a relatively higher room‐temperature ionic conductivity of 10^−5^ S cm^−1^ than PEO‐based polymer solid‐state electrolytes.^[^ ^222^ ^]^ The ester group within PMMA enables the capability of incorporation with higher amounts of plasticizers. However, even possessing the advantage of lower manufacturing costs, the drawback of the amorphous structure and weak mechanical properties strictly rendered the practical applications of PMMA‐based polymer solid‐state electrolytes.

In addition to the extensively culminated polymer solid‐state electrolytes mentioned above, several other host matrix‐based electrolytes, including plastic crystal‐based electrolytes,^[^ ^223^ ^]^ polycarbonate‐based,^[^ ^224^ ^]^ polyester‐based,^[^ ^225^ ^]^ and polysiloxane‐based^[^ ^226^ ^]^ polymer solid‐state electrolytes have also been reported for coupling with Mn‐based spinel cathodes in ASSLIBs.

#### 3.1.2 Challenges in Coupling with Mn‐Based Spinel Cathodes

While various types of polymer solid‐state electrolytes exhibited diversified and unique properties, some pragmatic challenges associated with polymer solid‐state electrolytes when pairing with Mn‐based spinel cathodes hinder the commercialization of solid polymer electrolyte‐based ASSLIBs. Chief among these challenges is 1) incompatibility with cathodes at high working voltages and 2) unsatisfactory ionic conductivity at room temperature.

1) The electrochemical incompatibility between the cathodes and the polymer electrolytes is a severe obstacle that limits the applications of polymer solid‐state electrolytes in ASSLIBs. For instance, the PEO‐based polymer solid‐state electrolytes possess a narrow electrochemical stability window (<3.9 V vs Li/Li^+^).^[^ ^227^ ^]^ When coupled with Mn‐based cathodes (LMO and LNMO), the mismatched electrochemical stability window leads to the oxidation of the electrolytes. Gao et al.^[^ ^228^ ^]^ reported the oxidative decomposition of PEO under high voltage resulted in gas generation (CO_2_ and O_2_), which was also experimentally confirmed by the in‐situ DEMS measurement. As a result, the capacity degradation, which was ascribed to the electrolyte decomposition, ultimately caused performance failure in long‐term cycling.
2) Another significant challenge of polymer solid‐state electrolytes is the low room‐temperature ionic conductivity, which significantly hinders Li‐ion transport at the cathode/SSE interface. For example, within PEO‐based polymer solid‐state electrolytes, it is indispensable to be aware that the crystallinity of the PEO matrix critically determines the ionic conductivity of the electrolytes. The amorphous structure favors ionic conduction, while the crystalline structure limits the motions of ions.^[^ ^229^ ^]^ Unfortunately, since the solid polymer electrolyte chains normally crystallize under 65 °C, consequently, most of the polymer solid‐state electrolytes exhibit much lower ionic conductivity (10^−8^–10^−7^ S cm^−1^) than the expected>10^−3^ S cm^−1^ level at room temperature.^[^ ^210^, ^230^, ^231^ ^]^ On the other hand, with a frequently employed strategy of elevating the temperature of operation to around 60 °C, the ionic conductivity is improved, but at the cost of compromising the mechanical strength.^[^ ^232^ ^]^

Therefore, to meet a satisfactory electrochemical performance when coupled with Mn‐based spinel cathodes, the great challenge for the polymer solid‐state electrolytes is to simultaneously meet both criteria of high ionic conductivity and great electrochemical stability. Accordingly, numerous endeavors have been conducted focusing on improving the chemical, physical, and electrochemical properties of polymer solid‐state electrolytes, thus enhancing the overall electrochemical performance of ASSLIBs.

#### 3.1.3 Strategies to Address the Challenges

##### Expanding the Electrochemical Stability Window

Generally, PEO comprises a (─C─O─C─) main chain and a hydroxide group (─OH) as the terminal. To enable the potential of pairing PEO‐based polymer solid‐state electrolytes with the high‐voltage cathodes, it is imperative to identify the fundamental cause of the incompatibility, i.e., whether the main chain or the hydroxide group is the limiting factor. To scrutinize the origin of the incompatibility, Sun et al.^[^ ^233^ ^]^ studied two poly(ethylene glycol)‐based polymers that possessed the same main chain but different terminals, i.e., one with (─OH), the other one with (─OCH_3_) for comparison, as shown in Figure 5B. The electrochemical analyses revealed that the polymer with the terminal (─OCH_3_) demonstrated an electrochemical stability window up to 4.3 V with stable charge–discharge cycling, in contrast to the polymer electrolyte with the terminal (─OH) of 4.05 V. These results substantiated the (─OH) terminal was the electrochemical stability window determining factor not the (─C─O─C─) main chain. Therefore, tuning the structure by replacing the limiting terminal within the polymer solid‐state electrolytes could fundamentally extend the electrochemical stability window to enable the potential of coupling with high‐voltage Mn‐based cathodes in ASSLIBs

Alternatively, tuning the microstructure by incorporating the terminals from different polymer electrolytes could attain a wider electrochemical potential window. Hou et al.^[^ ^234^ ^]^ expanded the electrochemical stability window of the PAN‐based polymer electrolyte by interpenetrating the PAN and cross‐linking with PEO. The modified polymer electrolyte manifested a remarkably high oxidation reaction stability of 5.5 V, attributed to the stabilization provided by the inherent chemical structure of the nitrile group (─C≡N). Moreover, with this strategy, the crystallinity of the PEO was profoundly decreased, and accordingly, the room‐temperature ionic conductivity of the polymer electrolyte was increased to 3.0 × 10^−3^ S cm^−1^. Pan's group^[^ ^235^ ^]^ reported a 3D‐polymer electrolyte by copolymerizing poly (lactic acid)‐poly (ethylene glycol)‐poly (lactic acid), followed by mixing with polyacrylonitrile. This composite electrolyte achieved an expanded electrochemical stability window up to 5.11 V. The terminal groups (─C═O, ─C─O─C─, and ─C≡N) also functioned in dissociating the LiTFSI salt, resulting in a high ionic conductivity of 0.84 mS cm^−1^. A block copolymer elastomeric electrolyte, which was prepared by PMMA grafted to polyisoprene (MG_30_), was reported by Zhang et al.^[^ ^236^ ^]^ The LSV test indicated the polymer electrolyte possessed an electrochemical stability window of up to 5.08 V, which was also consistent with the previously reported 5.2 V from the DFT computational calculation^[^ ^237^ ^]^ and 5.14 V from LSV.^[^ ^238^ ^]^ The oxidation stability at high voltage originated from the high loading of LiTFSI. Tan et al.^[^ ^239^ ^]^ introduced a copolymer P(MMA‐AN)‐based polymer electrolyte that demonstrated an expanded electrochemical stability window up to 4.5 V and was confirmed by LSV. The authors also incorporated PVAc (polyvinyl acetate) to develop a P(MMA‐AN‐Vac)‐based copolymer electrolyte, which achieved an electrochemical stability window of 5.6 V vs Li/Li^+^. Exploiting the intrinsic advantages of excellent electrochemical and thermal stability of AN, flexibility of MMA, and mechanical stability of Vac, the new polymer electrolyte manifested excellent physical and chemical properties. In addition, the low crystallinity of the P(MMA‐AN‐Vac)‐based polymer electrolyte further facilitated a high room‐temperature ionic conductivity of 3.48 × 10^−3^ S cm^−1^.

In the modifications of polymer solid‐state electrolytes, metal‐organic framework (MOF) can provide a favorable, efficient, facile, and free ion migration pathway and hence elevate the ionic conductivity.^[^ ^240^, ^241^, ^242^, ^243^ ^]^ This improvement is attributed to the intrinsic porous structure of MOFs along with their remarkable thermal and chemical stability. However, MOF‐based polymer solid‐state electrolytes still face the drawback of the constrained electrochemical potential window due to the energy gap between HOMO and LUMO.^[^ ^244^, ^245^ ^]^ As a result, when coupled with LNMO cathodes, the electrochemical incompatibility hinders their applications in ASSLIBs. On the other hand, previous research revealed that fluorination in molecules was shown to enable expanding the energy gap between HOMO and LUMO and subsequently enlarging the electrochemical stability window.^[^ ^246^, ^247^, ^248^ ^]^ Inspired by the aforementioned studies, Lin et al.^[^ ^249^ ^]^ utilized a novel fluorinated MOF (4F‐MOF) strategy by incorporating the MOF and fluorination to tune the energy level of the PEO‐based composite electrolyte, especially HOMO, to realize the capability of coupling with the high‐voltage cathode LNMO while maintaining an excellent ionic conductivity. The optimized 50% 4F‐MOF/PEO‐based solid polymer electrolyte manifested superior antioxidation capability and anodic voltage of 5.1 V, in contrast to 4.2 V of 50% MOF/PEO‐based polymer electrolyte without fluorination, as shown in Figure 5C. The assembled ASSLIBs demonstrated a stable charge‐discharge performance up to 4.6 V at 0.1 C and 60 °C, with an initial discharge capacity of 93.5 mAh g^−1^ (Figure 5D). A significant improvement over the ASSLIBs without fluorination modification. After 150 cycles, the formation of LiF was observed on the cathode and electrolyte interface (CEI) layer, which originated from the decomposition of LiTFSI and 4F‐MOF during the cycling. The formed LiF could effectively contribute to stabilizing the CEI and preventing the oxidation of PEO‐based polymer solid‐state electrolytes.^[^ ^250^ ^]^ Very recently, Lin's group successfully developed a method to extend the solid polymer electrolyte's electrochemical window by tuning the polymer's backbone with an in situ hybrid Si/F 3D network.^[^ ^251^ ^]^ The polymers poly(methylhydrosiloxane) (PMHS) and 2,2,3,4,4,4‐hexafluorobutyl methacrylate (HFBMA) were used as main‐chain and fluorinated pendants, respectively, and poly(ethylene glycol)allyl methyl ether (PGAME) was applied to improve salt solubility. The optimized solid polymer electrolyte with increased fluorination degree (Si/F2) expanded the energy levels between HOMO and LUMO to 7.2 eV, as shown in Figure 5E, and the electrochemical window was enlarged to 4.9 V, which was much higher than the fluoropolymer sole of 4.5 V.^[^ ^247^, ^248^, ^249^, ^250^, ^251^, ^252^ ^]^ Through the potentiostatic hold test by holding the charging potential for 3 hours from 3.6 V to 5.0 V, cross‐linked Si/F2 exhibited increased stability at 5.0 V as shown in Figure 5F. The solid polymer electrolyte also exhibited superior ionic conductivity of 1.83 × 10^−4^ S cm^−1^ at ambient temperature (Figure 5G). The ASSLIBs with LNMO as cathode achieved an initial discharge capacity of 144 mAh g^−1^ and 87.5% capacity retention after 30 cycles under 0.5C at 60 °C, which further evidenced the stabilized CEI by hybrid Si/F. Additionally, eutectic electrolyte^[^ ^253^ ^]^ was blended with Si/F‐modified solid polymer electrolyte to further enhance the room‐temperature ionic conductivity. The optimized ionic conductivity was achieved at 1.59 × 10^−3^ S cm^−1^, and the ASSLIBs enabled room temperature operation of the LNMO high voltage cathode battery with an initial capacity of 142 mAh g^−1^.

##### Enhancing Ionic Conductivity

Several approaches have been developed to enhance the ionic conductivity of polymer solid‐state electrolytes, such as adding organic plasticizers,^[^ ^254^ ^]^ preparing with inorganic active fillers,^[^ ^255^ ^]^ and filling non‐ionic materials.^[^ ^256^, ^257^, ^258^ ^]^ However, as mentioned above, adding Li‐salt is the most efficient and economical method to elevate the ionic conductivity of polymer solid‐state electrolytes. It was reported by Mousa et al.^[^ ^259^ ^]^ that by introducing LiI into PEO as a solid electrolyte, the ionic conductivity was greatly improved from 3.50 × 10^−9^ S cm^−1^ (PEO pristine) to 1.67 × 10^−4^ S cm^−1^. Thus, the assembled hybrid BatCap batteries with LMO as cathode and a hybrid anode encompassed with polyaniline (PANI), graphene, and nanostructure‐modified LTO, not only delivered a high initial discharge capacity of 173 mAh g^−1^ at 1 C and 90.5% capacity retention after 1000 cycles, but also a remarkable rate capability up to a high current density of 50 C. This result showed that adding Li‐salt can effectively improve the ionic conductivity of polymer solid‐state electrolytes.

Tuning the structure of the host matrix by incorporating another functioning polymer to benefit from the advantages of both polymers could also profoundly improve the ionic conductivity of the polymer solid‐state electrolytes. In Chen's research, a novel modification of polysiloxane (PS)‐based solid polymer electrolyte was reported, which not only demonstrated an excellent ionic conductivity of 3.39 × 10^−4^ S cm^−1^ at room temperature, but also an expanded electrochemical window to 5.0 V.^[^ ^226^ ^]^ The new polymer solid‐state electrolytes were prepared by grafting a polysiloxane backbone with sulfobetaine zwitterion and PEO, followed by tri(ethylene glycol) divinyl ether crosslinked as shown in Figure 5H. This modification further proved that the functionality of adding zwitterions into the polymer solid‐state electrolytes could enhance the diffusion coefficient of Li‐ions and improve the ionic conductivity,^[^ ^260^ ^]^ and grafting the PEO to the backbones could increase its mobility and flexibility, thus improving the ionic conductivity.^[^ ^261^ ^]^ The ASSLIBs comprised an LNMO cathode, and the novel developed polymer solid‐state electrolytes delivered an initial discharge capacity of 131.5 mAh g^−1^ at 30 °C, almost 90% of the theoretical capacity of 146.7 mAh g^−1^ (Figure 5I). The ASSLIBs could maintain 95.8% capacity retention after 50 charging and discharging cycles.

**Figure 6:**
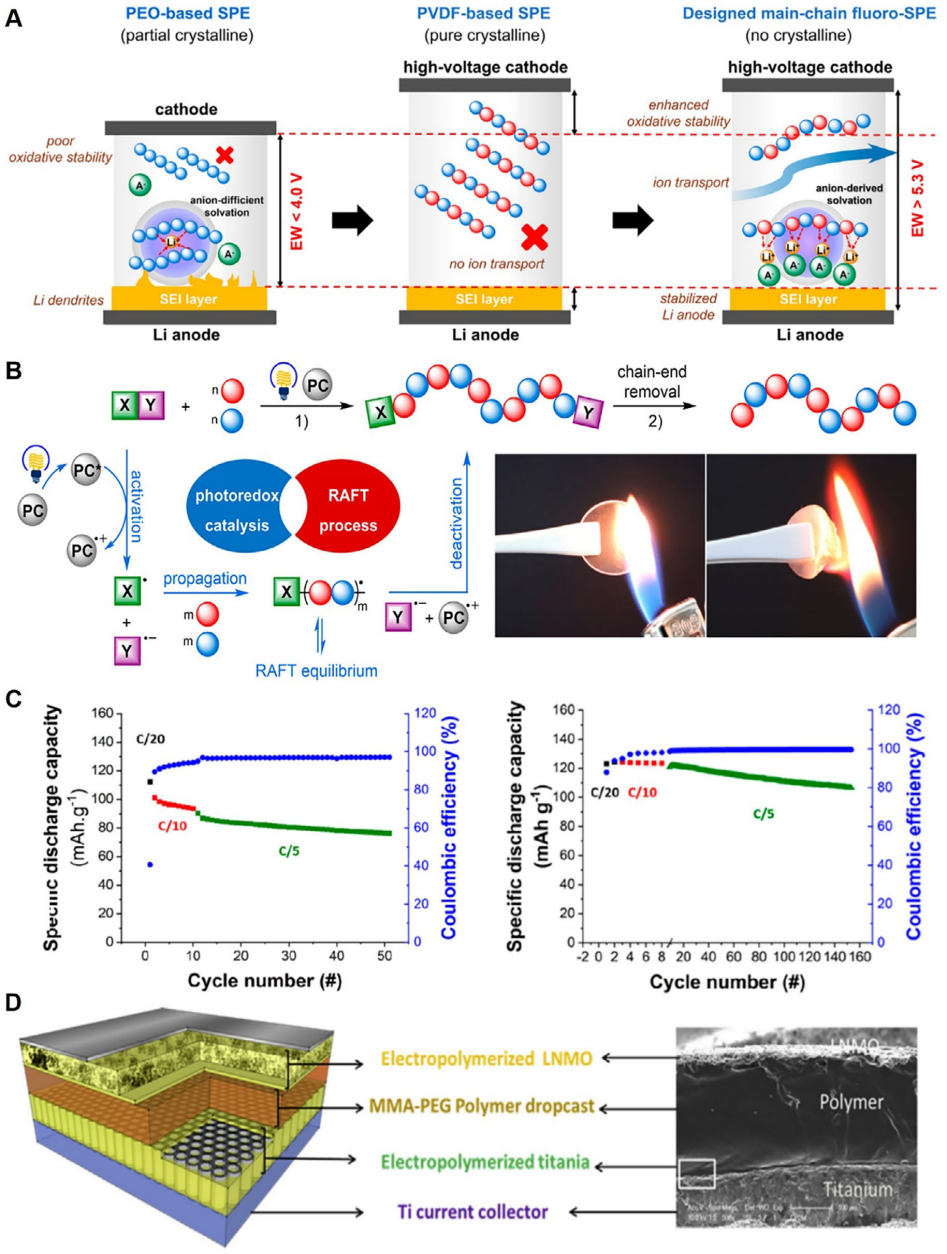
A) Schematic illustration of fluoropolymer electrolytes in stabilizing both cathode and anode interfaces with improved ionic conductivity. B) Schematic of copolymerization synthetic process and a combustion test comparison of MCF‐based solid polymer electrolyte (left) vs PEO‐based (right). Reproduced with permission.^[^ ^252^ ^]^ Copyright 2021, American Chemical Society. C) The cycling discharge electrochemical performance of ASSLIBs consists of LMO‐based cathodes with PVDF‐based solid polymer electrolyte (Left) and LIBs with additional ionogel (PMA and C3mPyrFSI ionic liquid) into LMO cathodes (Right). Reproduced with permission.^[^ ^264^ ^]^ Copyright 2023, American Chemical Society. D) Schematic and the corresponding SEM images of LNMO cathodes and MMA‐PEG‐based polymer solid‐state electrolytes ASSLIBs. Reproduced with permission.^[^ ^280^ ^]^ Copyright 2016, Elsevier.

Mainchain fluoropolymers, such as PTFE, PCTFE, and PVDF, possess a high degree of fluorination and could sufficiently benefit interface stabilization. However, their high crystallinity results in low ionic conductivity.^[^ ^262^, ^263^ ^]^ For example, the challenges of the PEO‐based, PVDF‐based polymer solid‐state electrolytes were demonstrated in **Figure** **6**A. Lin et al.^[^ ^252^ ^]^ developed a novel group of main‐chain fluoro‐based polymer solid‐state electrolytes, as shown in Figure 6B, which exhibited ionic conductivity of 3.5 × 10^−5^ S cm^−1^ at 25 °C and 2.7 × 10^−4^ S cm^−1^ at 85 °C. Notably, this solid fluoropolymer film possessed excellent anti‐flammability that remained non‐flammable during the combustion test, in contrast to the immediately ignited PEO‐based solid polymer film.

Howlett et al.^[^ ^264^ ^]^ developed a triblock copolymer solid‐state electrolyte with a sandwich structure to enhance the ionic conductivity. In this work, the ternary system comprised of polystyrene‐based PILBLOC (polymerized ionic liquid (PIL) block copolymer), LiFSI, and ionic liquid (C_3_mPyrFSI). The addition of LiFSI and ionic liquid was engineered to further improve the ionic conductivity of the PILBLOC.^[^ ^265^, ^266^, ^267^ ^]^ By optimizing the ratio between LiFSI and IL, the PILBLOC electrolyte achieved an improved ionic conductivity of 2.3 × 10^−4^ S cm^−1^ at 50 °C. However, due to the electrochemical instability of PILBLOC at high voltage (4.3 V), a severe irreversible capacity of more than 50 mAh g^−1^ was observed. Alternatively, by adding the same ionogel components as the electrolyte additives into the pores of the LMO cathode, the liquid‐based cathode LIBs delivered stable electrochemical performance with close to 90% capacity retention after 150 cycles and 99.4% Coulombic efficiency, as shown in Figure 6C.

Utilizing advantageous preparation methods to tune the microstructure of polymer electrolytes could also provide excellent functionality in promoting ionic conductivity. It had been reported that utilizing the electrospinning method in preparing the polymer electrolytes could facilitate a fiber structure, which possessed the advantages of controllable porosity and amplified mechanical strength.^[^ ^268^, ^269^, ^270^ ^]^ The high porosity structure could effectively facilitate migration paths for Li‐ions and hence improve ionic conductivity. Inspired by the preeminent preparation method, Li et al.^[^ ^271^ ^]^ further developed the PAN/PMMA‐based polymer electrolyte with the electrospinning method. The electrospun‐prepared PAN‐based polymer electrolyte possessed room‐temperature ionic conductivity of 2.3 × 10^−3^ S cm^−1^, much higher than other conventionally prepared PAN‐based polymer electrolytes of 10^−4^ S cm^−1^.^[^ ^272^ ^]^ The electrospun‐prepared fibrous structured PAN/PMMA‐based polymer electrolyte demonstrated an amplified ionic conductivity of the electrolyte of 3.6 mS cm^−1^, which was almost triple that of 1.25 mS cm^−1^ in the previous work.^[^ ^239^, ^273^, ^274^ ^]^ Therefore, tuning the structure on a micro‐scale, especially the porosity of the nevertheless an excellent method to reach the desired ionic conductivity.

In addition to the above‐mentioned strategies, tuning the viscosity of the solid polymer electrolyte enables improving Li‐ion transport properties and ionic conductivity. Theoretically, a high degree of viscosity results in low ionic conductivity due to the intense interactions between Li‐ions and ether‐oxygen atoms within the polymer chains, and thus, is unfavorable in Li‐ion migrations.^[^ ^275^, ^276^, ^277^ ^]^ To gain a comprehensive understanding of the effectiveness of the viscosity on ionic conductivity, Wakihara et al.^[^ ^278^ ^]^ investigated the relationship between the ionic conduction mechanism and the viscosity at the interfaces between the PEGDME‐based electrolytes and the cathodes. It was perceived that introducing additional PEGDME1000 into PEGDME500 raised the viscosity of the PEGDME500‐based electrolyte but decreased the exchanged current density. This result suggested an inversely proportional relationship between the viscosity of the electrolyte and the exchange current density. Furthermore, Raman results revealed that the addition of PEGDME‐1000 into PEGDME‐500 had minimal effect on Li‐ion activities, which further confirmed that the viscosity of the electrolyte was the main rate‐determining factor. In the following work, the same researchers investigated the charge‐transfer reaction rates by evaluating the surface conductivity and charge‐transfer resistance at the interface between the LMO thin film cathode and PEGDME‐based polymer electrolytes.^[^ ^225^ ^]^ The same phenomenon was obtained for this pair of electrodes and electrolytes, indicating that the high viscosity of PEGDME‐based electrolytes decreased the interfacial Li‐ion intercalation and de‐intercalation rates. These results provided insight into the viscosity of the polymer solid‐state electrolytes when rationalizing the design of the solid polymer electrolyte‐based ASSLIBs with Mn‐based spinel cathodes.

##### Engineering the Contact Interface

Due to the intrinsic flexibility of the polymer solid‐state electrolytes, the interfacial contacts between the cathode and the polymer solid‐state electrolytes are not a pronounced concern compared to the other inorganic electrolytes (which will be discussed in the following sections). However, Djenizian et al.^[^ ^279^, ^280^, ^281^ ^]^ revealed that improved interfacial contact can still contribute to promoting the electrochemical performance of polymer‐based ASSLIBs. It is well understood that a higher surface contact area and a shorter ion transportation distance are highly favorable to achieving fast ion transport, thereby improving the overall electrochemical performance of ASSLIBs. For instance, the conformal interface between PMMA‐PEG‐based polymer electrolyte and the electrodes was prepared by the novel electrodeposition method on the surface of the porous LNMO cathodes and TiO_2_ nanotube anodes.^[^ ^280^ ^]^ This conformally constructed interface, as shown in Figure 6D, could effectively aid in enhancing the electrochemical performances of the ASSLIBs. In LNMO‐based ASSLIBs at 0.1C current rate, almost double the initial discharge capacity of 169 mAh g^−1^ was achieved, compared to the conventional top‐down deposition method with the capacity of 90 mAh g^−1^.^[^ ^279^ ^]^

In the following work,^[^ ^281^ ^]^ the research group further advanced the contact interface by enhancing the method of electrodepositing the PMMA‐PEG electrolyte into the porous structure LNMO cathode. The porous structure can remarkably enhance the specific contact area and promote ion and electron migrations.^[^ ^282^ ^]^ Although the interfacial resistance was enlarged with the increasing thickness of the polymers, it was observed that the initial discharge capacity was dramatically increased from 80 mAh g^−1^ to 122 mAh g^−1^. Such improvement further evidenced the effectiveness of the porous structure in expanding the surface contact area. Notably, the rapid capacity decay and large irreversible capacity challenges persisted and led to the deterioration of the overall performance of the ASSLIBs. Therefore, increasing the contact interface is an undoubtedly useful approach to enhancing electrochemical behavior. However, challenges such as electrochemical stability window incompatibility should also be addressed to maintain a stable and prolonged cycling life.

In summary, the abovementioned strategies in this section revealed the feasibility of achieving improved electrochemical performance in the application of Mn‐based spinel cathodes within solid polymer electrolyte‐based ASSLIBs. It should be noted that due to the intrinsic property of possessing a low dielectric constant, it is always challenging to completely dissociate lithium salt in polymer solid‐state electrolytes, for example, PEO‐based polymer solid‐state electrolytes. Consequently, the undissociated lithium salts form undesired clusters and sluggish the ion transportation.^[^ ^283^, ^284^, ^285^ ^]^ Moreover, the advancements in achieving enhanced ionic conductive or expanded electrochemical stability windows are promising in leading polymer solid‐state electrolytes into wider applications. It is still challenging to effectively address the two major difficulties simultaneously. Therefore, further research will be required to mitigate the challenges of polymer solid‐state electrolytes from the novel synthesis or polymerization methods, incorporating advanced‐functioning additives or copolymers, and tuning the structure from the micro‐level to achieve the desired performance.

### 3.2 Oxide Solid‐State Electrolytes

#### 3.2.1 Introduction of Oxide Solid‐State Electrolytes

The first oxide‐based super‐ionic conductor compound with a general formula Na_1+x_Zr_2_P_3‐x_Si_x_O_12_ (0 <x<3) was reported by Goodenough and Hong as early as 1976.^[^ ^286^, ^287^ ^]^ Within the system of this skeleton structure, both NaZr_2_P_3_O_12_ and Na_3_Zr_2_Si_2_PO_12_ were identified to possess a rigid, linked 3D interstitial space and have PO_4_ or SiO_4_ tetrahedra sites that share corners with the ZrO_6_ octahedra sites. The intrinsic properties of these structures facilitate enhanced, fast, reversible alkali‐ion transportation (Li^+^, Na^+^, K^+^, etc.). After decades of research and development, the first application of oxide SSE in the ASSLIB system of Li_4_Ti_5_O_12_/Li_1.3_Al_0.3_Ti_1.7_(PO_4_)_3_/LiMn_2_O_4_ was reported by Birke's group in 1998.^[^ ^288^ ^]^ A 15% weight percentage sintering additive (0.44 LiBO_2_ • 0.56 LiF) was employed within the LMO cathode to minimize side reactions that were induced by high‐temperature annealing; meanwhile, the additive in the LATP electrolyte functioned in elevating the conductivity to more than 10^−4^ S cm^−1^. Notably, even though a pronounced polarization effect was discerned in the first cycle, the ASSLIBs still implemented excellent capacity retention within the first 10 cycles. This work demonstrated the feasibility of applying oxide SSEs in ASSLIBs. Inspired by this work as the pioneer of the oxide electrolyte‐based ASSLIBs, the oxide SSEs, which possess wide electrochemical stability windows, excellent air stability, and robust mechanical strength, have spurred accumulated attention and have been widely studied since then.^[^ ^20^, ^112^ ^]^

Generally, oxide electrolytes can be categorized into four types: perovskite, garnet, NASICON, and LISICON. A perovskite‐type SSE normally possesses a general formula of ABO_3_. In early 1953, the first perovskite‐type oxide electrolyte Li_1/2_La_1/2_TiO_3_ (LLTO) was reported by Brous et al.^[^ ^289^ ^]^ By manipulating the extent of Li‐ion substitution, Inaguma et al.^[^ ^290^ ^]^ prepared Li_3x_La_2/3‐x_□_1/3‐2x_TiO_3_ (where □ represents a vacancy) SSE with superior room‐temperature Li‐ion conductivity of 1 × 10^−3^ S cm^−1^ and electric conductivity of 7.5 × 10^−5^ S cm^−1^. Attracted by the great potential as an excellent candidate of oxide SSE for ASSLIBs, a diversity of studies and investigations were conducted to delve into the understanding of the structures and electrochemical mechanisms of the LLTO. It was perceived that the crystal lattice and Li‐ion concentrations were two fundamental factors in determining ionic conductivity. The tetrahedral structure of LLTO with Li‐rich exhibited exceptional Li‐ion conductivity with the help of the formed 3D Li‐ion conduction channels. In contrast, Li‐poor and high La occupancy within the orthorhombic LLTO structure resulted in inferior ionic transport parameters.^[^ ^291^, ^292^, ^293^, ^294^ ^]^

Another widely used garnet‐type oxide SSEs can be written in the form of A_3_B_2_(XO_4_)_3_ as the general chemical formula. Garnet‐type oxide SSEs are generally crystallized in the face‐centered cubic structure.^[^ ^112^ ^]^ The garnet‐type Li_3_La_3_Te_2_O_12_ was first introduced by Cussen et al.^[^ ^295^ ^]^ and discovered to possess low Li‐ion conductivity. Therefore, for the purpose of elevating the ionic conductivity by increasing the Li content, Li_3_,^[^ ^296^ ^]^ Li_5_,^[^ ^297^, ^298^ ^]^ Li_6_,^[^ ^299^ ^]^ and Li_7_^[^ ^300^, ^301^, ^302^ ^]^ types of garnet‐type oxide SSEs were developed. Because of the intrinsic facile Li‐ion transport properties of the octahedral site within Li_5_‐Li_7_, the number of lithium occupancies in this site plays an essential role in determining Li‐ion conductivity. Furthermore, Li_7_ phase garnet‐type SSE typically possesses either cubic or tetrahedral polymorph structures. The Li‐ions in the cubic LLZO are completely disordered but fully ordered in tetrahedral LLZO. As a result, the lithium ionic conductivity in cubic LLZO demonstrates 1–2 orders of magnitude higher than that of tetrahedral LLZO.^[^ ^20^, ^112^ ^]^ Currently, the highest ionic conductivity of garnet‐type oxide SSE Li_6.55_Ga_0.15_La_3_Zr_2_O_12_ could reach 2.06 × 10^−3^ S cm^−1^ at room temperature.^[^ ^303^ ^]^ Garnet‐type oxide electrolytes possess a wide electrochemical stability window of 0–6 V,^[^ ^304^, ^305^ ^]^ and the stability against Li metal that enables pairing with the Li metal anode in the ASSLIBs.^[^ ^306^ ^]^ From the computational DFT calculation and thermodynamic stability evaluation, Cedar et al.^[^ ^195^ ^]^ delineated the most well‐discerned garnet‐type Li_7_La_3_Zr_2_O_12_ electrolyte, which exhibited a wide electrochemical stability window of 0.05–3.30 V^[^ ^304^ ^]^ and high ionic conductivity of 3 × 10^−4^ S cm^−1[^ ^302^ ^]^ at room temperature since it possessed the chemical similarity to LiAlO_2_.^[^ ^307^ ^]^

NASICON, which is short for sodium (Na) superionic conductor with a general formula of AM_2_(PO_4_)_3_, is another well‐recognized oxide SSE.^[^ ^308^, ^309^, ^310^, ^311^ ^]^ The most commonly exploited NASICON SSEs are LiTi_2_(PO_4_)_3_ (LTP) and LiGe_2_(PO_4_)_3_ (LGP). The rigid framework of M_2_P_3_O_12_ could facilitate extra MO_6_ octahedra and PO_4_ tetrahedra channels, which are beneficial for Li‐ion diffusion transportation. Unfortunately, even with the additional Li‐ion pathways, the NASICON‐type SSEs possess relatively low ionic conductivity, for instance, 10^−8^–10^−6^ S cm^−1^ for LTP at 50 °C.^[^ ^312^, ^313^ ^]^ NASICON SSEs exhibit low electronic conductivity due to the overlapping of the electronic orbitals between the octahedral MO_6_ and corner‐sharing tetrahedral PO_4_ within the framework. Additionally, through the modifications of Al^3+^ aliovalent substitutions, the derived Li_1.3_Al_0.3_Ti_1.7_(PO_4_)_3_ (LATP) and Li_1+x_Al_x_Ge_2‐x_(PO_4_)_3_ (LAGP) exhibit increased Li^+^ concentration plus the additional diffusional pathways within the structure, thus significantly improving the ionic conductivity to the level of ≈10^−3^ S cm^−1^ at room temperature.^[^ ^314^, ^315^, ^316^, ^317^ ^]^

Similar to NASICON, LISICON, as the prevailing last type of oxide SSE, adopted its name from lithium superionic conductors. Hong's group, for the first time, reported this LISICON with the typical formula as Li_16‐2x_D_x_(TO_4_)_4_ (0 < x < 4), where D stands for divalent cations, such as Mg^2+^ or Zn^2+^, and T represents tetravalent cations, for instance, Si^4+^ or Ge^4+^. Li_14_Zn(GeO_4_)_4_ (LZGO), as a typical one of these, with the favorable 3D Li‐ion transport channel in the fundamental structure, exhibited ionic conductivity of ≈10^−7^ S cm^−1^ at room temperature and 0.125 S cm^−1^ at 300 °C.^[^ ^318^ ^]^ In some cases, LISICON‐type SSEs Li_3+x_X_x_Y_1‐x_O_4_ could be considered as a combination of Li_4_XO_4_ (X = Si, Ge, Ti, etc.) and Li_3_YO_4_ (Y = P, As, V, etc.).^[^ ^319^, ^320^ ^]^ Li_3.5_Si_0.5_P_0.5_O_4_, as a typical example of this series, possesses a relatively moderate ionic conductivity of 3 × 10^−6^ S cm^−1^.^[^ ^319^ ^]^

#### 3.2.2 Challenges of Coupling with Mn‐Based Cathodes

##### Temperature‐Derived Interfacial Resistance Challenges

It is important to note that the distinction between the *interface* and the *interphase* is crucial when evaluating electrode‐electrolyte contact in solid‐state batteries.^[^ ^19^ ^]^ Generally, the interface refers to the physical boundary that was initially formed between the electrode and the electrolytes when the two components are in contact with each other. For instance, the Mn‐based spinel cathodes (LMO or LNMO), the SSEs, and/or conductive additives. Ideally, an intimate, clean, and thermodynamically and kinetically stable cathode/SSE interface with minimal impedance is highly desired to facilitate fast and efficient ion and electron transport.^[^ ^196^, ^321^ ^]^ In contrast, the interphase represents a chemically, electrochemically, and structurally evolved region that forms due to chemical interdiffusion, electrochemical reactions, and mechanical stresses. These formed interphases that result from, for instance, Mn migration, oxygen loss, or SSE decomposition, and may exhibit amorphous, polycrystalline, or defect‐rich structures. It should be noted that the formed interphases can be either beneficial (by forming ion‐conductive protective layers) or detrimental (by blocking transport, causing cathode degradation and structural failure) to the overall electrochemical performance.^[^ ^322^, ^323^ ^]^ Therefore, understanding, controlling, and engineering both of them are essential for reducing interfacial impedance and improving battery stability.

The existence of large interfacial resistance between the Mn‐based spinel cathode and the oxide SSEs is still a critical challenge in the applications of ASSLIBs.^[^ ^324^ ^]^ It was revealed from the previous studies that such challenges are induced by:

1) the decomposition of the electrode/electrolyte interphases under high temperature or high voltage,^[^ ^325^, ^326^ ^]^
2) the formation of the space‐charge effects,^[^ ^327^, ^328^ ^]^ and
3) the contact loss due to the structure/volume changes during the lithiation and delithiation process,^[^ ^171^, ^329^, ^330^, ^331^, ^332^ ^]^ or the combination of these factors.

Due to the intrinsic stiffness of the oxide SSEs and the fabrication method used, solid‐state sintering between the cathode and the oxide SSEs is always demanded to fabricate an intimate and well‐contacted interface.^[^ ^333^, ^334^, ^335^ ^]^ Unfortunately, at elevated temperatures, the associated formation of the impurities and the induced large impedance interphases result in sluggish Li‐ion transportation kinetics and a reduction in discharge capacity.^[^ ^19^, ^336^ ^]^ It should be prudent to note that, even though the Mn‐based spinel cathodes and the coupled oxide SSEs could tolerate high‐temperature heating without undergoing decompositions, the thermal instability of the interface is the origin of the large impedance. Such instability was reaffirmed through both computational and experimental studies. From experimental results, it was observed that the stability of the LMO/Al‐LLZO interfaces could be preserved as high as 400 °C,^[^ ^306^ ^]^ and the formation of the side products was observed at 600 °C at the interface between LMO and Al‐LLZO. Meanwhile, from the thermodynamic simulation, Raskovalov et al.^[^ ^337^ ^]^ indicated that the pure LMO phase at the CEI could only exist at room temperature. When increasing the temperature (≥ 400 K), unfavorable products such as Li_4_Mn_5_O_12_, LaMnO_3_, Li_8_ZrO_6_, and La_2_ZrO_7_ were predicted to form due to the LMO/LLZO interactions at the interface. Howard's group^[^ ^338^ ^]^ obtained similar results from the computational study of the thermodynamic stability at the interface between LMO/LLTO. The simulation results indicated the interface was thermodynamically reactive; consequently, the thermodynamically favored interdiffusion of Ti and Mn commenced as low as 0 K. The interdiffusion at the interface triggered the formation of unfavored large interfacial resistance. In addition, the DFT results suggested the significant entropy‐driven Ti‐Mn interdiffusion at the synthesis temperature. Experimental tests were conducted by depositing the LMO layer on top of the LLTO layer to validate the DFT calculation further and investigate the relationship between interfacial reactivity and temperature. The temperature range of 600–700 °C emerged as critical since severe interdiffusion between LLTO and LMO was observed, which led to noticeable blurring with the formation of interphases.

**Figure 7:**
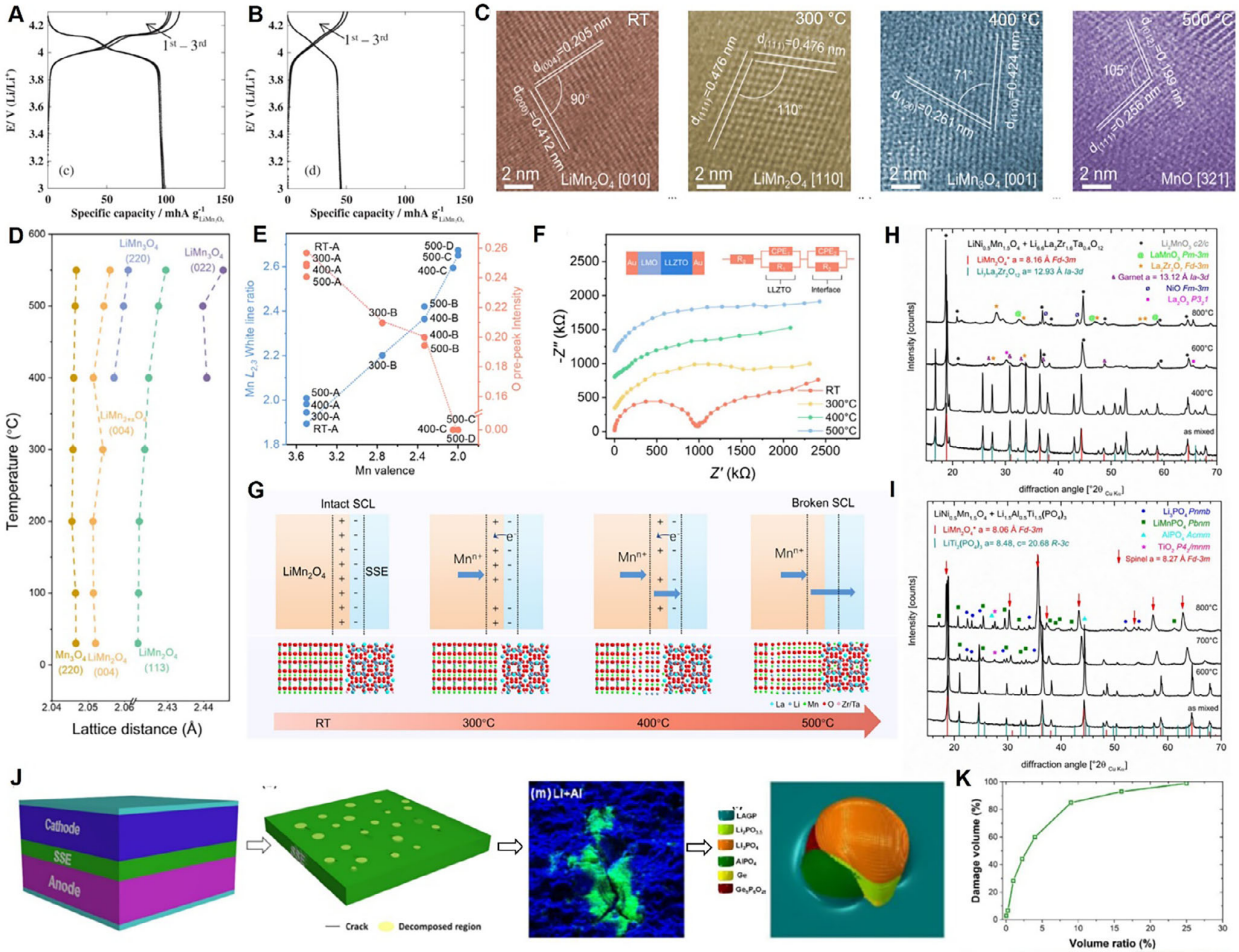
A,B) Typical charge‐discharge profile of LMO//Al‐LLZ before treatment and post‐treatment, respectively. Reproduced with permission.^[^ ^306^ ^]^ Copyright 2017, The Electrochemical Society of Japan. C) HRTEM images at LMO//LLZTO interface at different temperatures (RT, 300 °C, 400 °C, and 500 °C). D) Lattice parameter changes of LMO cathodes when heated to different temperatures. E) Valence changes of Mn at Mn‐L_2,3_ edge and O‐K edge at the interface of LMO//LLZTO at different temperatures. F) EIS tests of Au//LMO//LLZTO//Au at different temperatures. G) Schematic of the mechanism of the space charge layer at the LMO//LLZTO interface. Reproduced with permission.^[^ ^340^ ^]^ Copyright 2023, Wiley. H,I) XRD patterns of LNMO & Ta‐LLZO and LNMO & LATP at different temperatures, respectively. Reproduced with permission.^[^ ^336^ ^]^ Copyright 2016, American Chemical Society. J) Schematic of electromechanical failure of LNMO‐based cathode, LAGP SSE, MnO_2_‐based anode ASSLIBs. K) Decomposition regions vs decomposition volume ratios of LAGP after long cycling. Reproduced with permission.^[^ ^348^ ^]^ Copyright 2021, American Chemical Society.

Weppner's group examined the chemical compatibility of the LMO and the garnet‐like oxide SSE—Li_6_BaLa_2_Ta_2_O_12_ at various temperatures through the powder X‐ray diffraction (XRD) technique.^[^ ^339^ ^]^ It was observed that the SSE was not chemically stable and started to decompose at 400 °C, which was associated with more accelerated side reactions. Consequently, the garnet‐like Li_6_BaLa_2_Ta_2_O_12_ transformed into the thermodynamically more stable, ordered perovskite La_2_LiTa_2_O_6_ at the elevated temperature. The EIS test confirmed that the formed La_2_LiTa_2_O_6_ resulted in enlarged impedance, leading to sluggish Li‐ion mobility. From the study of Kanamura et al.,^[^ ^306^ ^]^ the thermal stability of the LMO cathode with the cubic garnet structure oxide SSE Li_6.25_Al_0.25_La_3_Zr_2_O_12_ (Al‐LLZO) was investigated. With the increasing temperature, it was observed that the peak intensity of Al‐LLZO of the XRD pattern decreased and eventually completely disappeared at 800 °C. The formation of side‐reaction products, such as Li_2_MnO_3_, La_2_Zr_2_O_7_, La_2_O_3_, and LaMn_0.8_O_3,_ further evidenced the decomposition of Al‐LLZO and the reactions between LMO and Al‐LLZO. Consequently, the specific discharge capacity was significantly reduced from 100 mAh g^−1^ to under 50 mAh g^−1^, as shown in **Figure** **7**A,B. Gellert et al.^[^ ^324^ ^]^ screened and evaluated the thermal compatibility of the LATP SSE and several cathode materials during the cosintering process. The pristine LATP was revealed to be relatively stable up to 800 °C with minimal generation of AlPO_4_, but a noticeable amount of AlPO_4_ in the 900 °C sample. When cosintering LMO with LATP, the phase changes were observed at a low temperature of 600 °C with the formation of Mn_2_O_3_. At 700 °C, the decomposition of LMO became conspicuous and was associated with the observation of Li_3_PO_4_ and AlPO_4_. The formation of AlPO_4,_ which originated from the exsolution of Al^3+^ from LATP, led to the valence change from Ti^4+^ (ionic conductivity = 10^−3^ S m^−1^) to Ti^3+^ (ionic conductivity = 10^−7^ S m^−1^), and consequently, elevated grain boundary resistance.^[^ ^312^ ^]^

Su et al.^[^ ^340^ ^]^ systematically studied thermal stability and structure evolution of the interface between LMO and LLZTO at different temperatures through various in‐situ techniques. The Mn diffusion from LMO to LLZTO was observed starting from 300 °C, which was associated with the formation of LiMn_3_O_4_ at the interface. When the temperature was elevated to 500 °C, the formed LiMn_3_O_4_ phase transformed into MnO, restricting Li‐ion transportation and enlarging the interfacial impedance. The formation of the impurity with the increase of the temperatures at the LMO and LLZTO interface was indicated in the images of HRTEM, as shown in Figure 7C. The variation of lattice parameters is shown in Figure 7D, further reaffirming the formation of the impurity at different temperatures. EELS spectra of Mn‐L_2,3_ edges and O‐K edge at the LMO and LLZTO interface were conducted. The Mn valence was quantitatively analyzed, where the Mn exhibited +3.5 at room temperature and reduced to +2 at 500 °C, indicating the transformation to MnO (Figure 7E). EIS measurements revealed that the 2 semicircles observed at room temperature for the LMO and LLZTO samples merged into one semicircle, showing the enlarged interfacial resistance with the elevation of the temperatures (Figure 7F). Moreover, the impacts of the space charge layer at the interface were also investigated. Generally, the formation of the SCL originated from the difference in the chemical potential between the cathode and the SSEs.^[^ ^341^, ^342^ ^]^ Such a layer induced large interfacial impedance and sluggish Li‐ion transportation. In this study, at room temperature, the spontaneously formed space charge layer functioned as a barrier to prevent Mn in the cathode from migrating toward the Mn‐deficient electrolyte side.^[^ ^343^, ^344^ ^]^ At 300 °C, Mn‐ions with high kinetic energy were restrained by the SCL barrier and accumulated at the surface of the SCL, resulting in extensively increased interfacial resistance and reduced ionic conductivity. At a high temperature of 500 °C, thermodynamically energized Mn‐ions broke through the SCL barrier and migrated to SSE. Consequently, Mn ions occupied Li sites and led to the irreversible phase transformation from LiMn_2_O_4_ into LiMn_3_O_4_ and eventually into MnO, as illustrated in Figure 7G.

Cedar et al.^[^ ^336^ ^]^ investigated the decomposition reactions between LNMO and garnet‐type SSE (Ta‐LLZO) and NASICON‐type SSE (LATP) during the high‐temperature co‐sintering process. In this study, both ab initio calculations and thermal analysis techniques were employed to elucidate the failure mechanism of the cathode/oxide SSE composite mixtures. It was observed that Ta‐LLZO commenced to decompose at a temperature of 600 °C. The formed insulating phases with side products of La_2_Zr_2_O_7_, La_2_O_3_, La_3_TaO_7_, NiO, and LaMnO_3_ remarkably increased the interfacial impedance, as shown in Figure 7H. The formed highly stable Li_2_MnO_3_ originated from oxygen loss from the cathode and was absorbed by Ta‐LLZO. Due to the large reactivity between LATP and LNMO, the intensities of both phases from XRD results were significantly decreased at the temperature of 700 °C when compared with the co‐sintering temperatures of 600 °C and completely disappeared at 800 °C. The side products, such as Li_3_PO_4_, AlPO_4_, LiMnPO_4,_ etc., were observed as shown in Figure 7I. Interestingly, the decomposed product, Li_3_PO_4_, possessed reasonable ionic conductivity that could still maintain the functionality of the battery instead of failure.

##### Electrochemical Incompatibility Under High Voltages

Beyond the significant challenges of highly unfavorable decompositions of the electrode and electrolyte during high‐temperature co‐sintering, the decompositions of the oxide SSEs due to their incompatibility with high‐voltage spinel cathodes nevertheless needed to be considered. The decomposition of the electrolytes not only produces side products that deteriorate ion transport capabilities and the cell capacity but also results in battery dysfunction.

The incompatibility of the nano‐grain synthesized Li_6.4_Ga_0.2_La_3_Zr_2_O_12_ (LGLZO) pairing with the high voltage LNMO cathode was reported by Rupp's group.^[^ ^345^ ^]^ The ASSLIBs undertook charging and discharging cycle testing under 95 °C between 3.0 V and 4.9 V (vs Li/Li^+^). Notably, a severe potential plunge was observed at 3.8 V during the initial charging process, indicating the severity of the irreversible reactions between LNMO and LGLZO. Consequently, the battery could not maintain any electrochemical performance in the following cycles. Further modifications to the pellet density and sintering temperature‐controlled interface showed no improvement in electrochemical performance. Such observations confirmed that the failure of the ASSLIBs was directly related to the incompatibility of the LNMO and LGLZO, regardless of the fabrication method. The XRD result of the cycled battery confirmed the formation of the side products Li_0.35_Ni_0.05_NiO_2_ and Li_2_MnO_3_, and small amounts of unable‐to‐identify binary compounds that originated from the Li‐ions loss from the electrolyte and resulted in the formation of the new phases. These findings were also in line with the previous theoretical DFT calculation by Cedar's group and Zhu's group^[^ ^346^ ^]^ that the LLZO possessed an intrinsic stability window up to 3.8 V.

Adams et al.^[^ ^348^ ^]^ investigated the decomposition of NASICON‐type SSE (LAGP), when coupled with LNMO from both DFT calculations and experimental evaluations. During the room temperature cycling process, in order to reach the thermodynamic equilibrium within the LAGP, the free surface electrons accelerated the decomposition of LAGP and the formation of side products such as AlPO_4_, L_4_P_2_O_7_, GeO, etc. (Figure 7J). A grain size reduction and volume expansion were also detected during cycling. After 80 cycles, the crack length on the LAGP was measured at 5 µm with the accumulation of Li and P in the decomposed LAGP area, as shown in Figure 7K.

#### 3.2.3 Engineering Interfacial Contact

As discussed in the above section, the rigid nature of oxide SSEs presents persistent challenges in maintaining close interfacial contact with cathodes. This results in a limited contact area and severe polarization effects that significantly hinder Li‐ion transport. Therefore, it is indispensable to address this challenge to realize satisfactory electrochemical performance when pairing a Mn‐based spinel cathode with oxide SSEs. Strategies were implemented to overcome the challenges, including incorporating advanced deposition methods, tuning microstructure, and introducing flexible polymer electrolytes to enhance the interfacial contact between the cathode active material and the oxide SSEs.

The distinction between the *interface* and the *interphase* is crucial when evaluating electrode‐electrolyte contact in solid‐state batteries. The interface refers to the physical boundary between the electrode and electrolyte, ideally clean and well‐matched, to facilitate fast ion transport. In contrast, the interphase represents a chemically or structurally modified region that forms due to interdiffusion or side reactions, which can either enhance or hinder electrochemical performance. Understanding and controlling both are essential for engineering interfacial contact between the electrode and electrolyte, reducing interfacial impedance, and improving battery stability.

**Figure 8:**
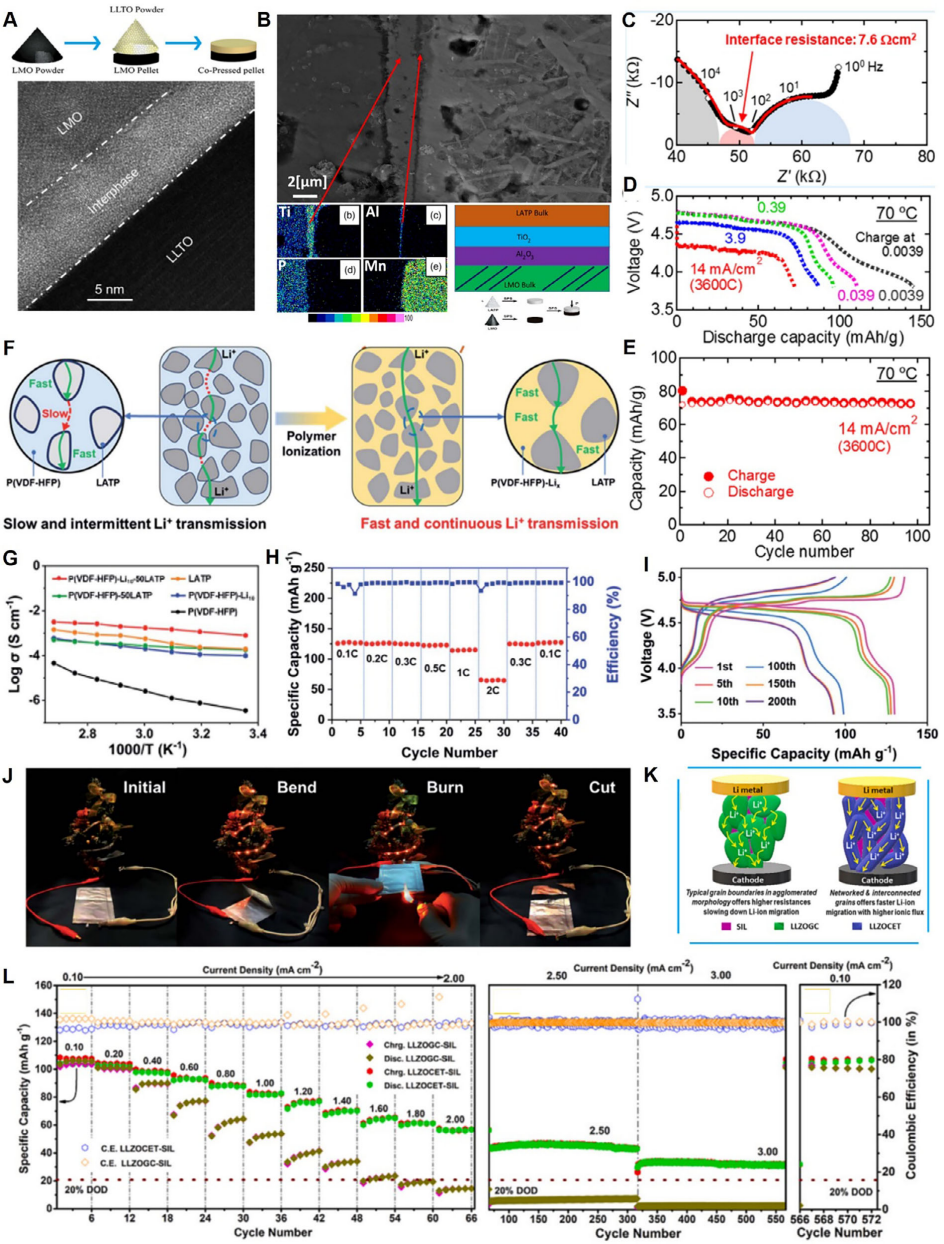
A) Schematic of co‐press LMO cathode and LLTO oxide electrolyte (top) and HRTEM image of LMO and LLTO interfacial region (bottom). Reproduced with permission.^[^ ^93^ ^]^ Copyright 2019, Wiley. B) SEM image at the LMO and LATP interface and the corresponding EDS mapping. The schematic of the interfacial structure at the LMO and LATP interface (bottom right). Reproduced with permission.^[^ ^353^ ^]^ Copyright 2020, Wiley. C) EIS fitting results of the LNMO and LPO interface at 4.7 V. D) Electrochemical discharge performance at different current rates of LNMO/LPO solid‐state batteries. E) Cycling electrochemical performance of LNMO/LPO solid‐state batteries under 3600C. Reproduced with permission.^[^ ^370^ ^]^ Copyright 2018, American Chemical Society. F) Schematic of enhanced Li‐ion transportation pathways with P(VDF‐HFP)‐Li_x_ hybrid electrolyte. G) Arrhenius plots of Ionic conductivity for different electrolytes. H) Rate capability of solid‐state batteries with LNMO cathodes and a hybrid solid‐state electrolyte. I) Typical charge‐discharge profile for LNMO//HSE//Li solid‐state batteries under 0.5C and 25 °C. J) Bend, burn, and cut tests for LNMO//HSE//graphite soft‐packed batteries. Reproduced with permission.^[^ ^381^ ^]^ Copyright 2022, The Royal Society of Chemistry. K) schematic of enhanced Li‐ion transportations with LLZO prepared through the CET process. L) Cycling electrochemical performance of LMO//LLZO‐SIL//Li solid‐state batteries under different current densities. Reproduced with permission.^[^ ^386^ ^]^ Copyright 2023, Elsevier.

The advanced deposition methods could sufficiently improve the interface between the electrode and the electrolyte in reducing the challenge of large interfacial impedance. For instance, the spark plasma sintering (SPS) cosintering method,^[^ ^349^, ^350^ ^]^ which employs uniaxial pressure and a pulsed DC, accelerates the sintering rates under a lower processing temperature and shortens sintering time, thereby enhancing the densification and grain size control of high‐density particles.^[^ ^351^, ^352^ ^]^ Stanciu et al.^[^ ^93^ ^]^ investigated the interface between LLTO and LMO via the SPS sintering method (**Figure** **8**A), identified the formation of interphases, and compared it with the interface that was fabricated through the conventional sintering method. Analyzed by SEM and EDS, the samples produced via SPS exhibited highly densified and reduced grain growth, resulting in improved interfacial contact than those fabricated through conventional co‐sintering methods. Two types of interphases were observed via HRTEM: a) a 10–100 µm thick interdiffusion layer caused by Mn and Ti interdiffusion, and b) a nanometer‐scale amorphous complexion interphase where both LMO and LLTO coexisted (see Figure 8A). In contrast, the interface from the traditional sintering method exhibited visible voids and significant interdiffusion of Mn, Ti, and La ions extending as deep as 300 µm. In addition, EIS tests revealed that the interdiffusion‐derived interphases (a) in the SPS samples were the primary cause of the high interfacial resistance, which was 40 times greater than the sum of the individual LMO and LLTO layers. In contrast, the amorphous interphase at the grain boundary exhibited negligible impedance. Notably, although the SPS‐fabricated interface showed improved densification, it still suffered from interdiffusion at the interface, which led to significant interfacial resistance comparable to that observed with conventional sintering methods. Attempts to reduce the SPS co‐sintering temperature in order to alleviate interfacial impedance were met with trade‐offs; specifically, lower temperatures resulted in poor structural densification of the cathode side, which in turn introduced extensive bulk‐related impedance. Thus, Levit et al.^[^ ^353^ ^]^ developed a novel LMO/LATP fabrication method by SPS to obtain two sintered pellets instead of the SPS co‐sintering, as shown in Figure 8B. The fundamental advantage of this method was to enable a lower temperature to bond the two already‐densified pellets. The thickness of the interphases was reduced, and the effective contact area was improved. The obtained smooth interface exhibited negligible impedance, as shown in Figure 8B, demonstrating the effectiveness of the modified fabrication method. Notably, the formation of secondary insulating interphase products such as TiO_2‐x_ and Al_2_O_3‐y_ due to mutual migration of cations (Al^3+^ and Ti^4+^) from the electrolyte and anions (O^2−^) from the cathode still induced a significant interfacial impedance. Further efforts are still required to reduce the mutual diffusion and prevent the structural changes in optimizing the performance of the interface.

In addition to the sintering methods, various thin‐film fabrication techniques have been reported in constructing oxide SSEs‐based ASSLIBs. These advanced deposition techniques not only deliver homogeneous and conformal interfaces between cathode and oxide SSEs but also allow precise interfacial layer control over thickness and morphology. Notable examples demonstrated in Mn‐based cathode for oxide SSEs‐based ASSLIBs include atomic layer deposition (ALD),^[^ ^354^, ^355^ ^]^ pulsed laser deposition (PLD),^[^ ^117^, ^356^, ^357^, ^358^, ^359^ ^]^ radio frequency magnetron sputtering,^[^ ^360^, ^361^, ^362^ ^]^ chemical solution deposition,^[^ ^363^ ^]^ electrostatic integrated assembly,^[^ ^364^, ^365^ ^]^ sol‐gel coating,^[^ ^366^ ^]^ and pulsed spray technique.^[^ ^367^ ^]^ Lethien et al.^[^ ^354^ ^]^ utilized the ALD technique to fabricate a conformal and uniform 3D‐LMO thin film cathode. The constructed interface without detections of interdiffusion, inclusions, or segregation further evidenced the advantages of this deposition method. With the help of 3D scaffold silicon microtubes, the thickness of the LMO thin film was controlled between 100–250 nm. The ASSLIBs with the fabricated 100 nm LMO thin film as cathode and Li_3_PO_4_ as the electrolyte, delivered a superior surface capacity of approximately 0.18 mAh cm^−2^ under 0.05C and excellent cycling stability and capacity retention with Coulombic efficiency of 95% in 50 cycles. Radio frequency magnetron sputtering deposition, followed by annealing, is also a widely used fabrication technique in LNMO thin film ASSLIBs. Lethien et al.^[^ ^362^, ^368^ ^]^ emphasized that deposition pressure was the key factor that influenced the cations' ordering, which directly related to the crystal phases (ordered or disordered), and subsequently, affected the ASSLIBs' electrochemical performance. The sample deposited with a pressure higher than 2.5 × 10^−2^ mbar was pure ordered spinel (P4_3_32), meanwhile, under the critical pressure of 1 × 10^−2^ mbar delivered disordered spinel ($\mathrm{Fd}\bar{3}m$). The formation of Li_2_MnO_3_ detrimental phase associated with O^2−^ loss and the existence of Ni^2+^/Ni^3+^ mixing were detected via Raman spectroscopy.^[^ ^144^, ^164^, ^369^ ^]^ This observation was delineated to be increasing with the reduction of the deposition pressure. In addition, the LNMO films possessed higher density and homogeneous morphologies with lower surface roughness and reduced amount of small grains on the surface when deposited at low pressure (6 × 10^−3^ and 10^−2^ mbar). Meanwhile, the higher pressure (5×10^−2^ and 9×10^−2^ mbar) resulted in more columnar and porous films with significantly rougher surfaces and increased size and number of grains.

Hitosugi's group performed a series of experimental research to systematically study the interface (LPO) between Li_3_PO_4_ SSEs and LNMO electrodes from the perspectives of composition,^[^ ^370^ ^]^ phase transformation,^[^ ^371^ ^]^ and thermal stability.^[^ ^372^ ^]^ In the early work, to avoid the generation of impurities at the interface that were induced by exposure to air, the group fabricated an interface under ultrahigh vacuum (UHV) conditions.^[^ ^370^ ^]^ The interface exhibited an extremely low resistance of 7.6 Ω cm^−2^, as shown in Figure 8C, which was one order of magnitude lower than the LNMO liquid electrolyte‐based batteries.^[^ ^373^ ^]^ After deposition at room temperature, a spontaneous Li‐ion migration from the LPO thin film, across the LPO/LNMO interface, to the LNMO surface was observed, which was attributed to the difference in chemical potential between LPO and LNMO. Consequently, almost half of the LNMO converted to Li_2_Ni_0.5_Mn_1.5_O_4_ phase, which was further evidenced by the peak from the CV test at a voltage of ≈2.9 V during the charging process. At an elevated temperature of 70 °C, the ASSLIBs demonstrated 145 mAh g^−1^ and 73 mAh g^−1^ initial discharge capacity under a current density of 1C, and 3600C, respectively, with stable cycling for 100 cycles due to the formed stable interface (Figure 8D,E). To further understand the spontaneously formed Li_2_Ni_0.5_Mn_1.5_O_4_ phase at the interface between LNMO and LPO from the abovementioned study, Hitosugi's group investigated Li‐ion migration behavior at the interface.^[^ ^371^ ^]^ It was confirmed that the phase transformation from Li_2_Ni_0.5_Mn_1.5_O_4_ to LiNi_0.5_M_1.5_O_4_ and Li_0_Ni_0.5_Mn_1.5_O_4_ during the charging and discharging process was highly reversible and did not possess a pronounced influence on the cycling stability. The ASSLIBs delivered an initial Coulombic efficiency of 58% due to the spontaneous Li‐ion migrations. A stable cycling was reaffirmed by maintaining close to 100% Coulombic efficiency from the second to the 50th cycles. In the third work, the thermal stability of the air‐isolated interface between LPO and LNMO was dissected.^[^ ^372^ ^]^ The interface annealed at 200 °C exhibited a relatively low interface resistance of 7.2 Ω cm^2^, similar to the unannealed interface. In contrast, a 70 times higher interface resistance of 490 Ω cm^2^ was observed at the 450 °C annealed interface, indicating the instability of the interface. Due to the crystallization of LPO at 450 °C, the ionic conductivity of the LPO layer (8.7 × 10^−10^ S cm^−1^) was three orders of magnitude lower than 6.5 × 10^−7^ S cm^−1^ (at 200 °C annealing). The XPS results suggested LNMO could maintain high thermal stability at high temperatures (450 °C) due to the consistency of the chemical states of Ni and Mn in LNMO. Moreover, the observed P 2p peak shift indicated the reduction of P from LPO, which was identified as the origin of the enlarged interfacial resistance and reduced ionic conductivity in addition to the electrolyte crystallization. The fabricated ASSTFBs with 200 °C annealed LNMO/LPO interface delivered an initial discharge capacity of 72 mAh g^−1^ at 1C (3.9 µA cm^−2^). The stabilized interface successfully contributed to high current density tolerance up to 1000C (3900 µA cm^−2^) with a discharge capacity of 8.1 mAh g^−1^, in contrast to the 450 °C annealed interface, which only tolerated up to 100C without the capability of discharge.

The advanced deposition methods significantly improve the interfacial contact between the spinel cathode and the oxide SSEs. Tuning the microstructure of the cathode is another approach to effectively enhance the interfacial contact. An innovative three‐dimensionally ordered microporous (3DOM) electrode system was developed by Kanamura's group.^[^ ^374^, ^375^, ^376^, ^377^ ^]^ A 3DOM‐Li_1.5_Al_0.5_Ti_1.5_(PO_4_)_3_ (LATP) and LiMn_2_O_4_ composite cathode was fabricated by the colloidal crystal templating and followed by the sol‐gel method.^[^ ^377^ ^]^ This 3DOM structure exhibited outstanding advantages of an enlarged contact surface area that enabled filling the LMO within the pores, which facilitated short Li‐ion and electron transportation pathways. The fabricated cells delivered an initial discharge capacity and Coulombic efficiency of 64 mAh g^−1^ and ca. 94%, respectively. Notably, even with 60% of the LMO filling ratio, the ionic and electronic insulating voids persisted and resulted in a relatively low discharge capacity. In a later work, 3DOM‐perovskite‐type Li_0.35_La_0.55_TiO_3_ (LLTO) and LMO composite cathode were developed with the same fabrication method as the previous study, showing an increment to 80% of the LMO filling ratio.^[^ ^375^ ^]^ The ASSLIBs delivered an initial discharge capacity of 82 mAh g^−1^ with a Coulombic efficiency of 75% at a current density of 25 µA cm^−2^, which was attributed to the low conductivity of the composite cathode and/or the electrolyte degradation during the cycling process. The remaining challenge of existing voids in the composite cathode that restrained Li‐ion transportation was still unsolved in this study. A novel porous honeycomb structure was introduced to further improve the 3DOM structure.^[^ ^376^ ^]^ With the micro‐sized holes on one side, the LLTP SSE honeycomb structure enabled a higher cathode active material loading within by inserting the 3DOM porous LLTO electrolyte into the holes before filling LMO in the pores, thus escalating the contact surface area.^[^ ^378^, ^379^ ^]^ This innovative 3D design achieved a high LMO filling ratio of 70%. Consequently, the fabricated ASSLIBs delivered an initial reversible discharge capacity of 71 mAh g^−1^, which was 1.6 times enhanced than previous work (42.2 mAh g^−1^).^[^ ^379^ ^]^ Notably, the cathode active materials at the center of the holes still suffered from long Li‐ion transportation paths, which still required further improvement to enhance the electrochemical performance.

#### 3.2.4 Facilitate Li‐Ion Transportation at the Cathode/SSE Interfaces

Benefiting from the rigid mechanical properties of oxide electrolytes and the flexibility and wettability of organic additives, hybrid SSEs employ polymer solid‐state electrolytes to substantially improve the interfacial contact between low‐flexibility ceramic SSEs and the cathode. And therefore, it could significantly enhance the electrochemical performance of the ASSLIBs. For instance, Guo et al.^[^ ^380^ ^]^ designed a novel heterogeneous multilayer structured hybrid SSE by incorporating high content LAGP (80 wt.%) with high‐voltage resistant PAN to the cathode side and Li‐stable PEGDA to the anode side. The hybrid SSE demonstrated an enlarged electrochemical stability window up to 5.0 V with room‐temperature ionic conductivity of 3.7 × 10^−4^ S cm^−1^. Yan et al.^[^ ^381^ ^]^ reported a Li_1.4_Al_0.4_Ti_0.6_(PO_4_)_3_ (LATP)‐based hybrid SSE by incorporating an anionic‐type single Li‐ion conductor—lithium taurine‐grafted poly(vinylidene fluoride‐co‐hexafluoropropylene (P(VDF‐HFP)‐Li_10_, as shown in Figure 8F. Functioning as a transporting bridge, this polymer Li‐ion conductor provided a fast and continuous Li‐ion pathway and prevented the space charge effect accumulation at the polymer and oxide electrolytes interface, hence improving Li‐ion migration efficiency with an excellent ionic conductivity of 7.88 × 10^−4^ S cm^−1^ (Figure 8G). The ASSLIBs with LNMO as cathode exhibited a specific discharge capacity of 126.8 mAh g^−1^ at 0.5C with almost 100% Coulombic efficiency and 59.8 mAh g^−1^ at 2C, as shown in Figure 8H. In addition, the batteries achieved 75% capacity retention after 200 cycles (Figure 8I), further proving the stability of the interfaces. From a safety perspective, the soft‐packed batteries maintained stable operation under bend, burn, and cut tests, as shown in Figure 8J, which demonstrated highly desired enhanced safety in future energy storage applications.

When exposed to the ambient atmosphere, the cubic LLZO undergoes surface reactions, leading to Li^+^ and H^+^ ion‐exchange, and forms poor ionic conductive Li_2_CO_3_. Additionally, the impurities, such as La_2_O_3_ and LiAlO_2_, are generated along with the formation of tetragonal LLZO, which exhibits reduced ionic conductivity.^[^ ^382^, ^383^, ^384^ ^]^ Raja's group,^[^ ^385^ ^]^ for the first time, developed a unique fine combustion method to suppress lithium loss during high‐temperature synthesis and subsequent surface reactions, reduce the formation of impurities, and enhance the ionic conductivity of the electrolyte. This method involved precisely controlled synthesis temperature below 1000 °C and engineering the morphological structure of LLZO. Following, the hybrid SSE was impregnated with a solvated ionic liquid to increase the wettability. When paired with LMO, the ASSLIBs demonstrated electrochemical performance with an average discharge capacity of 104.91 mAh g^−1^ and a Coulombic efficiency of 98% under a current density of 0.05 mA cm^−2^ (0.25 C). The ASSLIBs also possessed excellent rate capability and long‐cycle stability that could tolerate 1.2 mA cm^−2^ (6C) and retain almost 100% capacity when the rates were gradually reduced back to 0.2 mA cm^−2^ (1C). The fast ionic transportation originated from 1) a multifaceted microstructure that significantly reduced the grain boundary area, and 2) a solvated ionic liquid that filled the void of LLZO and further enhanced ionic conductivity. In the later work, Raja's group demonstrated another microstructure‐engineered LLZO‐based solid‐state hybrid electrolyte to reduce grain boundary resistance and elevate Li‐ion conductivity.^[^ ^386^ ^]^ The electrolyte was developed by firstly doping Ga into LLZO and forming Li_6.25_La_3_Ga_0.25_Zr_2_O_12_ (Ga‐LLZO) pellet, then infusing the pellet with SIL and followed by cellulose exo‐templating. Such modifications facilitated facile Li‐ion transport pathways within the electrolyte than the conventional LLZO with large grain boundary resistance, as shown in Figure 8K. The ASSLIBs with LMO cathode demonstrated excellent long‐cycle stability, which could retain 95% of initial discharge capacity after more than 570 cycles (Figure 8L). At a current density of 3.0 mA cm^−2^, the battery could still deliver a discharge capacity of 32.87 mAh g^−1^, which was equivalent to 31.65% of the initial discharge capacity.

Lu's group^[^ ^387^ ^]^ employed a novel lithium ionic conductive binder, PALLi, between the LNMO cathode and LAGP electrolyte. Such a Li salt binder could sufficiently bind the cathode with the electrolyte at room temperature to eliminate the undesired interfacial reactions or TM diffusions which was induced by the conventional high‐temperature annealing. In addition, the PALLi binder effectively reduced the interfacial resistance of LNMO/LAGP from 1.47 × 10^4^ Ω to 190.8 Ω while improving the total ionic conductivity to 1.03 × 10^−4^ S cm^−1^ at room temperature. The maximum discharge capacity of 87.5 mAh g^−1^ was achieved at 0.2C under room temperature and increased to 146 mAh g^−1^ at 0.5C after several cycles when operated at 50 °C.

To summarize, attracted by the advantages of possessing a wide electrochemical stability window and excellent mechanical strength, the oxide SSEs in the applications of ASSLIBs are widely studied and developed. When pairing with Mn‐based cathodes, the challenges of poor interfacial contact between the cathode and the rigid oxide SSEs and the side reactions associated with unfavorable phase transition during the high‐temperature (co)sintering need to be diligently scrutinized and engineered to achieve enhanced electrochemical performance. To address the challenges mentioned, strategies such as introducing organic electrolytes, tuning the microstructure, and reducing the particle size are needed to enhance the interface contacts between the cathode and the oxide SSEs. In addition, incorporating advanced deposition methods facilitates reduced fabrication temperatures while maintaining excellent interfacial contacts. The remaining challenges of oxide SSEs when coupled with Mn‐based cathodes, such as the formation of unfavorable conductive‐free voids at the interface, the large interfacial resistance, and the electrochemical incompatibility between the oxide‐based electrolytes and the high‐voltage spinel cathodes, still need to be addressed.

### 3.3 Composite Solid‐State Electrolytes

To mitigate the challenge of poor ionic conductivity of polymer SSEs at room temperature due to the crystallinity of the polymer host matrix and to enhance their mechanical strength, the composite electrolytes were proposed to be a promising solution to enable their potential employment in ASSLIBs.^[^ ^210^, ^388^, ^389^, ^390^ ^]^ Generally, the solid‐state composite electrolyte comprises polymer electrolytes, lithium salt, and inorganic fillers.^[^ ^391^, ^392^ ^]^ Besides the typical ion‐conducting polymer electrolyte—PEO as the polymer matrix host, other excellent polymer hosts, such as polyvinylidene fluoride (PVDF), polyacrylonitrile (PAN), polyvinyl alcohol (PVA), poly(vinyl carbonate) (PVC), poly(propylene carbonate) (PPC), polymethyl methacrylate (PMMA), etc. are also widely used in designing solid composite electrolytes.^[^ ^219^, ^230^, ^231^, ^393^, ^394^, ^395^, ^396^, ^397^, ^398^ ^]^

It is pivotal to understand that Li‐salt and the inorganic fillers function differently in enhancing the performance of the polymer host. Adding lithium salts plays a prominent role in improving the electrochemical/thermal stability and the ionic conductivity of the solid polymer electrolyte host(s).^[^ ^231^ ^]^ The most commonly utilized lithium salts encompass LiN(CF_3_SO_2_)_2_ (LiTFSI),^[^ ^399^, ^400^, ^401^, ^402^ ^]^ LiN(SO_2_F)_2_ (LiFSI),^[^ ^403^ ^]^ and LiClO_4_.^[^ ^398^, ^404^, ^405^ ^]^ Meanwhile, the inorganic fillers promote Li‐salt dissolution and host polymer plasticization. The inorganic fillers are typically categorized into active and passive types depending on ion‐conducting capabilities.^[^ ^390^, ^406^ ^]^ Oxide compounds, such as SiO_2_,^[^ ^407^, ^408^, ^409^, ^410^, ^411^ ^]^ Al_2_O_3_,^[^ ^412^, ^413^, ^414^ ^]^ etc., are classified as passive fillers. Even though these passive fillers do not possess ionic conductivity, they can nevertheless effectively enhance ionic conductivity by promoting the plasticization of the polymer matrices into disordered crystalline. For instance, Plichta et al.^[^ ^415^ ^]^ elucidated the effectiveness of three types of nanosized fillers: TiO_2_ (11 nm), Al_2_O_3_ (5.8 nm), and SiO_2_ (7 nm) in elevating the ionic conductivity of PEO‐based solid composite electrolytes. At 60 °C, TiO_2_ delivered the greatest improvement of 10^−3^ S cm^−1^, than Al_2_O_3_ of 10^−4^ S cm^−1^ and filler‐free of 10^−5^ S cm^−1^. The enhancement of the ionic conductivity was attributed to the Lewis acid interactions at the surface of PEO chains, the salt dissociation, and the promoted ionic conducting pathways. In addition to the abovementioned oxide compounds, some carbon materials^[^ ^416^, ^417^ ^]^ and metal–organic frameworks (MOFs)^[^ ^418^, ^419^ ^]^ that are applied as passive fillers are also well‐studied.

**Figure 9:**
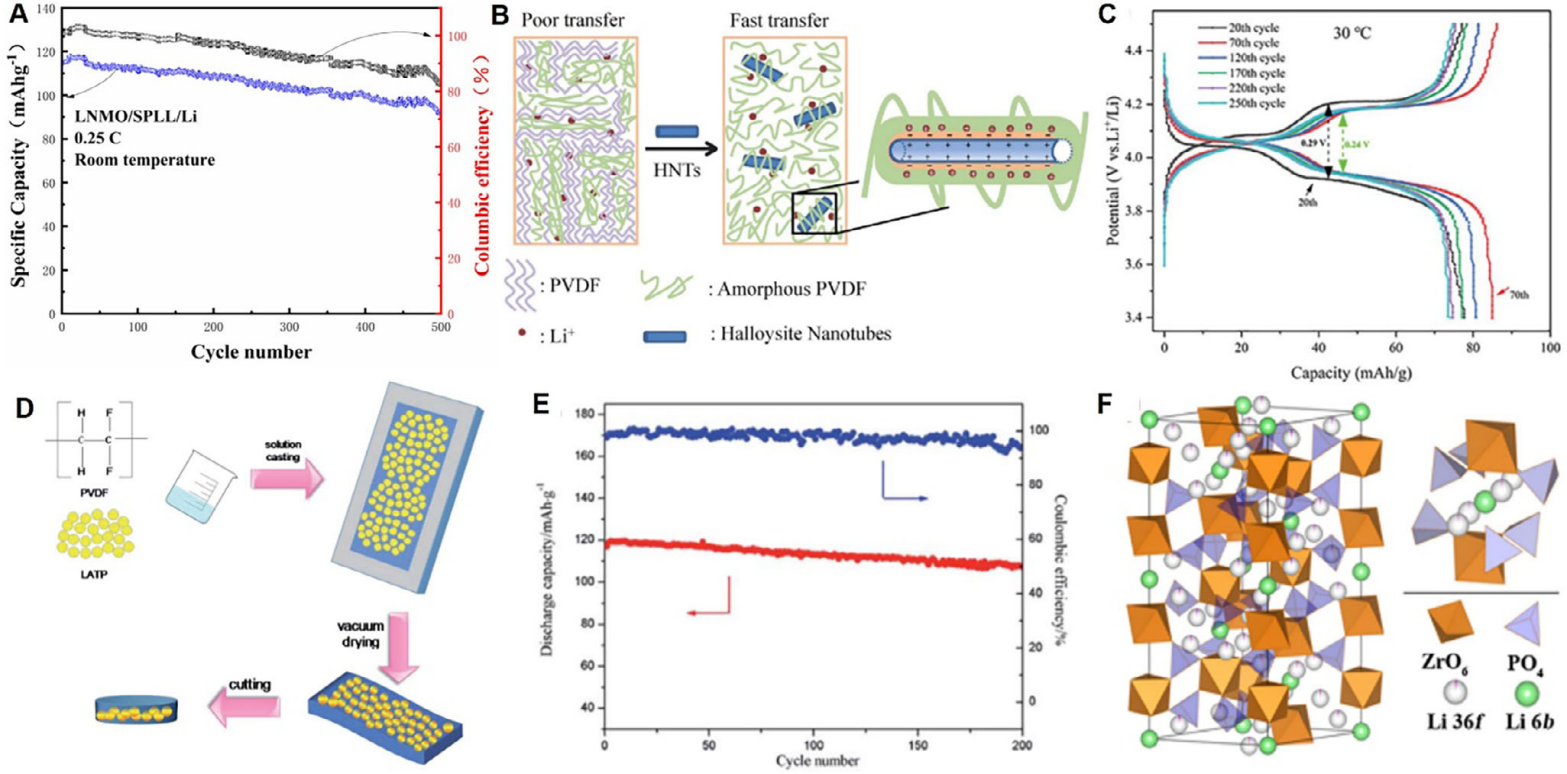
A) Cycling electrochemical performance of LNMO cathodes and three‐matrix solid electrolyte under 0.25C. Reproduced with permission.^[^ ^425^ ^]^ Copyright 2022, MDPI. B) Schematic illustration of the mechanism of halloysite nanotubes and PVDF composite electrolyte in enhancing the ionic conductivity. C) Typical charge‐discharge curve of LMO//HNTs‐PVDF//Li batteries under 1C. Reproduced with permission.^[^ ^459^ ^]^ Copyright 2018, The Royal Society of Chemistry. D) Schematic of the preparation of the composite PVDF‐LATP electrolyte. E) Cycling electrochemical performance of LMO//PVDF‐LATP//Li batteries under 0.2C. Reproduced with permission.^[^ ^462^ ^]^ Copyright 2018, the Royal Society of Chemistry. F) Crystal structure of Y‐doped LZP in PVDF‐HFP composite electrolyte. Reproduced with permission.^[^ ^468^ ^]^ Copyright 2024, American Chemical Society.

Active fillers are distributed into a wide range of lithium‐ion‐containing conducting materials: oxide‐based electrolytes (LLZO, LATP, LAGP, LLTO, etc.), sulfide‐based electrolytes (LGPS, LPSCl, etc.), halide‐based electrolytes (LYC, LIC, etc.), and other ceramic materials (LiPON, Li_3_N, etc.).^[^ ^419^, ^420^, ^421^, ^422^, ^423^, ^424^, ^425^ ^]^ Beyond exhibiting the same functions as inactive fillers, the active fillers also provide increased lithium‐ion transporting pathways to escalate the efficiency of ionic transportation. For example, Liang et al.^[^ ^424^ ^]^ developed a polysulfone (PES)‐based composite solid electrolyte with LATO as an active filler that could achieve 5.37 × 10^−4^ S cm^−1^ and expand the electrochemical stability window from 3 V to 5.21 V at ambient temperature. The ASSLIBs with LNMO as the cathode delivered an initial discharge capacity of 120 mAh g^−1^ at 0.1C at room temperature and stable operation with a Coulombic efficiency of 97% for 50 cycles. In their later work, another composite solid electrolyte was developed, which was composed of three polymer electrolytes (PES‐PVC‐PVDF), LiBF_4_ as Li salt, and LATZP and SiO_2_ as active fillers.^[^ ^425^ ^]^ The ASSLIBs utilizing LNMO/Li electrodes delivered an initial discharge capacity of 122 mAh g^−1^ at 0.1C at room temperature and excellent capacity retention for 500 cycles with 82.5% capacity retention at 0.25C, as shown in **Figure** **9**A.

Profiting from the advantages of both polymer electrolyte host and oxide electrolyte filler, solid composite electrolytes have become an excellent candidate as a promising electrolyte in ASSLIBs. It is worth mentioning that when pairing LMO, the solid composite polymer electrolyte could sufficiently tackle the severe Mn^2+^ dissolution challenge in liquid batteries at relatively higher operating temperatures (>55 °C).^[^ ^426^, ^427^ ^]^ Feng's group^[^ ^428^ ^]^ reported a composite solid‐state electrolyte of PVDF/LLZTO with a wide electrochemical stability window and excellent mechanical strength. At room temperature, the ASSLIBs with LMO cathodes delivered an initial discharge capacity of 67 mAh g^−1^ and demonstrated an excellent and stable cyclability with a capacity retention of 88% after 100 cycles. When operating at high 80 °C, the ASSLIBs could still deliver an initial discharge capacity of 80 mAh g^−1^ and capacity retention of 78.8% after 50 cycles. In stark contrast, the liquid battery only operated for 1 cycle due to continuous reactions between LMO and liquid electrolyte, which led to rapid material degradation.

#### 3.3.1 Introducing Inert Fillers

To promote room‐temperature ionic conductivity of the composite solid polymer electrolyte through the introduction of inert fillers, two widely recognized mechanisms include a) ceramic fillers reducing the crystalline degree of the host polymer matrix and facilitating the segmental motion, and b) the efficient Lewis acid‐base interactions between the polymer electrolyte and the ceramic fillers, accelerating the Li‐salt dissociation.^[^ ^429^, ^430^, ^431^ ^]^

To elevate the ionic conductivity of polymer solid‐state electrolytes, it is crucial to address the root challenge by suppressing their crystallinity. Introducing inorganic additives is carried out to overcome the challenges of poor ionic conductivity due to the high crystallinity degree in the PEO‐based solid polymer electrolyte.^[^ ^409^, ^432^, ^433^, ^434^ ^]^ Incorporating micro‐ or nano‐sized additives could efficiently reduce the ion‐ion interactions and the ion pair formations to achieve the effectiveness of suppressing the crystallinity of the polymer solid‐state electrolytes.^[^ ^435^ ^]^ The employment of nano‐sized inert fillers and their corresponding effectiveness in elevating the ionic conductivity in composite solid electrolytes has been widely reported.^[^ ^210^, ^390^ ^]^ Scrosati et al.^[^ ^436^ ^]^ introduced nano‐scaled ceramic powders, TiO_2_ (13 nm) or Al_2_O_3_ (5.8 nm), into PEO‐LiClO_4_‐based polymer electrolyte to promote low‐temperature ionic conductivity and enhance mechanical stability. Instead of introducing the fillers at room temperature, this work innovatively introduced the inert fillers at 80 °C. The nano‐scaled ceramic powders not only possessed a large surface area but also could sufficiently freeze the crystalline and suppress the recrystallization of the polymer matrix. Consequently, in the following process of cooling down to ambient temperature, the crystallinity of the PEO matrix was sufficiently suppressed, and the ionic conductivity of PEO‐based solid polymer electrolyte with 10 wt% TiO_2_ was elevated from 10^−8^ S cm^−1^ to 10^−5^ S cm^−1^ at 30 °C. Introducing monodispersed 12 nm nanospheres of SiO_2_ as ceramic fillers into PEO‐based composite solid polymer electrolyte was reported by Cui et al.^[^ ^409^ ^]^ The SiO_2_ nanoparticles were developed via an in‐situ controllable‐growth synthesis method instead of the conventional mechanical mixing method. This innovative method facilitated the improved and uniform SiO_2_ distribution and advanced surface area. As a result, the crystallinity of the PEO matrix was profoundly reduced, and the LiClO_4_ dissociation was sufficiently amplified. The composite solid polymer electrolyte achieved ionic conductivity of 4.4 × 10^−5^ S cm^−1^ at 30 °C and 1.2 × 10^−3^ S cm^−1^ at 60 °C. Moreover, the CV test confirmed that the electrochemical stability window was expanded to 5.5 V, demonstrating the potential to couple with high‐voltage cathodes such as LMO and LNMO. Notably, the interconnections among the nanoparticles were weak, which made it highly important to introduce a sufficient amount of additives to achieve the ionic conductivity enhancement. Meanwhile, excessive nanoparticles eventually resulted in increased ion‐ion interactions and sluggish ion transportation within polymer solid‐state electrolytes.^[^ ^415^ ^]^

Ruan's group^[^ ^437^ ^]^ reported a novel additive, TiO_2_‐based nanoparticle, that could significantly improve the ionic conductivity of PEO‐based polymer electrolytes. Incorporating the advantages of possessing high‐branched cavities and rich functional termination groups as a bridge for ion transportation,^[^ ^438^, ^439^ ^]^ the hyper‐branched poly(amine‐ester) (HBPAE) polymer electrolyte was grafted onto nano‐TiO_2_ by in situ synthesis. The optimized HBPAE‐grafted nano TiO_2_ with a concentration of 15 wt.% exhibited ionic conductivity of 3.2 mS cm^−1^ at room temperature, which was almost 10 times higher than a conventional PEO‐based solid polymer electrolyte. In the LIBs with LMO as cathode and LiTi_2_(PO_4_)_3_ as anode, the greatly improved electrochemical performance achieved an initial discharge capacity of 78 mAh g^−1^, which was almost double that of the pure PEO‐based electrolyte LIBs with a capacity of 42 mAh g^−1^.

The strong Lewis acid‐base interactions between the inert fillers and the polymer matrix could accelerate the Li‐salt dissociation. Therefore, while the anions of the Li‐salt were attached to the surface of the filler, the elevated concentration of Li‐ion at the interface facilitated improved ionic conductivity.^[^ ^440^, ^441^ ^]^ An et al.^[^ ^442^ ^]^ introduced Mg^2+^ and Al^3+^ into PEO‐based solid polymer composite electrolytes to facilitate Lewis‐acid coordinated polymer in enhancing the low ionic conductivity and expanding the electrochemical stability window. Notably, the Al^3+^ demonstrated the highest potential of improving the ionic conductivity, while the Mg^2+^ exhibited the capability of achieving a wide electrochemical stability window. Therefore, employing both Mg(ClO_4_)_2_ and Al(ClO_4_)_3_ to incorporate the synergistic effects of both the Al^3+^ and Mg^2+^ simultaneously. The Mg^2+^ and Al^3+^ coordinated environment in electrolytes facilitated the dissociation of the LiTFSI, reinforced the Li‐ion migration rates, and coordinated and protected EO chains from oxidation. Consequently, the enhanced PEO‐based composite solid electrolyte achieved an elevated ionic conductivity of 0.51 mS cm^−1^ at room temperature and integrated the electrochemical stability window to 4.8 V.

#### 3.3.2 Incorporating Inorganic Active Fillers

Incorporating high‐efficiency and low‐cost active filler(s) into polymer solid‐state electrolytes is one of the commonly utilized strategies to enhance the ionic conductivity of composite solid electrolytes. Wang et al.^[^ ^443^ ^]^ employed LAGP‐PEO(LiTFSI) as the composite solid electrolyte to mitigate the challenges of poor interface contact between rigid LAGP and the cathode and the low room‐temperature ionic conductivity of PEO‐based polymer electrolyte. This work innovatively applied LMO and LiFePO_4_ (LFP) as the composite cathodes to reduce the operating voltage.^[^ ^444^ ^]^ EIS tests indicated the stability of the LMO/LFP interface and suggested the suppression of the Mn^2+^ dissolution and Jahn‐Teller effect. The ASSLIBs delivered an initial discharge capacity of 169.6 mAh g^−1^ and maintained nearly 100% capacity retention after 80 cycles. Goodenough et al.^[^ ^445^ ^]^ investigated the composite polymer solid electrolytes by employing garnet ceramic Li_6.4_La_3_Zr_1.4_Ta_0.6_O_12_ (LLZTO) as an active filler with PEO‐LiTFSI‐based electrolytes. The LLZTO additive enhanced the ionic conductivity of composite electrolytes by both reducing the crystalline degree of the polymer matrix and providing alternative Li‐ion pathways. The concepts of “ceramic‐in‐polymer” and “polymer‐in‐ceramic” were identified by the content of LLZTO (0–80 wt.%) within the composite electrolytes. Optimized ionic conductivity with 10 wt.% of LLZTO was achieved around ≈10^−4^ S cm^−1^ at 55 °C. Wang et al.^[^ ^446^ ^]^ for the first time, experimentally demonstrated the effectiveness of introducing a fast ion conductor LiAlSiO_4_ (LASO) into PEO‐based polymer solid‐state electrolytes in improving the ionic conductivity of composite polymer solid‐state electrolytes at relatively low temperatures (< 65 °C) and expanding the electrochemical stability window from 4.55 V to 4.90 V, simultaneously. Structurally, within the LASO, Si and Al share oxygen atoms in the tetrahedral units, which benefits lithium ions' migration between the gap positions in LASO.^[^ ^447^ ^]^ The optimized composite solid polymer electrolyte achieved an excellent ionic conductivity of 1.62 × 10^−4^ S cm^−1^ at 40 °C and 4.68 × 10^−4^ S cm^−1^ at 60 °C. The ASSLIBs with LMO as cathode and PEO‐LiTFSI‐LASO as SSE demonstrated discharge capacity of 85 mAh g^−1^ at 0.2C and 56 mAh g^−1^ at 0.5C under 30 °C, and steady cycling performance with close to 100% coulombic efficiency for 50 cycles. These results further confirmed the capability of coupling composite polymer solid‐state electrolytes with Mn‐based cathodes.

The effectiveness of incorporating the nanoparticle fillers in suppressing the crystallization of the polymer matrix and elevating the ionic conductivity was widely studied and confirmed.^[^ ^448^, ^449^, ^450^ ^]^ Alternatively, introducing the nanowire fillers facilitates continuous and facile Li‐ion migration pathways to overcome the junctions between nanoparticle fillers. Cui et al.^[^ ^213^ ^]^ for the first time reported the application of introducing ceramic electrolyte Li_0.33_La_0.557_TiO_3_ (LLTO) nanowire into PAN‐LiClO_4_‐based composite polymer solid electrolyte to enhance the ionic conductivity and the electrochemical stability. Consequently, the composite polymer solid electrolyte incorporated with 15 wt.% LLTO nanowires demonstrated the greatest ambient temperature ionic conductivity of 2.4 × 10^−4^ S cm^−1^, which was 1–2 orders of magnitude higher than the one with LLTO nanoparticle (∼10^−6^ S cm^−1^), and three orders of magnitude improved than filler‐free PAN‐LiClO_4_ (2.1 × 10^−7^ S cm^−1^). The enhancement of the ionic conductivity could be attributed to a) the Li‐ion mobility was accelerated by the interaction between Li‐salt and “─C≡N” terminal on PAN, b) the perovskite‐type LLTO was A‐site deficient, which exhibited enriched surface vacancies, enabling Li‐ion hopping from one vacancy to a nearby one, and c) the expanded surface area by nanowires, which facilitated 3D ion‐conducting pathways in the polymer matrix. In the second work, Cui's group modified the orientation of the nanowire LLTO additives from random‐oriented to organized‐aligned to further enhance the ionic conductivity of PAN‐LiClO_4_‐based composite polymer solid electrolyte.^[^ ^451^ ^]^ The well‐organized nanowires further reduced the crossing junctions during Li‐ion migrations and facilitated fast ion conduction. Incorporated with 3 wt.% aligned nanowire additives, the composite polymer solid electrolyte achieved ionic conductivity of 5.4 × 10^−6^ S cm^−1^ at 30 °C, which was one order of magnitude higher than the composite electrolyte without fillers (3.62 × 10^−7^ S cm^−1^). Moreover, the 0° orientation alignments enabled the calculation of the nanowires' surface conductivity. The calculated interface conductivity reached 1.26 × 10^−2^ S cm^−1^, which was competitive with the liquid electrolytes, demonstrating the promising potential in the further design of composite polymer solid electrolyte‐based ASSLIBs. Yang et al.^[^ ^452^ ^]^ systematically tested the effectiveness of different cubic structure Li_7_La_3_Zr_2_O_12_ (LLZO)‐based nano‐sized additives in elevating the ionic conductivity of PAN (polyacrylonitrile)‐LiClO_4_‐based composite polymer solid electrolytes. In this study, the effectiveness of the doped and undoped LLZO nanowires, LLZO nanoparticles, and Al_2_O_3_ nanowires was investigated. With 5 wt.% of Ta‐doped LLZO (Ta‐LLZO) nanowire additives, the composite polymer solid‐state electrolyte demonstrated the greatest room‐temperature ionic conductivity of 1.5 × 10^−4^ S cm^−1^, which was slightly higher than the composite polymer solid electrolytes with nanowire additives of undoped‐LLZO (1.31 × 10^−4^ S cm^−1^) and Al‐LLZO (1.27 × 10^−4^ S cm^−1^). The three orders of magnitude increment of ionic conductivity over additive‐free PAN‐LiClO_4_ (10^−7^ S cm^−1^ at room temperature) was attributed to the nanowire morphology and the excellent ionic conductivity and high dielectric constant (40–60) of the active filler.^[^ ^453^, ^454^ ^]^ In addition, solid‐state Li nuclear magnetic resonance (NMR) studies revealed that Li‐ion exhibited preferential migration pathways at the interface of LLZO and PAN matrix than the individual PAN polymer amorphous phase or the LLZO nanowire phase. This observed behavior was different from that reported in the PEO/Al‐LLZO study,^[^ ^455^ ^]^ which originated from the distinct functioning mechanisms of the additives in PEO and PAN. As previously discussed, in PEO, the active filler could sufficiently reduce the degree of crystallinity, in elevating the ionic conductivity. Meanwhile, the crystallinity of PAN was not significantly affected by the active ceramic filler.^[^ ^213^, ^456^ ^]^ The XRD results from this work further confirmed that the PAN was in an amorphous state that was independent of the incorporation of active filler.

The above‐mentioned strategies that employ the nanoparticles and nanowires in integrating the electrochemical performance of composite polymer solid‐state electrolytes have not been applied in the Mn‐based ASSLIBs. However, the advanced strategies and the outstanding effectiveness demonstrate the potential of coupling with Mn‐based cathodes and shed light on the future design of ASSLIBs.

Attracted by the intrinsic properties of high specific surface area, negatively charged surface, and environmentally friendly nature, the nano‐structured oxide‐electrolytes have become an ideal candidate as an active filler for composite polymer solid electrolytes, especially those that contribute to Li‐ion transportation.^[^ ^457^ ^]^ Hu et al.^[^ ^458^ ^]^ reported for the first time to incorporate 3D garnet nanofiber Li_6.4_La_3_Zr_2_Al_0.2_O_12_ (LLZAO) with PEO‐based composite solid polymer electrolyte to enhance the ionic conductivity and mechanical strength. The 3D structure facilitated continuous Li‐ion migration pathways within the polymer PEO matrix. Consequently, the composite polymer solid electrolyte demonstrated an improved room‐temperature ionic conductivity of 2.5 × 10^−4^ S cm^−1^. Chen's group introduced a PVDF‐based solid‐state composite electrolyte with halloysite nanotubes (Al_2_Si_2_O_5_(OH)_4_•nH_2_O).^[^ ^459^ ^]^ The ionic conductivity of the composite polymer solid‐state electrolyte was elevated from ≈10^−5^ S cm^−1^ to 3.5 × 10^−4^ S cm^−1^ at 30 °C. Incorporating nanotube fillers enabled maintaining PVDF in amorphous structures and suppressing the formation of crystalline regions, hence improving the ionic conductivity of the composite polymer solid‐state electrolytes (Figure 9B). The ASSLIBs with the LMO cathode and Li anode delivered an initial discharge capacity of 71.9 mAh g^−1^ and an increased discharge capacity of 95.2 mAh g^−1^ at the 23rd cycle. The increment of the reversible capacity originated from the stable interface between the halloysite nanotube and PVDF that gradually formed during cycling. The ASSLIBs exhibited excellent cycling stability for more than 250 cycles, as shown in Figure 9C, and an excellent rate capability of delivering a discharge capacity of 74.4 mAh g^−1^ at 3.5C.

PVDF‐based polymer electrolytes exhibit excellent salt dissolution capability and a facile free‐standing fabrication procedure, which has been intensively studied to improve ionic conductivity to meet expectations.^[^ ^460^, ^461^ ^]^ Mao's group^[^ ^462^ ^]^ reported PVDF and ceramic NASICON‐type—Li_1.3_Al_0.3_Ti_1.7_(PO_4_)_3_ (LATP)‐based composite solid electrolytes to enhance the ionic conductivity as shown in Figure 9D. The optimized composition with improved Li‐salt concentration delivered ionic conductivity of 1.58 × 10^−4^ S cm^−1^ at room temperature. The challenge of poor interfacial contact between LATP and the cathode was addressed.^[^ ^463^, ^464^, ^465^ ^]^ The ASSLIBs with LMO as cathode delivered an initial discharge capacity of 117 mAh g^−1^ at 0.2C and retained capacity of 107.4 mAh g^−1^ after 200 cycles with almost 100% Coulombic efficiency, indicating excellent long‐cycling stability (Figure 9E).

Kumar et al.^[^ ^466^ ^]^ reported a composite polymer solid electrolyte comprised of P(VDF‐HFP) as a polymer matrix, LiTFSI as Li‐salt, and 15 wt.% of LiZr_1.5_Sn_0.5_(PO_4_)_3_ (LZSP) as an active filler. Isovalent Sn^4+^ substitution into NASICON‐type ceramic electrolyte was capable of enhancing the facile Li‐ions migration pathways within the ASSLIBs.^[^ ^460^, ^467^ ^]^ The composite solid electrolyte achieved an ionic conductivity of 2.87 × 10^−4^ S cm^−1^ at room temperature. The active additive modification strategy provided increased Li‐ion transportation channels while strengthening the mechanical properties of the polymer solid‐state electrolytes. As a result, the ASSLIBs with LMO as the cathode demonstrated superior room‐temperature electrochemical performance with an initial discharge capacity of 105 mAh g^−1^ and 93% capacity retention after 100 cycles. Very recently, following the same principle, Kumar et al.^[^ ^468^ ^]^ reported another P(VDF‐HFP)‐based composite polymer solid electrolyte with Y‐doped ceramic NASICON‐type LiZr_2_(PO_4_)_3_ electrolytes, as shown in Figure 9F. Different from Sn^4+^ isovalent substitution in the previous work, Y^3+^ aliovalent substitution Li_1.15_Y_0.15_Zr_1.85_(PO_4_)_3_ facilitated increased Li‐ion concentration and suppressed secondary phase formation.^[^ ^469^ ^]^ With 15 wt.% of Y‐LZP, the composite polymer solid electrolyte achieved an ionic conductivity of 5.78 × 10^−5^ S cm^−1^ at ambient temperature. The ASSLIBs with LMO cathode and Li anode delivered a discharge capacity of 104 mAh g^−1^ under 0.1C at room temperature.

Applying a buffer layer between the cathode and the solid polymer electrolyte could be considered another strategy to mitigate the challenge of a narrow electrochemical stability window. The buffer layer could effectively suppress the oxidation of the polymer solid‐state electrolytes and, therefore, improve the interfacial stability between the polymer solid‐state electrolytes and the Mn‐based cathodes. For example, Iwahori's group^[^ ^470^ ^]^ developed an artificial CEI by using Li_3_PO_4_ (LPO) as a buffer layer to overcome the challenge of electrochemical incompatibility of PEO‐based polymer solid‐state electrolytes (ethylene oxide co‐2‐(2‐methoxyethoxy)ethyl ether) with high‐voltage LNMO cathodes. An ≈100 nm thick LPO was introduced by electrostatic spray deposition onto the surface of the LNMO particles. At 60 °C, the all‐solid‐state thin film batteries (ASSTFBs) demonstrated competitive electrochemical performance to that of a liquid‐state battery over a voltage range of 3.0 V to 5.0 V with a discharge capacity over 100 mAh g^−1^. This observation implied that the effectiveness of artificial CEI in suppressing the oxidation degradation of polymer solid‐state electrolytes at high voltages. The two typical plateaus were observed at 4.7 V and 4.1 V, indicating the redox reactions of Ni and Mn, respectively. In stark contrast, the unmodified ASSTFBs only delivered one plateau under 3.5 V. Theoretically, during the charging process, the supply of O^2−^ from LNMO potentially led to and resulted in electrolyte oxidation and interfacial impedance increments.^[^ ^209^ ^]^ In this work, the ASSTFBs with LPO buffer layer demonstrated a stable high‐voltage charging plateau, indicating that the LPO could successfully prevent the supply of the LNMO cathode from oxidizing the electrolyte under high voltage. However, due to the high fabrication cost of ASSTFBs, large‐scale commercialization is still challenging. Therefore, in a later work, to further verify the feasibility and electrochemical performance of the composite electrolyte in the applications of ASSLIBs, Miyashiro et al.^[^ ^471^ ^]^ studied the ASSLIBs with a composite electrolyte that consisted of Li_3_PO_4_ and the solid polymer electrolyte which comprised of P(EO(ethylene oxide)/MEEGE(2‐(2‐methoxyethoxy) ethyl glycidyl ether)/AGE(allyl glycidyl ether)). As indicated in the previous work, Li_3_PO_4_ exhibited excellent anti‐oxidation stability and contributed to expanding the electrochemical stability window of polymer solid‐state electrolytes. However, in the first cycle, the ASSLIBs exhibited a typical 4.6 V voltage plateau with a significant amount of irreversible capacity (≈125 mAh g^−1^), which indicated the oxidation decomposition of the solid polymer electrolyte. This was further confirmed by less than 100% coulombic efficiency in the following second and third cycles. It was considered that the undesired electrolyte oxidation originated from heterogeneous powder mixing, resulting in direct contact between LNMO and the exposed solid polymer electrolyte. In a later work, the research group developed a sandwich structure by placing the ceramic Li_0.41_La_0.47_TiO_2.91_ (LLTO) SSEs as the buffer layer between the LMO cathode and a PEO‐based solid polymer electrolyte (ethylene oxide co‐2‐(2‐methoxyethoxy) ethyl ether) to mitigate the challenge of electrochemical incompatibility at the cathode interface.^[^ ^472^ ^]^ Benefiting from the excellent flexibility of the solid polymer electrolyte, close contact at the interface was successfully maintained. At 60 °C, the ASSLIBs exhibited high cycling reversibility and close to 60% capacity retention after 50 cycles. In stark contrast, the battery system with LMO/liquid electrolyte (LiPF_6_‐EC/DMC)/Li suffered severe Mn‐dissolution and capacity degradation with less than 20% capacity retention in less than 20 cycles.

In conclusion, the composite polymer solid‐state electrolytes have demonstrated the capability of integrating the merits of the composed solid‐state electrolytes. Ion transportation within polymer solid‐state electrolytes has been proven to be crucially determined by both the ionic motions and the host polymers' segmental motions.^[^ ^473^ ^]^ Benefitting from the inert or active fillers, the ionic conductivity, mechanical strength, and interfacial stability of the solid‐state composite electrolytes were significantly improved. It should still be noted that the room temperature ionic conductivity of composite electrolytes still needs to improve to ≈10^−3^ S cm^−1^ level to be competitive with other counterparts. Moreover, the preparation methods should also be further developed to avoid the employment of hazardous solvents and reduce the cost to realize practical applications in ASSLIBs.

### 3.4 Sulfide Solid‐State Electrolytes

#### 3.4.1 Introduction of Sulfide Solid‐State Electrolytes

Sulfide SSEs have garnered significant attention from researchers because of their outstanding room‐temperature ionic conductivity and robust mechanical properties, making them promising candidates for future applications in ASSLIBs. Typically, sulfide SSEs are comprised of Li_2_S and various sulfides (i.e., P_2_S_5_, GeS_2_, and SiS_2_).^[^ ^474^ ^]^ Based on the structural characteristics, sulfide SSEs are mainly categorized into three types: glass, glass–ceramic, and crystalline.

Glassy‐type sulfide SSEs possess outstanding advantages of isotropic ionic conductivity, low grain boundary resistance, facile fabrication methods, wide varieties of composition, etc.^[^ ^475^ ^]^ Notably, both oxide and sulfur elements belong to the chalcogens group; meanwhile, sulfur possesses a larger atomic radius. Consequently, when forming the sulfide SSEs such as Li_2_S‐SiS_2_ and 67Li_2_S‐33P_2_S_5_, the larger S^2−^ radius exhibited higher ion polarizabilities and a larger Li‐ion transmission channel.^[^ ^474^, ^476^, ^477^, ^478^ ^]^ Therefore, glassy sulfide electrolytes generally exhibit ionic conductivities ranging from 10^−3^ to 10^−5^ S cm^−1^ at room temperature, which surpass those of oxide glassy SSEs, which typically range from 10^−6^ to 10^−8^ S cm^−1^.

Structurally, glass‐ceramic sulfide SSEs, which possess superionic conductivity, are formed through precipitation of crystals from glass precursors. Compared with glassy‐type sulfide SSEs, the glass‐ceramic types exhibit enhanced ionic conductivity and reduced grain‐boundary resistance.^[^ ^479^ ^]^ Some of the glass‐ceramic sulfide SSEs could achieve high ionic conductivities of 10^−4^ to 10^−2^ S cm^−1^ at room temperature. The most commonly seen sulfide SSEs are in the chemistry formula of Li_7_P_3_S_11_, Li_3_PS_4_, and 80Li_2_S‐20P_2_S_5_.^[^ ^480^, ^481^, ^482^, ^483^ ^]^ Notably, in 2014, Tatsumisago et al.^[^ ^480^ ^]^ reported for the first time a glass‐ceramic conductor‐Li_2_S‐P_2_S_5_, possessing a superior high ionic conductivity of 1.7 × 10^−2^ S cm^−1^, which is competitive with its liquid counterparts.

The crystalline type of sulfide SSEs, which are known for their superior Li‐ion conductivities, has gained cumulative attention. Such a type of sulfide SSEs is generally categorized into two sub‐types, i.e., thio‐lithium super ion conductors (thio‐LiSICONs) and argyrodite‐type, with a general formula of Li_4‐x_Ge_1‐x_P_x_S_4_, and Li_6_PS_5_X (X = Cl, Br, and I), respectively.^[^ ^474^ ^]^ Notably, it was reported in 2011 that a thio‐LiSICON—Li_10_GeP_2_S_12_, featuring a novel 3‐D framework, demonstrated high lithium ionic conductivity of 12 mS cm^−1^ at room temperature.^[^ ^484^ ^]^ Both types of sulfide SSEs generally show superior room ionic conductivities. Iso‐ and/or aliovalent substitution into the host lattice was implemented to further enhance the ionic conductivity. A high conductivity and stable LiSICON Li_9.54_Si_1.74_P_1.44_S_11.7_Cl_0.3_, with a superior ionic conductivity of 25 mS cm^−1^ at room temperature,^[^ ^2^ ^]^ and the up‐to‐date, highest room temperature ionic conductivity of 32 mS cm^−1^ sulfide SSE, Li_9.54_(Si_0.6_Ge_0.4_)_1.74_P_1.44_S_11.1_Br_0.3_O_0.6_^[^ ^485^ ^]^ were reported by Kanno's group. Recently, high‐entropy types of halogen‐rich and multicationic substituted argyrodite sulfide SSEs Li_5.5_PS_4.5_Cl_x_Br_1.5‐x_ (0 < x < 1.5)^[^ ^486^ ^]^ and Li_6.66_[P_0.167_Si_0.5_Ge_0.167_Sb_0.167_]S_5_I^[^ ^487^ ^]^ with disordered cations, respectively, which exhibited enhanced electrochemical stability and ion mobility and increased ionic conductivities beyond 10 mS cm^−1^, were studied and reported.

Sulfide SSE‐based ASSLIBs demonstrate outstanding electrochemical performance, including high energy densities (> 800 Wh L^−1^),^[^ ^488^, ^489^ ^]^ great cycling stability with more than 70% capacity retention after long cycles (>500 cycles),^[^ ^488^, ^490^, ^491^, ^492^ ^]^ room‐temperature processability, and facile synthesis.^[^ ^493^, ^494^, ^495^ ^]^ Furthermore, the advantages of intrinsic densification capability and mechanical properties, i.e., lower Meyer hardness and elastic modulus,^[^ ^496^ ^]^ which leads to a closer contact interface between cathodes and SSEs. All these abovementioned advantages contribute to the great potential for sulfide SSEs in ASSLIB applications.

#### 3.4.2 Challenges in Coupling with Mn‐Based Spinel Cathodes

However, when sulfide SSEs were paired with Mn‐based spinel cathodes, the electrochemical performance of ASSLIBs still could not meet the satisfactory requirements. In 2010, Tatsumisago et al.,^[^ ^94^ ^]^ for the first time, introduced the spinel LMO as the cathode active material in the application of sulfide‐based ASSLIBs. The ASSLIBs with glass‐ceramic 80Li_2_S•20P_2_S_5_ (mol.%) as SSE and indium (In) as an anode, delivered a discharge capacity of 55 mAh g^−1^ in the first cycle and were associated with 10 mAh g^−1^ irreversible capacity. Such irreversible capacity potentially originated from the electronic property change of 1) the electronic conductivity of LMO and 2) the lithium diffusion coefficients at the end of the lithiation process. The ASSLIBs demonstrated long‐cycle stability that could maintain 45 mAh g^−1^ discharge capacity with 100% coulombic efficiency after 100 cycles. In addition, a severe Mn diffusion phenomenon of a Mn penetration of as far as 250 nm into sulfide SSE was observed at the CEI. Such Mn diffusion was suggested by the reactions between LMO and sulfide SSE, leading to the LMO decomposition at the interfacial layer. Zeier et al.^[^ ^497^ ^]^ demonstrated that the ASSLIBs with LMO as cathode and Li_6_PS_5_Cl (LPSCl) as electrolytes could barely cycle. The incompatibility between LMO and LPSCl resulted in severe electrolyte decomposition and led to an unstable interfacial layer. Yao et al.^[^ ^498^ ^]^ investigated the compatibility between LNMO and three different types of sulfide SSEs: 75% Li_2_S–24% P_2_S_5_–1% P_2_O_5_ (LPOS), LPSCl, and Li_10_GeP_2_S_12_ (LGPS). It was observed that the ASSLIBs with LPSCl SSE demonstrated a 4.0 V charge plateau with an initial charge capacity of ≈60 mAh g^−1^. In contrast, both LPOS‐ and LGPS‐based ASSLIBs only delivered a charge capacity of 20–30 mAh g^−1^ without the observation of characteristic plateaus and the capability of discharging. These observations further evidenced the incompatibility between these two sulfide SSEs and LNMO.

Therefore, to tackle the challenges between Mn‐based spinel cathode and sulfide SSEs to achieve an exceptional electrochemical performance, it is paramount to understand the root causes of the incompatibility.

##### Chemical Incompatibility

The chemical incompatibility between Mn‐based cathode and sulfide SSEs presents a significant challenge, because the chemical reactions occur spontaneously when these two materials are in contact, even at the open circuit voltage (OCV) during the battery fabrication process, i.e., preparing the composite cathode by mixing the CAM with SSEs, and continuously happen during the cycling. Due to the difference in chemical potential, there are significant difficulties in achieving a thermodynamic equilibrium state at the initial state between LMO/LNMO and sulfide SSEs within composite cathodes, as it may involve the formation of intermediate phases and/or equilibrium intermediate phases.^[^ ^195^, ^336^, ^346^ ^]^ Establishing this equilibrium state requires the occurrence of an interfacial chemical solid‐state reaction and rate‐determined interdiffusion or nucleation kinetics.^[^ ^196^, ^499^, ^500^ ^]^ In addition, the spontaneously formed cathode electrolyte interface (CEI) continuously grows as side reactions occur, which is experimentally observed as the high interfacial resistance.^[^ ^19^, ^195^ ^]^ This phenomenon was initially suggested by the Sasaki group as the “space charge effect”.^[^ ^342^ ^]^ Originating from the large chemical potential difference between the oxide cathode and the sulfide SSEs, the Li‐ions are spontaneously transported from the electrolyte to the cathode. Consequently, a large resistive lithium‐deficient layer, which was also called the “space charge layer”, formed on the electrolyte side of the cathode–electrolyte interface. Moreover, Li‐ions underwent a deintercalation advance of the cathode at the beginning of the first charging. As a result, an extra oxidation step before the cathode operating plateau was observed. Further studies revealed that instead of a Li‐deficient layer, it is the Li‐deficient grain boundary core that prevents lithium ions from transporting between grains, resulting in large interfacial impedance.^[^ ^341^, ^501^, ^502^ ^]^ This ionic passivating layer leads to the degradation of the capacity and deterioration of electrochemical performance in the ASSLIBs.

##### Electrochemical Instability

For most sulfide SSEs, such as LGPS, Li_4_GeS_4_, and LPSCl, the electrochemical stability windows are limited. For instance, the argyrodite‐type sulfide SSEs‐LPSCl possess a narrow electrochemical stability window of 1.7–2.3 V (vs Li/Li^+^). As a result, the component starts oxidizing when the applied voltage is above 2.3 V.^[^ ^19^ ^]^ Consequently, when coupled with Mn‐based cathodes, LMO, and LNMO, the oxidation reactions at high voltage (>4.3 V) during the battery cycling lead to the highly thermodynamically favorable transformation from S^2−^ into S.^[^ ^503^ ^]^ After the decomposition of the sulfide SSEs, the formed interphases and the by‐products at the interface contribute to increasing interfacial impedance and subsequently hinder the ionic and electronic conductivities and sluggish kinetics.^[^ ^420^ ^]^ For instance, LPSCl initially undergoes the decomposition of Li_4_PS_5_Cl and Li_11_PS_5_Cl, followed by further decomposition into more thermodynamically stable products of Li_3_PS_4_, S, and LiCl, and P, Li_2_S, and LiCl, respectively.^[^ ^195^, ^503^, ^504^, ^505^ ^]^

**Figure 10:**
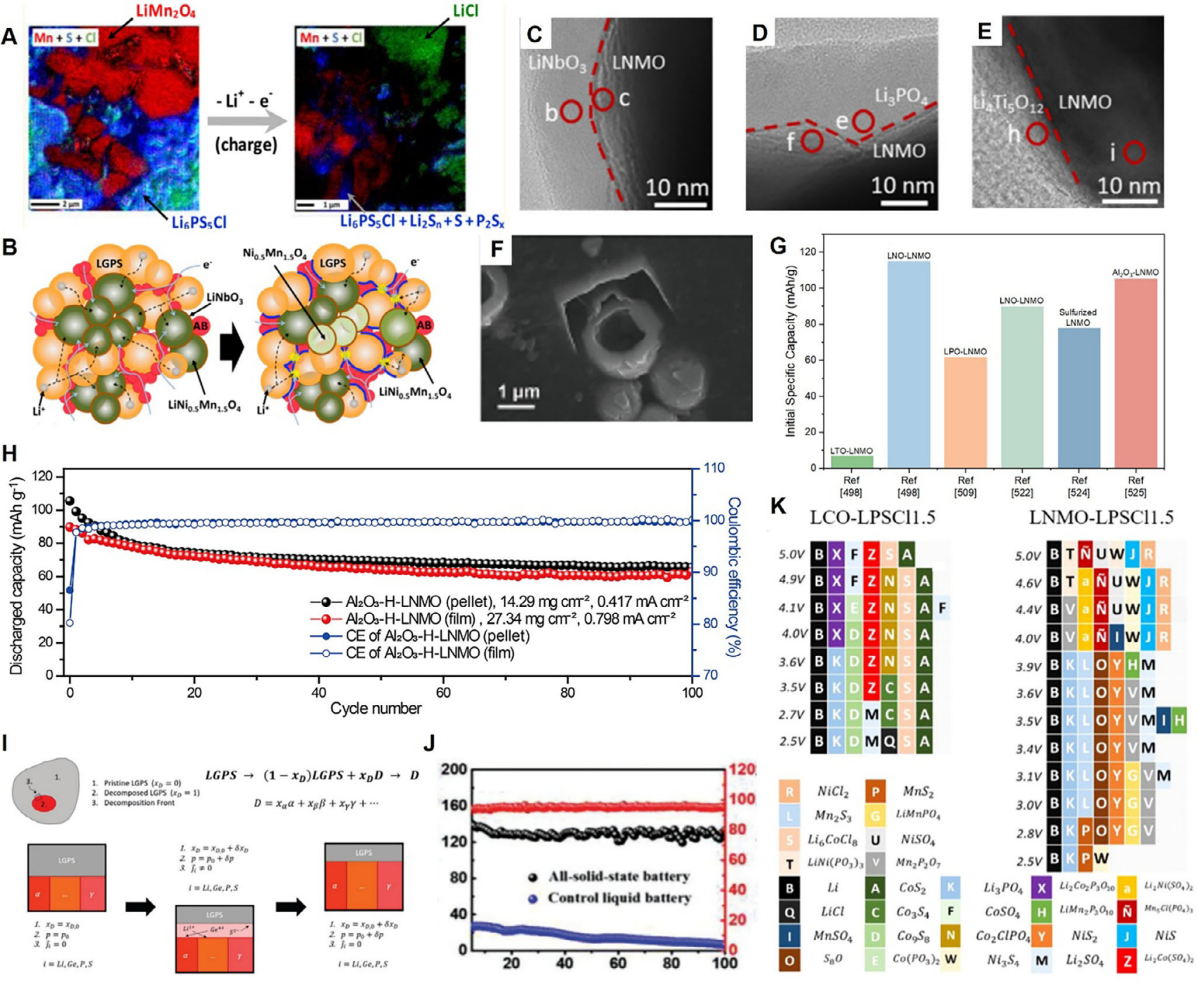
A) Scanning Auger microscopy (SAM) analysis of the pristine composite cathode and the composite cathode after 22 cycles. Reproduced with permission.^[^ ^506^ ^]^ Copyright 2017, American Chemical Society. B) Schematic of the interfacial reaction between LiNbO_3_@LiNi_0.5_Mn_1.5_O_4_ cathode and Li_10_GeP_2_S_12_ sulfide solid‐state electrolyte. Reproduced with permission.^[^ ^522^ ^]^ Copyright 2016, American Chemical Society. C–E) HRTEM images of 8 wt.% LiNbO_3_, 2 wt% Li_3_PO_4_, and 8 wt% Li_4_Ti_5_O_12_ coating LNMO, respectively. Reproduced with permission.^[^ ^498^ ^]^ Copyright 2020, American Chemical Society. F) SEM image of hollow LNMO particle cross‐section by FIB. G) Comparison of the initial discharge specific capacity between the references. H) Long cycling electrochemical performance of hollow LNMO in both pellet and film forms. Reproduced with permission.^[^ ^525^ ^]^ Copyright 2022, American Chemical Society. I) Schematic of the proposed mechanism of manipulating the decomposition propagation direction in maintaining kinetic stability. J) Long cycling electrochemical performance of LiNbO_3_@LNMO//LGPS//LTO under 0.3C. Reproduced with permission.^[^ ^529^ ^]^ Copyright 2020, Wiley. K) Computational calculations of decomposition products at the interfaces between LCO and LNMO cathodes and LPSCl_1.5_ between 2.5 and 5.0 V. Reproduced with permission.^[^ ^530^ ^]^ Copyright 2021, Elsevier.

Auvergniot et al.^[^ ^506^ ^]^ investigated the interface stability between the LMO and LPSCl to gain a more comprehensive understanding of the mechanism at the interfaces. Through the SEM and scanning Auger Microscopy (SAM) study of the LMO and LPSCl interface, the large reactivity‐derived side reaction products Li_2_S_n_, P_2_S_x_, and sulfites were observed, as shown in **Figure** **10**A, which were present even before electrochemical cycling. The ASSLIBs delivered an initial discharge capacity of 73 mAh g^−1^ and a Coulombic efficiency of 80% in the first ten cycles. In addition, the ASSLIBs retained a discharge capacity of 40 mAh g^−1^ after 22 cycles, indicating the instability of the interface and the oxidation of the sulfide SSEs. The heterogeneity of interfacial reactions was further confirmed via XPS analysis of various depths of mechanical etching from the surface, where a large amount of sulfur, polysulfides, and phosphates were observed at the 5 µm depth and disappeared at deeper depths.

#### 3.4.3 Strategies to Address the Challenges

One of the main root causes of rapid capacity fade for sulfide SSEs‐based ASSLIBs is the continuously growing interfacial resistance upon cycling.^[^ ^326^ ^]^ Therefore, to improve the capacity and electrochemical performance of the ASSLIBs, it is paramount to tackle the challenges at the interface between cathodes and sulfide SSEs. Various methods and strategies were researched and reported, such as coatings, dopants, tuning microstructures, and synthesizing methods, or a combination of those.^[^ ^507^, ^508^, ^509^ ^]^

##### Cathode Coatings

The CEI, which is spontaneously formed by the contact between the cathode and the SSE, is always arbitrary and uneven.^[^ ^510^ ^]^ Generally, this CEI could reach as thick as 50 nm at the interface, which significantly increases the impedance for ion transportation. When pairing with the cathodes with high operating voltage, sulfide SSEs, which exhibit limited electrochemical stability windows, become exacerbated during electrochemical cycling and hence, deteriorate the performance of ASSLIBs. Therefore, a better functioning artificial interface (coating) is highly demanded to overcome this challenge. It is worth noting that an excellent coating layer should exhibit a satisfactory and efficient passivating layer for Li‐ions conducting through the coating to ensure appreciable electrochemical performance. Meanwhile, the coating layer should also possess plausible electronic conductivity. Because the coating layer with exceeded electronic conductivity accelerates the electrochemical decomposition of the sulfide SSEs and leads to a rapid elevation in interfacial impedance. Meanwhile, a low electronic conductivity of the coating hinders the transport of electrons and hence the overall electrochemical performance.^[^ ^347^, ^510^ ^]^ The most commonly employed method is cathode surface coating to reinforce the cycling stability of the ASSLIBs. An extended working voltage window for the coating material is also beneficial so that the coating material can remain stable and chemically and electrochemically compatible with both cathode materials and the sulfide SSEs during the cycling operations. By controlling the thickness and composition of the coating materials, a desired stable artificial CEI is created to mitigate the interfacial chemical reactions and hence, enhance the cycling performance of the ASSLIBs.^[^ ^21^, ^22^, ^23^, ^24^, ^25^, ^26^, ^27^, ^28^, ^29^, ^30^, ^31^, ^32^, ^33^, ^34^, ^35^, ^36^, ^37^, ^38^, ^39^, ^40^, ^41^, ^42^, ^43^, ^44^, ^45^, ^46^, ^47^, ^48^, ^49^, ^50^, ^51^, ^52^, ^53^, ^54^, ^55^, ^56^, ^57^, ^58^, ^59^, ^60^, ^61^, ^62^, ^63^, ^64^, ^65^, ^66^, ^67^, ^68^, ^69^, ^70^, ^71^, ^72^, ^73^, ^74^, ^75^, ^76^, ^77^, ^78^, ^79^, ^80^, ^81^, ^82^, ^83^, ^84^, ^85^, ^86^, ^87^, ^88^, ^89^, ^90^, ^91^, ^92^, ^93^, ^94^, ^95^, ^96^, ^97^, ^98^, ^99^, ^100^, ^101^, ^102^, ^103^, ^104^, ^105^, ^106^, ^107^, ^108^, ^109^, ^110^, ^111^, ^112^, ^113^ ^]^

Through computational screening and evaluation, Cedar et al.^[^ ^510^ ^]^ suggested several oxide materials with optimal properties as excellent coating material candidates, such as LiNbO_3_ (LNO), Li_2_ZrO_3_ (LZO), LiTaO_3_, and polyanionic oxide LiPO_3_, LiTi_2_(PO_4_)_3_, etc. The employment of the coating material Li_4_Ti_5_O_12_(LTO),^[^ ^94^, ^511^ ^]^ LNO,^[^ ^507^, ^508^ ^]^ and Li_3_PO_4_ (LPO)^[^ ^509^ ^]^ to suppress the undesired side reactions was also confirmed through experimental tests. It is worth noting that each of the coating materials exhibits unique advantages. LNO exhibits the advantages of high conductivity of 10^−5^–10^−6^ S cm^−1^ at an amorphous state.^[^ ^342^ ^]^ LPO demonstrates excellent chemical inertness and is capable of reducing the side reactions and stabilizing the interface.^[^ ^512^ ^]^ LTO as a coating material not only possesses a zero‐strain structure and maintains structural stability during the lithiation and delithiation process, but also contributes to reducing the space charge layer effects between sulfide SSEs and cathode active material.^[^ ^513^ ^]^ Mo et al.^[^ ^514^, ^515^ ^]^ delineated that the coating material should be thermodynamically stable at both charged and discharged states to alleviate the potential deleterious side reactions due to the significantly strong reactivity of the cathode materials. In this study, various types of coating materials, including lithium ternary oxides, binary oxides, polyanion oxides, and lithium ternary fluorides, were systematically investigated. It was confirmed that at the delithiated state, the typical high‐voltage cathode LNMO was identified as fundamentally unstable with lithium ternary oxides such as Li_3_PO_4_ and LiTaO_8_. This phenomenon was precisely aligned with the general trends found from this study, which showed that the stability of the cathode vitally relied on the lithium content of the coating materials. Namely, the delithiated cathodes, which manifested a low concentration of Li‐ion, exhibited deliberate Li‐ion extraction force from the contacting material, and led to thermodynamic instability. Concurrently, even though the lithiated cathodes demonstrated increased stability compared to the delithiated cathodes, since they possessed higher Li contents, it was still observed that the elevated instability occurred when in contact with the higher Li‐content contact materials. In addition, the common binary oxides such as Al_2_O_3_, SiO_2_, and ZrO_2_ with the scarcity of the Li content demonstrated reasonable stability with the cathodes without the capability of extracting the Li‐ion, and were also experimentally reaffirmed.^[^ ^516^, ^517^, ^518^ ^]^ Among the studied polyanion oxides, such as lithium borates, silicates, and phosphates, LPO was scrutinized as the most promising candidate since it possessed extraordinarily high oxidation limits and higher‐voltage stability with the delithiated cathodes than the others. Intriguingly, the lithium ternary fluorides such as LiPF_6_ and LiBF_4_ with high Li extent, exhibited profound stability to high‐voltage cathodes‐LNMO, even at a delithiated state. This study elucidated fundamental understanding and the mechanisms of probing the coating materials and provided invaluable insights into the future pragmatic approaches and rational designs of optimal ASSLIBs.

The origin of the chemical incompatibility‐derived large interfacial impedance was considered to be the existence of the space charge layer. This finding aligned with the studies of ASSLIBs with LiCoO_2_ (LCO) cathode and sulfide SSEs, where a large interfacial resistance was also observed.^[^ ^342^, ^506^ ^]^ Even though it was accepted that the unfavored side reactions persisted between LCO and sulfide SSEs, asserting that the LCO and LMO possess the same reactivity to the sulfide SSEs was challenging. Accordingly, Sasaki et al.^[^ ^508^ ^]^ investigated the effectiveness of employing LiNbO_3_ (LNO) as the cathode coating layer for LMO/Li_3.25_Ge_0.25_P_0.75_S_4_ (LGPS) ASSLIBs in suppressing the high interfacial resistance caused by the space charge layer effect. It was found that the interfacial resistance decreased with the increase in the thickness of the LNO coating. With a 20 nm coating, the interfacial resistance was significantly reduced by two orders of magnitude from the pristine state of 1 × 10^4^ Ω to 200 Ω. The space charge effect was also effectively suppressed since the direct contact between the cathodes and the sulfide SSEs was reduced at the interface layer. In addition, the thickness of the space charge layer was quantitatively calculated as 9.7 nm, which was comparable with other studies of 8.3 nm (LCO/LGPS).^[^ ^519^, ^520^ ^]^ Similarly, Kanno et al.^[^ ^521^, ^522^ ^]^ investigated the effectiveness of LNO coating on LNMO when coupled with LGPS in sulfide SSE‐based ASSLIBs. The optimized thickness of the LNO coating layer was 9.7 nm (3 wt.%). As a result, the ASSLIBs under 0.05C delivered an initial discharge capacity of 53 mAh g^−1^, significantly improved compared to the pristine ones that were barely discharged. The potential root cause of the low discharge capacity was the low conductivity of LNMO, which was 10^−6^ S cm^−1^ at room temperature.^[^ ^164^ ^]^ Therefore, an oxygen‐deficient LiNi_0.5_M_1.5_O_4‐δ_ powder by high‐temperature annealing was introduced to further investigate how the electronic conductivity would affect the intercalation property. The optimized annealing temperature, 800 °C, delivered a significantly higher electronic conductivity of 8.9 × 10^−4^ S cm^−1^ than the pristine 5.6 × 10^−6^ S cm^−1^. The ASSLIBs with LNO coating increased the discharge capacity to 80 mAh g^−1^, suggesting the Li‐ion intercalation was improved. In addition, the enlarged 3.4 V discharge plateau observed in the 800 °C annealed LiNi_0.5_M_1.5_O_4‐δ_ sample further confirmed the increased ratio of Mn^3+/^Mn^4+^ redox reactions, which originated from the oxygen deficiency and increased contents of Mn^3+^. Additionally, two strategies were also proposed to overcome the challenge of low electronic conductivity in improving the Li intercalation rates: 1) increase the electrode/electrolyte interface contact area by reducing the particle size, and 2) introduce conductive carbon additives to increase the surface electronic conductivity. In the second work,^[^ ^522^ ^]^ the research group further investigated the effectiveness of these two strategies. Increased the interfacial contact area by employing LNMO with an average particle size of 7.9 µm (compared to pristine of 9.5 µm), the ASSLIBs delivered a higher initial discharge capacity of 93 mAh g^−1^. However, the ASSLIBs still suffered rapid capacity fading with a discharge capacity of <60 mAh g^−1^ in the 10^th^ cycle. The largely developed interfacial resistance from 832 Ω (first cycle) to 2240 Ω reaffirmed the side reactions with the formation of electron‐insulating Li_2_S. When employing the conductive AB as an additive in the composite LNMO/LGPS/AB cathode, through constant current/constant voltage (CC/CV) charging mode, an initial discharge capacity of 89 mAh g^−1^ was achieved. Severe capacity fading was still observed and consequently, reduced to <40 mAh g^−1^ in the 10th cycle. CV tests of LNO and LGPS confirmed the electrochemical stability between 3.5 V and 5 V without observation of oxidation and reduction. In contrast, the AB/LGPS exhibited an unstable interface, which was evidenced by the observable oxidation current. Thus, the irreversible capacity loss originated from the continuous decomposition of LGPS at the LGPS/AB interface above 4.5 V, as shown in Figure 10B. Yao et al.^[^ ^498^ ^]^ reported that when pairing LNMO with LPSCl, an optimized LNO cathode surface coating with a thickness of 15 nm, as shown in Figure 10C, demonstrated the best electrochemical performance. The ASSLIBs delivered an initial charge and discharge capacity of 136 and 115 mAh g^−1^, respectively. Even with the LNO coating, a severe capacity decay was observed in the first 10 cycles, with a discharge capacity of 80 mAh g^−1^, while a stable cycling performance of close to 100% capacity retention was achieved in the following 10 cycles.

Employing LPO as a coating material was reported to be effective in improving the interface between the cathodes and the sulfide SSEs.^[^ ^523^ ^]^ Yao et al.^[^ ^498^ ^]^ investigated the effect of coating material LPO on LNMO via the wet‐chemical deposition method. As shown in Figure 10D, the ASSLIBs with an optimized thickness of 20 nm delivered an initial discharge capacity of 25 mAh g^−1^ and a reversible discharge capacity of 49.9 mAh g^−1^ after 20 cycles. Notably, the resistance of the electrode and the SSE layer was slightly increased from 56.8 to 80.7 Ω after cycling. The interfacial layer resistance between LNMO and LPSCl was observed as 2.3 × 10^4^ Ω, originating from the poor passivation at the interface. Tatsumisago et al.^[^ ^509^ ^]^ reported that the ASSLIBs, which were composed of the pristine LNMO and glass‐ceramic 80Li_2_S‐20P_2_S_5_, could barely perform electrochemical cycling due to a large over‐potential with a discharge capacity of <1 mAh g^−1^. In contrast, the ASSLIBs with a 100 nm thick amorphous LPO thin film, which was fabricated by pulsed laser deposition on LNMO particles, achieved an initial reversible capacity of 62 mAh g^−1^. The interfacial impedance was also significantly suppressed from 1.5 × 10^4^ Ω (LPO‐coated LNMO) to 350 Ω (bare LNMO), which further evidenced the importance of the stabilized interface in achieving an exceptional electrochemical performance.

Tatsumisago et al.^[^ ^511^ ^]^ investigated the effectiveness of applying an amorphous LTO coating on the LMO cathode when paired with sulfide SSE 80Li_2_S•20P_2_S_5_ (mol.%). The ASSLIBs delivered an initial discharge capacity of 72 mAh g^−1^, which was improved from previous non‐coated ASSLIBs of 53 mAh g^−1^.^[^ ^94^ ^]^ The interface was stabilized, which was confirmed by the reduced polarization effect and the decreased interfacial impedance after being fully charged to 4.6 V. Notably, the ASSLIBs still suffered capacity decay in the first 10 cycles from 72 mAh g^−1^ to ≈58 mAh g^−1^ but could retain as much as 96% discharge capacity for the following 40 cycles. LTO as a coating material for LMO demonstrated great functionalities in suppressing the formation of interfacial SCL and reducing the interfacial resistance. On the other hand, due to the incompatibility of high voltage, LTO is not a suitable coating material for LNMO. According to Yao et al.’s investigation and study,^[^ ^498^ ^]^ by tuning the weight percentage of the coating material (3–13%), the optimized coating (8 wt.%, 10 nm thickness, as shown in Figure 10E) could only deliver the charge and discharge capacity of 28 and 7 mAh g^−1^, respectively. The first‐principles calculations indicated that LTO is not thermodynamically stable when cycling higher than 3.7 V, which could accelerate the LTO oxidation or decomposition rate.^[^ ^503^ ^]^

The aforementioned coating materials demonstrated pronounced effectiveness in enhancing the long cycling stability of the ASSLIBs. Probing the adequate coating material through the rational design is essential and crucial. Therefore, to obtain a holistic understanding of the thermodynamic stability between the coating materials and the cathode materials during extended cycling is pivotal. Especially, with a concentration of side reactions at the interfaces and interphases leading to the formation of unfavorable byproducts that sluggish ion transport and increase the interfacial resistance.

##### Modification of Microstructures

Employing the surface coating of the Mn‐based spinel cathode demonstrated the efficiency of suppressing the side reactions and stabilizing the cathode and SSEs interface. Notably, beyond the surface coating strategy, tuning the microstructure of the cathode is another strategy to mitigate the interfacial resistance difficulties.

Wang et al.^[^ ^524^ ^]^ demonstrated a novel method of microstructure modification by sulfurizing the LNMO cathodes to tackle the interfacial incompatibility challenge with LPSCl. This novel modification possessed the advantages of facile manufacture and easy industrialization. The S doping led to a more stable metal‐O‐S bonding and could sufficiently suppress side reactions and stabilize the interfaces.^[^ ^422^ ^]^ The ASSLIBs consisted of LNMO‐S/LPSCl/LTO, with an optimized S doping ratio LiNi_0.5_Mn_1.5_O_4_‐S_0.15_, delivering an initial discharge capacity of ≈80 mAh g^−1^, which was almost three times higher than the pristine 25 mAh g^−1^. The optimum ASSLIBs exhibited 90.1% and 79.5% capacity retention after 10 cycles and 20 cycles, respectively, which was higher than the previous literature of 77.6%.^[^ ^522^ ^]^

Lee et al.^[^ ^525^ ^]^ developed a hollow structured LNMO, which held the advantages of reduced Li‐ion diffusion length, homogeneity of the de‐/lithiation process, and prevented internal stress accumulation. An optimized thickness of 1 nm amorphous Al_2_O_3_ was ALD‐coated on the surface of hollow‐structured LNMO, as shown in Figure 10F, to further enhance the interface stability. The ASSLIBs with the modified LNMO cathodes and LPSCl as sulfide SSEs delivered an initial discharge capacity of 105.5 mAh g^−1^ at 0.1C, which was improved from the uncoated hollow LNMO of 83.4 mAh g^−1^. The comparison of the initial discharge capacity of ASSLIBs with LNMO cathodes with various coatings and sulfide SSEs from the literature is shown in Figure 10G. In addition, the ASSLIBs could maintain a capacity retention of 62.1% after 100 cycles. The Al_2_O_3_ demonstrated the capability of effectively suppressing the side reactions, and the formed increased interfacial impedance passivation layer by the side products of LiCl, S, and P_2_S_x_. In addition, film‐type ASSLIBs with high active material loading of 27 mg cm^−2^ demonstrated an initial discharge capacity of 89.8 mAh g^−1^ and capacity retention of 70.1% after 100 cycles, as shown in Figure 10H.

Tatsumisago et al.^[^ ^526^ ^]^ reported a novel amorphous 80LiNi_0.5_Mn_1.5_O_4_•20X (mol% and X = Li_3_PO_4_, Li_2_SO_4_, and Li_3_BO_3_) lithium oxy‐acid salts, which were synthesized by a mechanochemical process.^[^ ^527^, ^528^ ^]^ 80LiNi_0.5_Mn_1.5_O_4_ • 20Li_2_SO_4_ exhibited the highest room temperature ionic conductivity of 4.3 × 10^−6^ S cm^−1^, outperforming the pristine cathode of 2.3 × 10^−7^ S cm^−1^. The initial discharging capacities were increased from 5 mAh g^−1^ for the pristine to 55, 60, and 150 mAh g^−1^ when employing Li_2_SO_4_, Li_3_BO_3,_ and Li_3_PO_4_ salts, respectively. The ASSLIBs with the Li_2_SO_4_ and Li_3_PO_4_ salts delivered larger discharging capacities than the charging capacity, which originated from the additional lithium insertion undertaken during the discharge process.

##### Expanding the Electrochemical Stability Window

Generally, the sulfide SSEs possess an intrinsic narrow electrochemical stability window, which results in undesired decompositions and side reactions, especially when paired with high‐voltage cathodes, that deteriorate the electrochemical performance of ASSLIBs. Li et al.^[^ ^529^, ^530^ ^]^ elaborated that the electrochemical stability of sulfide SSEs could be widened by manipulating the operational stacking pressure. Through a mechanical constriction strategy, the researchers innovatively stabilized the kinetics of the sulfide SSEs and expanded the electrochemical stability window from 2.1 V^[^ ^304^ ^]^ to approximately 10 V. Such modification not only sufficiently eliminated the ion diffusion‐isolating voids but also realized the capabilities of pairing with high‐voltage cathode LNMO with stable cycling. From simulation calculations, as shown in Figure 10I, when applying a ground state pressure of 4 GPa to LGPS SSEs, the free energy of LGPS was minimized and the decomposition was prevented, even above 5 V.^[^ ^529^ ^]^ In the electrochemical experimental tests, the ASSLIBs with LNMO/LTO as cathode and anode, respectively, demonstrated stable long cycling with the cut‐off voltage of 4.4 V (vs Li/Li^+^ or 2.9 V vs LTO). It was observed that the ASSLIBs could deliver an initial discharge capacity of 150 mAh g^−1^ and maintain capacity retention of 82% after 100 cycles under 0.3C, which significantly outperformed the control liquid battery (LiPF_6_ in EC/DMC as electrolyte), as shown in Figure 10J. In the second work,^[^ ^530^ ^]^ the group enlarged the electrochemical stability window of the Cl‐doped Li_7‐x_PS_6‐x_Cl_x_ (x = 0.5, 1,1.5, and 2, LPSClx) sulfide SSEs with the mechanical constriction strategy. From the calculation, LPSCl_x_ (x = 0.5, 1) could achieve an upper cut‐off voltage of 4.3 V; meanwhile, LPSCl_1.5_ and LPSCl_2_ were capable of reaching 4.7 V due to the intrinsic low reaction energy of the orthorhombic phase. LPSCl_1.5_ possessed higher stability with the Li anode than LPSCl_2_; therefore, the ASSLIBs encompassed graphene‐modified Li/LPSCl_1.5_/LNMO delivered an initial discharge capacity of 85.5mAh g^−1^ and a capacity retention of 84% after 50 cycles at 0.1C. Computational calculations for LPSCl_1.5_ with both LCO and LNMO cathodes were conducted to further understand the degradation mechanism and the corresponding decomposition products under different voltages, as shown in Figure 10K. From the XPS analysis, the contents of polysulfide (P_2_S_x_), transition metal polysulfide (TM_y_‐S_x_), phosphates (PO_x_), and metal phosphates were significantly increased and were aligned with the calculation results, which indicated that the severe decomposition at the LNMO/LPSCl_1.5_ interface could not be completely eliminated by mechanical constriction.

##### Elevating the Ionic Conductivity of the Composite Polymer/Sulfide Solid‐State Electrolytes

Beyond the direct implementation of sulfide SSEs in the applications of ASSLIBs, introducing sulfide SSEs to integrate the ionic conductivity of composite polymer solid electrolytes has also been investigated and studied. For instance, Li et al.^[^ ^531^ ^]^ reported a PEO‐based composite polymer solid electrolyte incorporating Li_10_SnP_2_S_12_ to enhance the ionic conductivity. The optimized ionic conductivity was achieved as 1.69 × 10^−4^ S cm^−1^ at 50 °C with 1% Li_10_SnP_2_S_12_. The employment of the Li_10_GeP_2_S_12_ (LGPS) in the composite SSEs was also widely reported. Zhao et al.^[^ ^532^ ^]^ reported a PEO‐based composite polymer solid electrolyte with LGPS as an additive. The composite electrolyte with 1% LGPS exhibited an ionic conductivity of 1.21 × 10^−3^ S cm^−1^ at 80 °C and 1.18 × 10^−5^ S cm^−1^ at 25 °C that was attributed to the reduced PEO polymer crystallinity and weakened interactions between Li‐ion and PEO chains. Zheng et al.^[^ ^533^ ^]^ studied the composite electrolyte, which consisted of LGPS, PEO, and LiTFSI, and the influence of LGPS/PEO content on Li‐ions at the interface, and hence the corresponding ionic conductivity. The optimized ratio of 70 wt.% LGPS delivered the greatest room‐temperature ionic conductivity of 0.22 mS cm^−1^. Chen et al.^[^ ^534^ ^]^ innovatively prepared a composite electrolyte, which collaborated in‐situ polymerized monomers poly(ethylene glycol) diglycidyl ether (PEGDE), LiTFSI, and sulfide electrolyte—Li_10_GeP_2_S_12_ (LGPS). By optimizing the concentration of LiTFSI (60 wt.%), the electrochemical stability window was expanded to 4.7 V. Moreover, the modification of introducing the 3D porous structure of implanting porogenic SeS_2_ into LGPS facilitated fast ion transportation pathways and elevated the ionic conductivity to 7.7 × 10^−4^ S cm^−1^, which was almost 200 times higher than P(PEGDE)‐60 (4.03 × 10^−6^ S cm^−1^). This strategy sheds light on the feasibility of applying sulfide electrolytes as active fillers to realize the possibilities of pairing composite polymer solid electrolytes with a high‐voltage cathode. Beyond the studies of LGPS as an active filler, a novel argyrodite‐type Li_6_PS_5_X sulfide SSE as an active filler to enhance the ionic conductivity of PEO‐based composite polymer solid electrolyte was reported by Li et al.^[^ ^535^ ^]^ Through a solid‐state reaction, a novel off‐stoichiometry sulfide SSE deviation‐Li_6.25_PS_5.25_Cl_0.75_ with increased Li and S contents demonstrated excellent room‐temperature ionic conductivity of 1.0 mS cm^−1^. With 1 wt.% Li_6.25_PS_5.25_Cl_0.75_, the composite polymer solid electrolyte achieved the optimum ionic conductivity of 1.2 × 10^−2^ mS cm^−1^ at room temperature and 1.4 mS cm^−1^ at 70 °C.

It is worth noting that the effectiveness and mechanism in enhancing the ionic conductivity of PEO‐based electrolytes, between sulfide electrolytes and oxide electrolytes (i.e., LGPS and LLZO), are different. Generally, the Li‐ions migrate within ceramic‐based composite polymer electrolytes via three pathways: intra‐polymer, intra‐ceramic, and polymer‐ceramic interface. In the composite electrolyte LLZO‐PEO (LiTFSI), due to the rigid mechanical properties of the LLZO, the ion‐conducting interfaces were difficult to form.^[^ ^452^, ^536^, ^537^ ^]^ Therefore, the Li‐ions could only transport through either PEO or percolated LLZO particles. In contrast, the sulfide electrolytes LGPS exhibited soft and robust mechanical strength and facilitated more conformal ion‐conducting interfaces, which offered extra Li‐ion migration pathways.

In conclusion, while sulfide‐based ASSLIBs offer advantages such as facile syntheses, low cost, and superior room temperature ionic conductivity, thorough evaluations of the chemical stability, electrochemical compatibility, and interfacial layer contact are essential during the rational design process. Coating is the most common method to tackle these challenges. The coating layer could sufficiently reduce cathode decomposition caused by side reactions and suppress the elemental mutual diffusion between the cathode and electrolyte. Notably, choosing proper coating materials, especially when coupled with high‐voltage Mn‐based cathode material, is nevertheless extremely essential. An adequate coating thickness is also paramount to deliver satisfactory electrochemical performance. A thin coating would not be sufficient to fully cover the cathode material and effectively reduce the interfacial resistance. Meanwhile, a thick coating acts as an extra resistance that hinders the Li‐ions transport.^[^ ^538^ ^]^ In addition, Deng et al.^[^ ^539^ ^]^ innovatively employed poly (3,4‐ethylenedioxythiophene) (PEDOT) semi‐conductive polymer thin film as a coating material to modify carbon nanotube (CNT) additives. Such modification sufficiently suppressed the decomposition of the sulfide SSEs (LGPS) induced by contact with CNTs and side reactions in the composite cathode and thus enhanced the interfacial stability and overall electrochemical performance. Microstructure modifications are alternative strategies to mitigate the interfacial challenges. For example, S‐doping could suppress side reactions between the cathode and the sulfide SSEs, while tuning the microstructure and constructing a shortened Li‐ion transportation path is beneficial in improving the electrochemical performance of ASSLIBs. The endeavors to mitigate the challenge of volume change have been scrutinized and dissected to accommodate the volume changes during cycling while maintaining mechanical integrity. Deng et al.^[^ ^540^ ^]^ identified that the origin of the cathode interfacial degradation was the residual lithium compounds. Because such residual compounds accelerated the oxidation of the studied sulfide SSEs (Li_5.5_PS_4.5_Cl_1.5_ and Li_10_GeP_2_S_12_) and led to cathode degradation and rapid capacity decay. The strategies of removing the impurities of by‐products were beneficial in maintaining a “cleaned” cathode surface and the integrity of the sulfide SSEs. Employing the sulfide SSEs as an active filler in the composite polymer SSE is a promising strategy to leverage the advantages of both electrolytes.^[^ ^541^ ^]^ Unfortunately, these strategies have not been implemented in the Mn‐based ASSLIBs to integrate the merits of the electrochemical performance. It is also worth mentioning that, even with the optimized modifications, the electrochemical performance of the sulfide‐based ASSLIBs remains unsatisfactory when compared to their liquid counterpart.^[^ ^542^, ^543^ ^]^ In addition, due to the poor air stability and limited wettability,^[^ ^524^, ^544^ ^]^ most sulfide SSEs must be synthesized and handled within the inert gas (argon)‐filled glove box. The prevailing methods of sulfide SSEs synthesis, such as ball‐milling (more than 10 h) followed by heat treatment, require high energy and time costs. The associated challenges of limited production rate and inhomogeneous products further increase the cost of the process.^[^ ^545^, ^546^ ^]^ Therefore, it is necessary and important to address these critical challenges and develop novel and efficient synthesis methods in accelerating the pace of large‐scale industrialization and cost‐effective manufacturing of sulfide SSEs.^[^ ^547^, ^548^ ^]^

### 3.5 Halide Solid‐State Electrolytes

#### 3.5.1 Introduction of Halide Solid‐State Electrolytes

Halide SSEs, another main type of inorganic SSE, exhibit exceptional advantages, including high ionic conductivity, excellent interfacial compatibility, and a facile synthesis process, thus possessing great potential in the applications of Mn‐based ASSLIBs.^[^ ^549^ ^]^

Currently, there are two types of halide SSEs: rock‐salt type and anti‐perovskite type. Most of the rock‐salt type halide SSEs consist of trivalent metal cations with the typical crystalline structure Li_3_MX_6_, where M = Sc, Y, Ln, and In, X = halogen group elements, i.e., F, Cl, Br, and I. Generally, the rock‐salt type halide SSEs exhibit high ionic conductivity at room temperature (∼10^−3^ S cm^−1^), for instance, Li_3‐x_M_1‐x_Zr_x_Cl_6_ (M = Y, Er, 1.4 × 10^−3^ S cm^−1^),^[^ ^550^, ^551^ ^]^ Li_3_InCl_6_ (0.84–2.04 × 10^−3^ S cm^−1^),^[^ ^552^, ^553^ ^]^ etc. The ion transportation mechanism can be considered trivalent metal M^3+^ dopes in LiX, where one M^3+^ replaces three Li^+^, creating two vacancies in the original octahedral interstitial structure and developing a vacancies‐rich environment for Li‐ion transportation.^[^ ^554^ ^]^

The anti‐perovskite type halide SSEs have a typical chemical formula Li_3‐x_OH_x_X, where X = Cl and Br, exhibit excellent electrochemical reduction stability and, thus, form a stable SEI with the lithium anode and are capable of long‐cycling with low interfacial resistance.^[^ ^555^ ^]^ However, the ionic conductivities of this anti‐perovskite type halide S‐SEs are not satisfying, for example, Li_5_(OH)_2_Cl_3_ (1.48 × 10^−7^ S cm^−1^),^[^ ^556^ ^]^ Li_2_OHCl (≈10^−5^ S cm^−1^),^[^ ^555^ ^]^ etc.

Due to the intrinsic properties of longer ionic bonds and larger ionic radius of the monovalent halogen anions, halide SSEs possess advantages of higher oxidation potential and polarizabilities compared to other polymer‐ and sulfide‐based SSEs. Through computational analysis, Wang et al.^[^ ^557^ ^]^ suggested that the halide SSEs possessed high stability against oxidation due to the halide anions,^[^ ^550^, ^558^ ^]^ and Li_3_InCl_6_ exhibited an electrochemical stability window of 2.38–4.26 V, while Li_3_YCl_6_ exhibited a range of 0.62–4.02 V. Experimental results further confirmed such high oxidation stability with stable cycles without observation of side reactions when coupled with bare LiCoO_2_ and LiNi_0.8_Co_0.1_Mn_0.1_O_2_ cathodes without the coating modifications.^[^ ^552^, ^553^, ^559^, ^560^, ^561^ ^]^ Zhao et al.^[^ ^562^ ^]^ analyzed the thermal effects on the interfacial stability. The results indicated that after being sintered at 320 °C, both LiMn_2_O_4_ and LiNi_0.5_Mn_1.5_O_4_ could remain stable with Li_3_InCl_6_; LiMn_2_O_4_ was also compatible with Li_2_OHCl, but LiNi_0.5_Mn_1.5_O_4_ decomposed to LiMnO_2_ and Li_x_NiO_2_.

#### 3.5.2 Strategies to Address the Challenges

**Figure 11:**
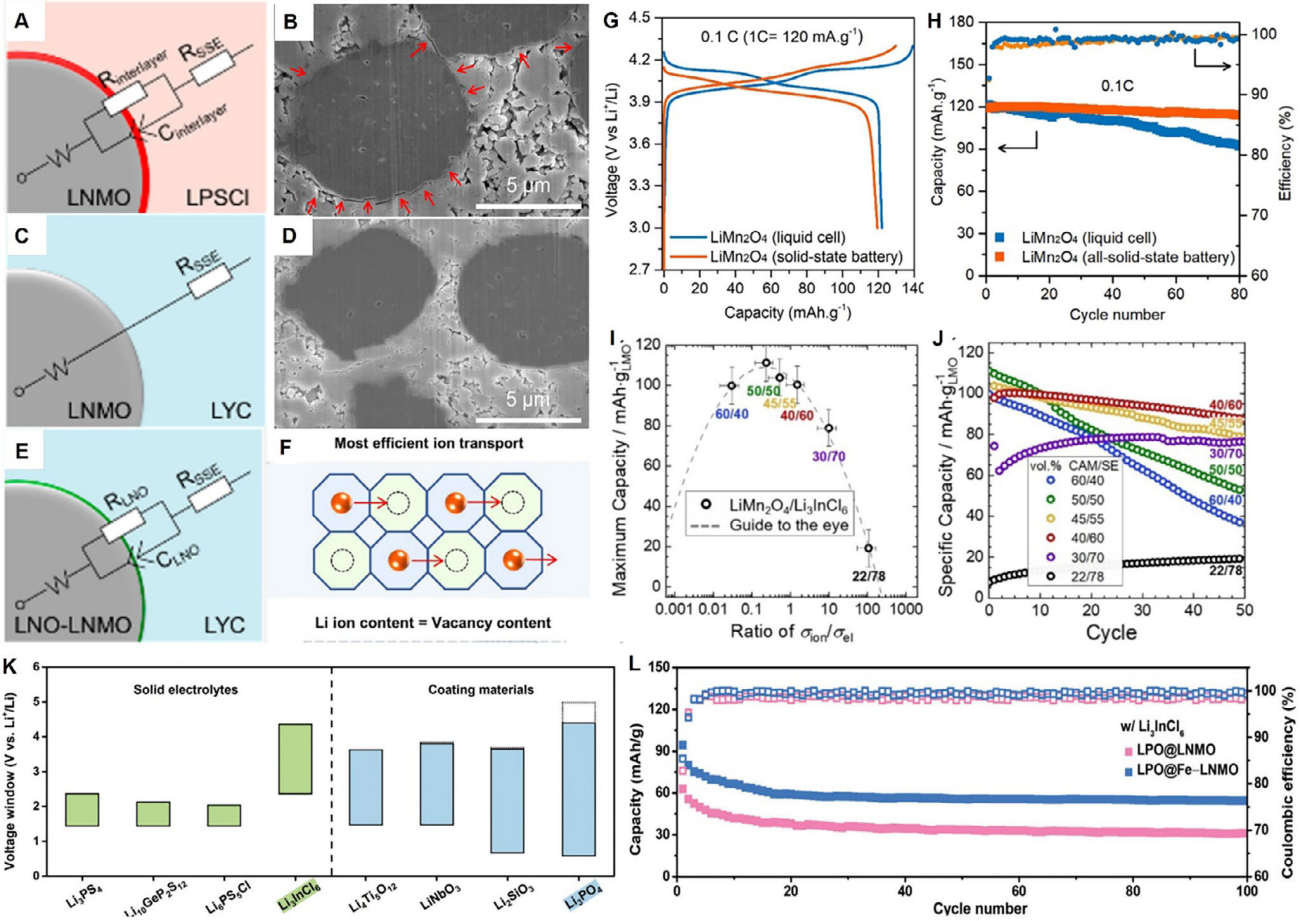
A) Schematic illustration of the interface between LNMO and LPSCl. B) SEM image of LNMO and LPSCl cross‐section before cycling. C) Schematic illustration of the interface between LNMO and LYC. D) SEM image of LNMO and LYC cross‐section before cycling. E) Schematic illustration of the interface between LNO‐coated LNMO and LYC. Reproduced with permission.^[^ ^563^ ^]^ Copyright 2022, American Chemical Society. F) Schematic of efficient ion transport with balanced ion and vacancy contents. G) Typical charge and discharge profile of LMO liquid and all‐solid‐state LIBs. H) Long cycling performance of LMO liquid and all‐solid‐state LIBs. Reproduced with permission.^[^ ^565^ ^]^ Copyright 2023, The Royal Society of Chemistry. I) Initial specific capacity at different LMO/LIC volumetric ratios and corresponding ratios of electron conductivity/ionic conductivity. J) Cycling behaviors for different volumetric ratios of LMO/LIC at 0.1C. Reproduced with permission.^[^ ^497^ ^]^ Copyright 2023, Wiley. K) Electrochemical stability window of solid electrolytes and coating materials. L) Long cycling capacity retention comparison of LPO‐coated LNMO and LPO‐coated Fe‐doped LNMO at 0.1C. Reproduced with permission.^[^ ^571^ ^]^ Copyright 2023, Wiley.

Meng et al.^[^ ^563^ ^]^ investigated the challenges of the ASSLIBs with LNMO coupled with sulfide SSE (LPSCl) and halide SSE (LYC). As illustrated in **Figure** **11**A,B, due to the chemical incompatibility between LNMO and LPSCl, side reactions and decomposition‐derived large interfacial impedance during charge and discharge eventually lead to poor electrochemical performance. The loss of interfacial contact and the formation of voids from the SEM image further evidenced the chemical incompatibility between LPSCl and LNMO at OCV. In contrast, LYC possessed excellent chemical compatibility with LNMO without emerging large interfacial impedance. The close interfacial contact without observations of decomposition was further confirmed via SEM, as shown in Figure 11C,D. However, the CV test demonstrated the oxidation peak of LYC at 4.3 V, which indicated the electrochemical incompatibility between LYC and LNMO of 4.7 V. Consequently, the ASSLIBs with bare LNMO and LYC only delivered an initial discharge capacity of 25.7 mAh g^−1^ and an initial Coulombic efficiency of 78.4%, without Ni^2+^ redox reaction above 4.5 V. The high polarization effect after 50 cycles further evidenced the oxidation of LYC at high voltage through the cycles. Therefore, amorphous LiNbO_3_ was employed as the cathode coating to mitigate the challenge of electrochemical incompatibility. Such an ionically conductive and higher oxidation‐stable layer could sufficiently suppress high voltage‐induced decomposition (Figure 11E). The initial discharge capacity and Coulombic efficiency were significantly improved to 91.0 mAh g^−1^ and 91.2%, respectively. The significantly reduced interfacial resistance throughout the cycling process proved the effectiveness of the coating in stabilizing the interface between the high‐voltage LNMO cathode and the halide SSEs.

In the halide SSEs, aliovalent substitution could facilitate elevated vacant sites for Li‐ion hopping within the structure, hence improving the ionic conductivity.^[^ ^564^ ^]^ To gain a better understanding of how the vacant site concentrations and ion contents influenced ion hopping and ionic conductivity, Sun et al.^[^ ^565^ ^]^ studied halide SSE of Li_3‐x_Lu_1‐x_Zr_x_Cl_6_ (0 < x <1) with Zr^4+^ substituted Lu^3+^ to elucidate the mechanism of the previously mentioned relationship. By manipulating the content of Zr^4+^, the optimized room temperature ionic conductivity was achieved as 1.5 mS cm^−1^ when Li‐ion concentrations were balanced with vacant site contents (i.e., x = 0.5), as shown in Figure 11F. The electrochemical cycling test of the ASSLIBs with bare LMO cathode and halide electrolyte Li_2.5_Lu_0.5_Zr_0.5_Cl_6_ under 50 MPa operating pressure and 0.1C current density delivered an initial capacity and Coulombic efficiency of 119.4 mAh g^−1^ and 91.8%, respectively. Notably, when compared with LMO cathode liquid LIBs, in the initial cycle, the liquid LIBs exhibited more typical Mn redox reaction plateaus, less polarization effects, and a slightly larger charge/discharge capacity. However, in 80 cycles of tests, under 0.1C, LMO‐based ASSLIBs demonstrated a capacity retention of 94.2% that significantly outperformed LIBs of ca.75% (Figure 11G,H). In addition, an excellent long cycling stability was demonstrated by over 1000 cycles at 0.3C and 150 MPa operation pressure with negligible capacity decay. This originated from the excellent chemical and electrochemical compatibility and interfacial stability between the LMO spinel cathode and the halide SSEs.

Through the studies of LMO paired with LPSCl and LIC, Zeier et al.^[^ ^497^ ^]^ emphasized the importance of balancing the cathodes' partial ionic and electronic transportation parameters in optimizing the electrochemical performance of ASSLIBs. Theoretically, it is essential to possess sufficient available ions and electrons in the composite cathode to maintain a perfect percolation and mediate the high tortuosity. A proper and optimum ratio of cathodes and SSEs in the composite cathode could ensure that the ionic transport pathways or electronic pathways are not hindered by each other.^[^ ^566^, ^567^, ^568^ ^]^ Previous studies^[^ ^569^ ^]^ have suggested that a high cathode weight ratio of 70:30 between cathodes and SSEs could achieve optimized performance. In this study, the ASSLIBs with an optimized ratio of 51:49 wt.% (40/60 vol%) in the LMO/LIC composite cathode exhibited a close to unity ratio of ionic conductivity/electronic conductivity (σ_ion_/σ_electron_) and exhibited the best electrochemical performance as shown in Figure 11I. Consequently, the ratio‐optimized ASSLIBs delivered an initial discharge capacity of ≈95 mAh g^−1^ with a stable capacity for 50 cycles with capacity retention of 92% (Figure 11J). In addition, when designing the high‐performance ASSLIBs, careful consideration should be given to the particle sizes and the intrinsic properties of both cathodes and SSEs, since partial conductivities and interfacial resistance were not linearly scaled with the volume fractions.^[^ ^525^, ^570^ ^]^

Lee et al.^[^ ^571^ ^]^ demonstrated that Fe‐doped LNMO (LiNi_0.476_Mn_1.475_Fe_0.049_O_4_/Fe‐LNMO) could enhance LNMO spinel structural stability and both ionic and electronic conductivity. When a trivalent cation (i.e., Fe^3+^) is doped into the octahedral sites of LNMO, its high‐spin t_2_^3^e^2^ electronic configuration causes partial occupancy of tetrahedral sites in LNMO, improving the spinel structural stability.^[^ ^572^, ^573^, ^574^ ^]^ Both the sulfide and halide SSEs were studied when paired with Fe‐LNMO. Due to the chemical and electrochemical incompatibility, LNMO and Fe‐LNMO with LPSCl sulfide SSEs delivered a low initial discharge capacity of 5.5 and 19.2 mAh g^−1^ at 0.05C, respectively. Even when employing an LPO coating with an expanded oxidative stability, as shown in Figure 11K, the ASSLIBs could only deliver the initial discharge capacity of 23.4 mAh g^−1^ and an initial coulombic efficiency of 57.6%. In addition, due to the chemical incompatibility leading to severe interfacial resistance and unsatisfactory lithium‐ion transport kinetics, the battery failed after the first cycle. Meanwhile, when pairing halide SSE (Li_3_InCl_6_) with LPO‐coated Fe‐LNMO, the ASSLIBs delivered an initial discharge capacity of 94 mAh g^−1^ and the corresponding Coulombic efficiency of 85.4%. Notably, the LPO@Fe‐LNMO demonstrated a capacity retention of 68.1% after 100 cycles, outperforming the undoped one with a capacity retention of 56.0%, as shown in Figure 11L. The improved Ni^2+^ redox reaction plateau at 4.5 V further confirmed the effectiveness of the cathode modification: Fe‐doping enhanced the structural and interphase stability, and LPO coating improved the charge‐transfer kinetics, passivated the decomposition of SSE, and stabilized the interface during cycling.^[^ ^503^ ^]^

In conclusion, halide SSEs possess notable advantages, including high ionic conductivity and oxidation stability, making them promising candidates for pairing with Mn‐based spinel cathodes in ASSLIBs. Surface coating is an effective strategy to mitigate electrochemical incompatibility, especially with high‐voltage LNMO cathodes. Further advancements in enhancing the room‐temperature ionic conductivity, broadening the electrochemical stability window, and refining synthesis methods could further position the halide SSEs to be more competitive with other SSE counterparts.

### 3.6 LiPON Thin‐Films Solid‐State Electrolytes

#### 3.6.1 Brief Introduction of LiPON Thin Films Solid‐State Electrolytes

Lithium phosphorus oxynitride (Li_3.2_PO_3.8_N_0.2_/LiPON) was discovered as early as the 1990s by Bates et al.^[^ ^575^ ^]^ and then rapidly developed as a solid‐state electrolyte in micro‐thin film battery applications.^[^ ^576^, ^577^ ^]^ LiPON exhibits various favorable electrochemical advantages, including excellent thermal and chemical stability, great compatibility with Li anode, low electronic conductivity (10^−13^–10^−14^ S cm^−1^), prolonged cycling life, and wide electrochemical windows (≈5.5 V).^[^ ^575^, ^578^, ^579^, ^580^, ^581^, ^582^, ^583^ ^]^ Notably, there are still several challenges that need to be addressed before realizing wider applications. These challenges include its relatively low ionic conductivity (10^−8^–10^−6^ S cm^−1^), large interfacial resistance, and low energy/power density.^[^ ^584^, ^585^, ^586^ ^]^

#### 3.6.2 Strategies to Address the Challenges

##### Enhancing the Ionic Conductivity

The ionic conductivity of the SSEs plays an extremely important role in determining the mobility of the Li‐ions and is closely related to the overall electrochemical performance. However, due to the nature of the amorphous structure, it is quite challenging to clearly explain the mechanism of ion transportation within LiPON. Tremendous efforts have been made to better understand the fundamental mechanism within the LiPON structure, and hence to tailor efficient strategies to improve ionic conductivity and electrochemical performance. In the early work from Bates et al.,^[^ ^587^ ^]^ the R.F. magnetron sputtering synthesized LiPON thin film‐based thin‐film batteries demonstrated ionic conductivity of 2 × 10^−6^ S cm^−1^. Compared with the precursor Li_3_PO_4_ with ionic conductivity of 1.4 × 10^−6^ S cm^−1^, the formation of P═N and P─N from the nitrogen substitution of oxygen was found to contain a higher covalent character than P‐O bonds, indicating the effectiveness of the presence of P and N bonds in increasing ionic conductivity.

Muñoz's group,^[^ ^588^, ^589^ ^]^ investigated the influence of nitrogen incorporation on the structure and ionic conductivity of glasses xLi_2_O•(100 − x)P_2_O_5_ (x = 38–60 mol%). This work revealed the structural changes induced by nitridation. At room temperature, the ionic conductivity was measured at ≈3.7 × 10^−6^ and 5.5 × 10^−4^ S cm^−1^, when x = 38 mol% and 60 mol%, respectively. The high ionic conductivity was attributed to a higher lithium oxide content (60 mol%), which possessed the highest amount of Li‐ion. In addition, it was found that the increase in ionic conductivity was correlated to both the nitrogen and lithium content, such that with the same nitrogen content, the lower the lithium content, the more significant the improvements in ionic conductivity. The sample with 60 mol% Li_2_O only demonstrated 0.55 orders of magnitude increment (9.1 × 10^−4^ S cm^−1^), while the 38 mol% sample enhanced the ionic conductivity by ≈2 orders of magnitude to 1.0 × 10^−5^ S cm^−1^.

Ceder's group studied the relationship between composition and structural changes due to N substitution and the ionic conductivity of LiPON electrolytes through ab initio molecular dynamics (AIMD) simulations.^[^ ^590^, ^591^ ^]^ From the simulation studies, it was found that the ionic conductivity can be improved by increasing the Li sites’ energy. The calculated result in the formula of Li_2.94_PO_3.50_N_0.31_ achieved the highest ionic conductivity of 1.5 × 10^−4^ S cm^−1^. The maximum Li‐ion mobility could be achieved by maintaining high charge carrier concentrations and N in bridging sites while suppressing the amount of isolated O anions and the excess Li in Li_3_PO_4_. These studies provided important insights into the ion transportation mechanism and mobility from a theoretical perspective.

Elevating temperature is an efficient strategy to improve ionic conductivity. Through atomistic simulations, Liu's group studied the influence of temperature and the size of nanoparticles on Li‐ion transportation in LiPON.^[^ ^592^ ^]^ The simulations revealed that the ionic conductivity of LiPON had a strong and close relationship with temperature, particle size, and film thickness. The electrostatic correlation interactions between Li‐ions increased as the temperature increased, accelerating the ionic 3D flow. It was also found that the highest ionic conductivity was achieved when the thickness of the LiPON film was less than 10 nm. The optimized particle size of 3.06 nm exhibited the highest room temperature ionic conductivity of 10^−6^ S cm^−1^, which was also verified through experimental tests. Chevalier et al.^[^ ^577^ ^]^ systematically studied the effectiveness of temperature and ionic conductivity within a wide temperature range (−200 to 100 °C). At a low temperature of −120 °C, ionic conductivity could only reach 10^−9^ S cm^−1^; meanwhile, the room temperature ionic conductivity was achieved as 2 × 10^−6^ S cm^−1^. The highest ionic conductivity of 5 × 10^−5^ S cm^−1^ was achieved at 100 °C. Indeed, the higher temperature provides more thermal activation energy, increasing the thermodynamically ionic mobility and ionic conductivity accordingly.

Attracted by the excellent intercalation kinetics and the stable electrochemical performance of the LMO thin films, Lee et al.^[^ ^593^ ^]^ developed all‐solid‐state thin film batteries (ASSTFBs) with LMO as cathode and LiPON as electrolyte. The LiPON with ionic conductivity of 2 × 10^−6^ S cm^−1^ and a thickness of 1 µm was deposited onto a 0.3 µm‐thick LMO thin film via RF magnetron sputtering. The ASSTFBs demonstrated ≈200 mV polarization in the first cycle and delivered an initial discharge capacity of ≈48 µAh cm^−2^‐µm under a current density of 0.1 mA cm^−2^. In addition, the TFBs also exhibited excellent long‐cycle stability with 96% capacity retention after 100 cycles, which was competitive to the liquid counterparts of 98%.^[^ ^594^ ^]^

##### Reducing Interfacial Resistances

To better understand the mechanism of large interfacial resistance between the electrode and the electrolyte in the TFBs, Iriyama et al.^[^ ^595^ ^]^ investigated the Li‐ion transfer reaction at the interface within the all‐solid‐state TFBs with 60 nm thick highly crystalline LMO as a thin film cathode and LiPON as a thin film electrolyte. A new phase near the LMO electrode surface was identified after the deposition of LiPON film, which was considered the origin of the formation of large interfacial resistance. The interfacial resistance of the TFBs after 500 cycles under 1 µA was enlarged 100 times and was measured at 15 000 Ω cm^2^, which was more than three times higher than the liquid counterpart (4200 Ω cm^2^). In addition, the TFBs demonstrated an excellent capacity retention of 80% initial discharge capacity compared to the liquid counterpart of ≈25%, which further evidenced the stability of the interface and inhibition of Mn dissolution.

**Figure 12:**
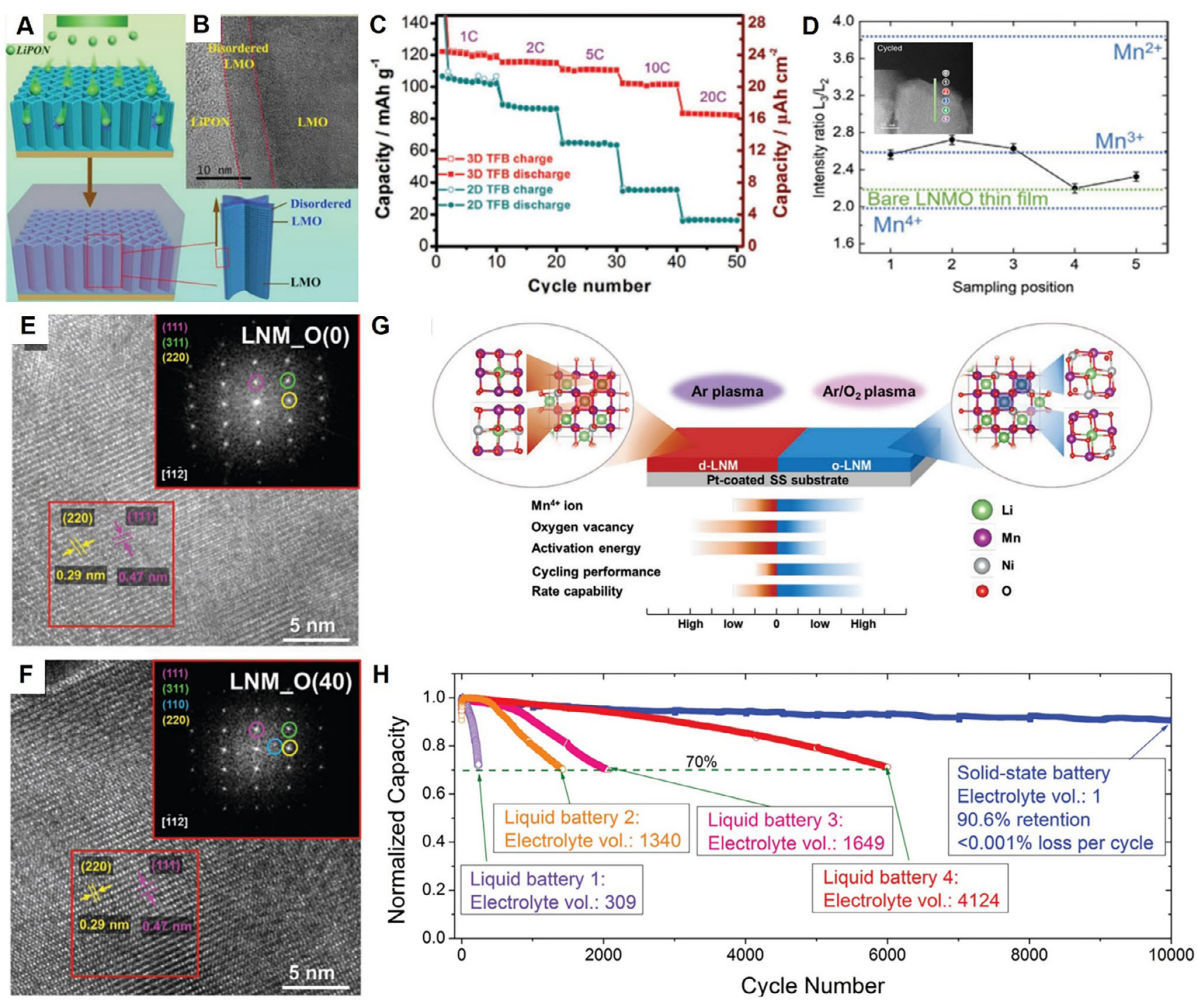
A) Schematic illustration of LiPON sputtering onto 3D nanowall arrays of LMO with reduced structural distortion. B) The corresponding HRTEM image of the interface between LiPON/LMO. C) Comparison of rate capability between 3D TFB and 2D TFB. Reproduced with permission.^[^ ^597^ ^]^ Copyright 2018, Wiley. D) The intensity ratio of Mn L_3_/L_2_ at the cycled LNMO/LiPON interface and the corresponding locations are shown as an inset. Reproduced with permission.^[^ ^602^ ^]^ Copyright 2022, Wiley. E,F) HRTEM images of post‐annealed LNMO under Ar (LNMO_0) and Ar/O_2_ of 60:40 (LNMO_40). G) Schematic illustration of the comparison between D‐LNMO and O‐LNMO thin film cathodes. Reproduced with permission.^[^ ^611^ ^]^ Copyright 2022, Wiley. H) Long cycling capacity retention comparison between LNMO/LiPON TFBs with liquid counterparts. Reproduced with permission.^[^ ^613^ ^]^ Copyright 2014, Wiley.

Chang et al.^[^ ^596^ ^]^ introduced the checkerboard deposition, which transformed the RF magnetron sputtering amorphous LMO thin film into a crystalline phase to facilitate an enlarged contact area between the LMO cathode and the LiPON electrolyte. The fabricated LMO thin film enabled more Li‐ion transport pathways that allowed more Li‐ion exchange under high current density and reduced the polarization effect simultaneously. Consequently, the all‐solid‐state TFB delivered a discharge capacity of 107.8 µAh at 11 µA cm^−2^ (0.5C) and demonstrated superior cycling stabilities with capacity retention of 95% after 150 cycles. Xia et al.^[^ ^597^ ^]^ developed 3D all‐solid‐state thin film batteries to mitigate the challenges of restrained reaction interface between electrode/electrolyte and limited ionic transportation, which resulted in low energy and power within the conventional planar 2D thin film batteries, as shown in **Figure** **12**A. The constructed vertically aligned nanowall arrays 3D structure LMO thin film cathode significantly enlarged the interface area between the cathode and electrolyte, reduced Li‐ion diffusion length, and accelerated ion transportations.^[^ ^598^, ^599^ ^]^ It is worth noting that RF magnetron sputtering deposited LiPON on the surface of the LMO film, leading to a change in the LMO crystalline structure. Through the high‐angle annular dark field scanning transmission electron microscopy (HAADF‐STEM) technique, a disordered amorphous LMO layer at the 3D nanowall arrays LMO and LiPON thin films interface was significantly reduced from 15 to 5 nm when compared with 2D TFBs (Figure 12B). The 3D‐TFBs delivered an initial capacity of 120 mAh g^−1^ under 1C, which was greatly improved from the conventional 2D TFBs of 100 mAh g^−1^. The 3D TFBs exhibited superior rate capability that delivered the discharge capacity of 83 mAh g^−1^ under 20C when compared to 16 mAh g^−1^ of 2D TFBs, as shown in Figure 12C. Moreover, the 3D ASSTFBs demonstrated excellent long‐cycle stability with a capacity retention of 90% after 500 cycles, whereas the 2D TFB could only maintain 73%. In addition, the 3D architecture facilitated enhanced structural and mechanical stability, which sufficiently mitigated the challenges of volume change of the cathode during cycling. The formation of this layer originated from the LiPON deposition process; the high kinetic energy from the sputtered LiPON energized the adatoms at the surface of the LMO film, hence changing the crystal structure of LMO.^[^ ^600^, ^601^ ^]^

Meng et al.^[^ ^602^ ^]^ systematically studied the LNMO cathode and LiPON electrolyte interface in the ASSTFBs. The ASSTFBs demonstrated an excess initial charge capacity of ≈32.5 µAh cm^−2^ and a discharge capacity of ≈12.5 µAh cm^−2^. The overlithiation resulted in an extended charging plateau over 4.7 V, which led to the observation of Mn reduction from +4 to +3, originating from the chemical reactions caused by LiPON deposition. And subsequently, the Mn^3+^ was oxidized to Mn^4+^ during the lithium extraction in the following discharge process. In addition, the TFBs exhibited superior stable cycling performance with an average coulombic efficiency of ≈99.6% between the second and 600th cycles. The stability of the interface was further confirmed by the void‐ and crack‐free interface, without the observation of Mn dissolution phenomena. As shown in Figure 12D, the intensity ratios of Mn L_3_/L_2_ from EESL spectra for post‐500 cycles LNMO/LiPON interface were maintained at 2.6 or lower, suggesting the valence of Mn was maintained at +3, which evidenced the electrochemical stability between LNMO and LiPON.^[^ ^603^ ^]^ The corresponding sampling positions were shown within the Cyro‐STEM images as an inset.

Engineering the crystal facet of the active materials, especially the exposed crystal facets, could tune the Li‐ion intercalation/de‐intercalation process during the operation of the batteries. For instance, the Li‐ion migration pathways facilitated by the favorable exposed facets enabled the anisotropic Li‐ion diffusion to achieve 100% theoretical capacity.^[^ ^604^, ^605^, ^606^ ^]^ Among the crystallographic orientations of conventional LNMO, the (100), (111), and (110) facets exhibited dominance within the structure. Previous studies^[^ ^607^, ^608^, ^609^ ^]^ provided various invaluable suggestions for the functions of each surface plane. However, due to the complexity of the materials, such as particle size, crystallinity, cation ordering, etc., the discrepancy persisted. For instance, one study suggested the (111) surface was more Li‐ion transportation favorable and more stable than the (100).^[^ ^608^ ^]^ While another one indicated the (100) plane possessed superior Li‐ion transportation kinetics than the (111) plane.^[^ ^607^ ^]^ To gain a better understanding of how the exposed facets would affect the electrochemical performance, Park et al.^[^ ^610^ ^]^ systematically studied the relationship between the exposed crystal facets (100), (111), and (110) of LNMO and the nano‐interface. It was found that the (100)‐oriented LNMO ASSTFBs under 0.5C delivered an initial discharge capacity of 114.6 mAh g^−1^, higher than that of (111)‐oriented 109.9 mAh g^−1^, which was attributed to the faster Li‐ion diffusivity within (100)‐dominant facets than (111)‐dominant facets. In addition, the ASSTFBs with (100)‐oriented LNMO exhibited a capacity retention of 103.8 mAh g^−1^ (90.6%) after 100 cycles, while (111)‐oriented LNMO demonstrated a capacity retention of 109.4 mAh g^−1^ (99.6%). The (111) facets could also sufficiently suppress the Mn dissolution and interfacial diffusion, hence demonstrating the capability of stable long cycling.

It is well‐recognized that the phase transformation between the disordered phase and the ordered phase can be induced by annealing.^[^ ^191^ ^]^ Accordingly, the ordering of cations (Ni and Mn) changes associated with the phase transformation during the LNMO deposition process. By controlling the oxygen flow rate in oxygen vacancies (Vo), Kim et al.^[^ ^611^ ^]^ investigated the correlation of the electrochemical performance between the orders of cations and the oxygen vacancies during the LNMO thin film fabrication. It was observed that during the RF‐sputtering deposition, increasing the flow rate of O_2_ resulted in reduced oxygen vacancies and elevated cation ordering, leading to the structure change from a disordered to an ordered one. From the HR‐TEM images shown in Figures 12E,F, the (111) and (220) planes were observed regardless of the presence of O_2_ flow. The FFT image of optimized LNMO, which was annealed under an Ar: O_2_ flow ratio of 60:40 sccm (LNMO_40), showed an extra (110) plane in addition to the common (111), (311), and (220) planes of annealed LNMO without O_2_. The ASSTFBs with 100 nm thickness of LNMO_40 delivered an initial discharge capability of 276.9 mAh cm^−3^ under 1 C and superior long‐cycling performance of 93.9% capacity retention (260.0 mAh cm^−3^) after 500 cycles. In addition, the DFT calculations revealed that the formation of V_O_ was more facile in the disordered than the ordered, which originated from a lower formation energy of V_O_ in the disordered structure. It was elucidated that the Li atom vacancy formation energies at the position near V_O_ were lower, which originated from the V_O_ hindering the Li‐ion diffusion rate by being more likely to trap Li‐ions near it.^[^ ^612^ ^]^ Therefore, the Li diffusion within a disordered structure of more easily V_O_‐forming was more difficult than in an ordered one, which was further evidenced by the optimum electrochemical performance achieved at the ordered structure with fewer oxygen vacancies (Figure 12G).

The challenge of the large interfacial resistance persists when coupling LiPON with high‐voltage cathode LNMO. Iriyama et al.^[^ ^373^ ^]^ examined the ASSTFBs with Mn‐based spinel cathode LiCr_0.05_Ni_0.45_Mn_1.5_O_4‐δ_ (LCNMO) and LiPON electrolyte. Due to the large electric field at the interface, the ASSTFBs could not demonstrate any charge or discharge electrochemical performance according to CV tests, which originated from the large interfacial resistance of more than 107 Ω·cm^2^. Therefore, dielectric BaTiO_3_ nanoparticles were introduced at the LiPON/LCNMO interface. The optimized ASSTFBs with 0.008 wt.% (100 nm) of BaTiO_3_ delivered a discharge capacity of 120 mAh g^−1^ at 0.25C, outperforming the unmodified ASSTFBs of 45 mAh g^−1^. The interfacial resistance was significantly reduced by 4 orders of magnitude to 2 × 10^3^ Ω·cm^2^.

Dudney et al.^[^ ^613^ ^]^ for the first time, demonstrated the successful superior stable, long cycling all‐solid‐state TFBs with LNMO as cathode and LiPON as electrolyte. Generally, the challenge of the low ionic conductivity of the SSEs in the ASSLIBs was addressed by the reduced thickness of the electrolyte layer. Therefore, in this work, by employing a thickness of 1 µm LiPON film, the ASSTFBs delivered a reversible capacity of 122 mAh g^−1^ under 0.1C. The ASSTFBs exhibited capacity retention of 90% over 10 000 cycles under 5C with an average of more than 99.98% coulombic efficiency. In contrast, the liquid electrolyte counterparts with less than 99.5% coulombic efficiency with electrochemical decomposition during the cycling achieved capacity retention of 70% within 6000 cycles (Figure 12H). Through SEM cross‐section images of the post‐1000 cycles sample, a conformal and smooth interface between LNMO and LiPON was observed without Mn dissolution. The interfacial resistance, which was estimated as half of the overall resistance of the solid‐state battery, was evaluated at 102 Ω cm^2^ and 115 Ω cm^2^ after the first cycle and 4000 cycles, respectively, further evidencing the stability of the interface.

In conclusion, the advantages of excellent chemical and electrochemical stability, great compatibility with cathode and anode, and the wide electrochemical stability window are undoubtedly pushing LiPON to be an excellent candidate in the applications of ASSLIBs. The existing challenges, such as the low ionic conductivity and significant interfacial resistance, are mitigated by strategies such as elevating the operating temperature, tuning the microstructures, and employing novel 3D structures. Nevertheless, there are still some remaining challenges that need to be tackled, such as the extra annealing procedure after deposition to maintain the crystallinity of LMO. However, when applying commercialized microelectronic devices, high temperatures are not favorable for complementary metal oxide semiconductors (CMOS) and low‐cost plastic substrates.^[^ ^614^ ^]^ An ideal manufacturing process should not only be capable of maintaining the homogeneity, conformity, and crystallinity of the cathode material but also have a low processing temperature. Secondly, the active materials loading still needs to be increased to be competitive with liquid counterparts. For instance, Dudney et al.^[^ ^613^ ^]^ suggested that the demonstrated ASSTFBs with a scant cathode loading of 0.5 mg cm^−2^ were approximately 10 times smaller than the commercial liquid LIBs. Lastly, the cost of manufacturing the ASSTFBs is still generally high. Increasing the ionic conductivity of the employed electrolytes, enlarging the energy density, and topping the cathode active material loading while suppressing the TM dissolution are still urgently required to elevate the overall performance of the ASSLIBs.

## 4 Conclusions and Future Perspectives

In this review, we began by examining the unique structural characteristics of Mn‐based spinel cathodes and their advantages in the applications of ASSLIBs. The electrochemical behavior and the degradation mechanism of the Mn‐based spinel cathodes are also comprehensively discussed. Despite the stable spinel structures during Li‐ion insertion and extraction processes, several challenges persist in ASSLIBs, including Mn dissolution/diffusion at the cathode/SSE interface and the electrochemical/chemical incompatibility, resulting in the formation of large interfacial impedance. Overall, LMO operates at ≈4.1 V vs Li^+^/Li, exhibits a cubic spinel structure with 3D Li‐ion diffusion channels. Its main challenge arises from Jahn–Teller distortion of Mn^3+^ and Mn dissolution during cycling, which can induce surface reconstruction and oxygen loss. By replacing a portion of Mn^3+^ with Ni^2+^, LNMO exhibits a high voltage of ≈4.7 V. The reduction in Mn^3+^ content suppresses Jahn–Teller distortion, but the high operation voltage introduces severe interfacial instability and oxidative decomposition of most SSEs. The interfacial evolution of LMO and LNMO is summarized and listed in **Table** **2**.

**Table 2:** Comparison of interfacial evolution between LMO and LNMO.

Feature	LMO	LNMO
Mn Valence Behavior	High Mn^3+^ content triggers Jahn–Teller distortion and disproportionation to Mn^2+^ (soluble) and Mn^4+^.	Ni doping reduces Mn^3+^ content, but cycling still promotes Mn^2+^ formation at the surface.
Interfacial Chemistry	Mn^2+^ diffuses into SSEs (e.g., LGPS), triggers interface decomposition and formation of resistive interphases.	High voltage (>4.3 V) leads to oxidative decomposition of SSEs (e.g., LPSCl), resulting in the formation of sulfur species and dense CEI layers.
CEI/Interphase Formation	Driven by Mn dissolution, oxygen vacancy formation, and subsequent migration into SSEs.	Accelerated by high‐voltage instability, the formation of Mn_3_O_4_‐like or rock‐salt phases.
Phase Heterogeneity	Multiple phase formation (e.g., spinel to layered or amorphous phases) is often localized at grain boundaries.	Surface reconstruction and rock‐salt‐like phases originate from transition metal migration.

Subsequently, we meticulously discussed the integration of Mn‐based spinel cathodes coupled with various SSEs in the ASSLIBs. We also included the potential decomposition products and the possible reactions at the cathode/SSE interface for the representative SSEs of oxide‐, sulfide‐, and halide‐SSEs when pairing with LMO and LNMO during the electrochemical cycling, as in **Table** **3**.

**Table 3:** Comparison of SSE decomposition products and possible side reactions.

SSE	Decomposition reaction byproducts	Possible reactions with LMO/LNMO
Li_7_La_3_Zr_2_O_12_	La_2_O_3_, La_2_Zr_2_O_7_, and Li_2_O^[^ ^514^ ^]^	LNMO at 4.7 V may cause oxygen loss; it forms resistive LaMnO_3_‐type phases.
Li_1.3_Al_0.3_Ti_1.7_(PO_4_)_3_	Ti_2_O_7_, TiP_2_O_7_, Li_3_PO_4_, and Al_2_O_3_^[^ ^514^ ^]^	LMO triggers Al^3+^/Ti^4+^ interdiffusion; Ti^3+^ can catalyze further decomposition.
Li_6_PS_5_Cl	Li_3_PS_4_, P_2_S_5_, Li_2_S, Li_3_P, S, and LiCl^[^ ^615^ ^]^	Mn^3+^/Mn^2+^ may oxidize S^2–^ → S^0^; forms Mn–S, MnCl_2_, or MnPS_3_^−^ like at interphases.
Li_10_GeP_2_S_12_	Li_3_PS_4,_ Ge, S, P_2_S_5_, and Li_2_S^[^ ^514^ ^]^	Ni^4+^/Mn^4+^ redox may oxidize S^2–^; Ge^0^ diffusion can form insulating layers.
Li_3_InCl_6_	LiCl, InOCl, In_2_O_3_, and Cl_2_^[^ ^616^ ^]^	Oxygen loss from LMO/LNMO may result in the formation of In_2_O_3_.
Li_3_YCl_6_	LiCl, Y_2_O_3_, and Cl_2_^[^ ^616^ ^]^	High operating voltage of cathodes LMO/LNMO, may result in the formation of Y_2_O_3_ due to oxygen loss.

**Figure 13:**
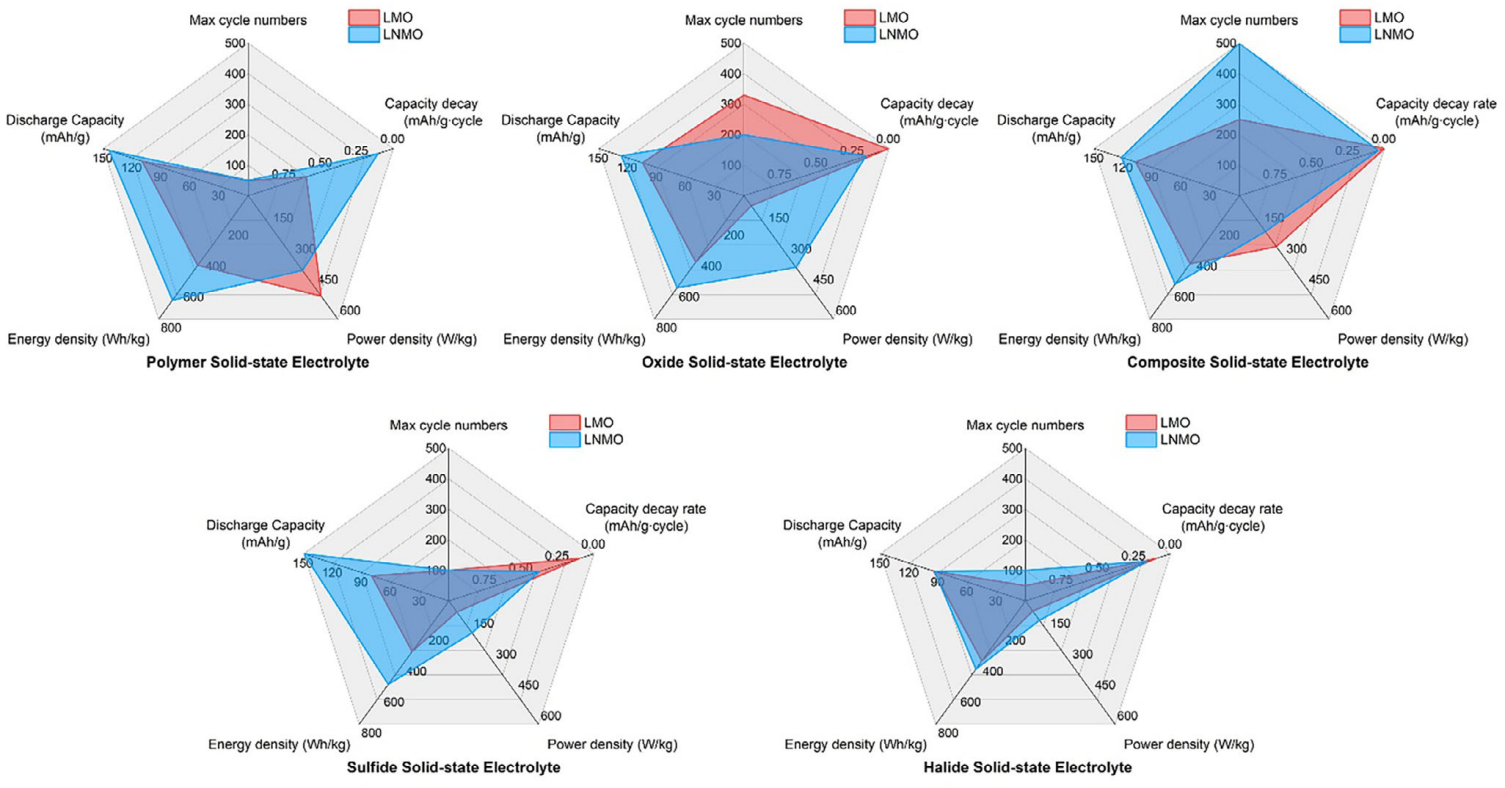
An electrochemical performance comparison of reported LMO/LNMO cathodes with different solid‐state electrolytes in ASSLIBs.

In addition, the primary challenges associated with polymer‐based ASSLIBs utilizing Mn‐based spinel cathodes are electrochemical incompatibility and limited room‐temperature ionic conductivity. To overcome these challenges, various strategies have been developed, encompassing increasing operation temperature, incorporating co‐polymer, optimizing microstructure, and adding Li‐salt and inorganic fillers. When pairing with oxide SSEs, side reactions during the co‐sintering or heat treatment, and the electrochemical incompatibility are the key challenges. To mitigate these challenges, approaches such as optimizing fabrication methods, employing 3D microstructures, and incorporating polymer electrolyte binders have been developed. The introduction of inert/active filler is the key strategy to improve the Li‐ion conductivities, thus facilitating fast Li‐ion transport at the cathode/SSE interface. Sulfide SSEs demonstrated chemical incompatibility and electrochemical instability when paired with Mn‐based cathodes. Coating and microstructure modifications are the mainstream strategies to mitigate these challenges. In addition, incorporating sulfide SSEs as active fillers into polymer‐based composite SSEs has proven to be an effective strategy to enhance interfacial ion‐conductivities. Halide SSEs exhibit electrochemical incompatibility with high‐voltage LNMO; thus, applying protective coatings is the most effective and practical strategy to overcome this challenge. Finally, we discussed the challenges associated with LiPON thin‐film SSEs in ASSTFBs, particularly their low ionic conductivity and large interfacial resistance. When paired with Mn‐based spinel cathodes, commonly developed strategies to address these challenges include elevating the operating temperature, optimizing microstructures, and constructing novel 3D architectures. Additionally, the current progress of spinel cathodes LMO‐ and LNMO‐based ASSLIBs, and their electrochemical performance are listed in **Tables** **4** and **5**, respectively. Subsequently, the comparison of the reported electrochemical performance of different solid‐state electrolytes with LMO and LNMO, in terms of the number of maximum cycles, capacity decay rate, power density, energy density, and initial discharge capacity, is shown in **Figure** **13**.

**Table 4:** LMO‐based ASSLIBs and their electrochemical performance.

Solid‐state electrolyte	Cathode loading [wt.%]	Anode	Voltage range[V vs Li/Li^+^]	Performance in the initial cycle (current density; initial discharge capacity; initial Coulombic efficiency)	Long cycling performance (cycles; capacity retention)	Rate capability	Temperature [°C]	Year	Refs.
Polymer solid‐state electrolytes									
Polystyrene‐based	30%	Li	3.0‐4.3	0.05 C; 110 mAh g^−1^; 40%	50; 72.3%	80 mAh g^−1^ (0.2 C)	50	2023	[264]
Oxide solid‐state electrolytes									
Li_3_PO_4_	100 nm	Si	3.7‐4.3	0.05 C; 190 µAh cm^−2^	50; 95%	4.5 µAh cm^−2^ (1 C)	–	2022	[354]
Li_1.5_Al_0.5_Ti_1.5_(PO_4_)_3_	60% ratio	Li	3.6‐4.3	0.1 C; 65 mAh g^−1^; 94%	–	–	25	2008	[377]
Li_0.35_La_0.55_TiO_3_	80% ratio	Li	3.0‐4.3	0.0016 C; 82 mAh g^−1^; 75%	–	–	25	2009	[375]
Li_0.35_La_0.55_TiO_3_	70% ratio	Li	3.0‐4.7	0.1 C; 71 mAh g^−1^; 70%	–	–	30	2011	[376]
LLZO/LiTFSI	80%	Li	3.0‐4.5	0.085 C; 104.26 mAh g^−1^; 98.85%	360; ‐	23.77 mAh g^−1^	‐	2023	[385]
Ga‐LLZO/SIL	80%	Li	3.0‐4.5	0.1 mA cm^−2^; 105 mAh g^−1^; 98%	572; 94%	32.87 mAh g^−1^	27	2024	[386]
Composite solid‐state electrolytes									
PVDF/LLZTO	80%	Li	3.0‐4.3	50 mA/g; 67 mAh g^−1^; 75%	100; 88%	–	25	2020	[428]
HBPAE‐g‐TiO_2_ /PEO	85%	LiTi_2_(PO_4_)_3_	2.5‐4.3	0.1 C; 78 mAh g^−1^; 82%	–	35 mAh g^−1^ (5 C)	RT	2016	[437]
PEO‐LiTFSI /LiAlSiO_4_	80%	Li	3.0‐4.3	0.5 C; 100 mAh g^−1^; 99%	100; 50%	–	30	2021	[446]
PVDF/HNT	70%	Li	3.4‐4.5	1 C; 71.9 mAh g^−1^; 75%	250; 100%	75 mAh g^−1^ (3.5 C)	30	2018	[459]
PVDF/LATP	80%	Li	3.0‐4.3	0.2 C; 117 mAh g^−1^; 97.70%	200; 91.4%	92 mAh g^−1^ (2 C)	RT	2018	[462]
P(VDF‐HFP) /LiZr_1.5_Sn_0.5_(PO_4_)_3_	–	Li	3.5‐4.3	0.1 mA cm^−2^; 106 mAh g^−1^; 94.60%	100; 93%	40 mAh g^−1^ (0.3 C)	–	2023	[466]
P(VDF‐HFP)‐LiTFSI/Y‐LZP	–	Li	3.5‐4.3	0.1 C; 104 mAh g^−1^; 94.50%	–	50 mAh g^−1^ (2 C)	–	2024	[468]
Sulfide solid‐state electrolytes									
Li_2_S‐P_2_S_5_	38.5%	In	3.0‐4.6	0.064 mA cm^−2^; 55 mAh g^−1^; 84.60%	100; 72.70%	–	25	2010	[94]
Li_6_PS_5_Cl	38%	Li‐In	3.3‐4.5	0.1 C; 73 mAh g^−1^; 81%	22; 54.80%	–	RT	2017	[506]
Li_2_S‐P_2_S_5_	38.5%	In	3.0‐4.6	0.064 mA cm^−2^; 72 mAh g^−1^; 84.70%	50; 96%	32 mAh g^−1^ (4 C)	25	2012	[508]
Halide solid‐state electrolytes									
Li_3‐x_Lu_1‐x_Zr_x_Cl_6_	70%	Li‐In	3.0‐4.3	0.1 C; 119.4 mAh g^−1^; 91.8%	80; 94.2%	42 mAh g^−1^ (2 C)	RT	2023	[565]
Li_3_InCl_6_	51%	Li‐In	3.4‐4.3	0.1 C; 98 mAh g^−1^; 93%	50; 91.8%	–	–	2023	[497]
LiPON thin film solid‐state electrolytes									
LiPON	0.3 um	Li	3.7‐4.3	100 µA cm^−2^; 48 µAh cm^−2^; 80%	100; 96%	45 µAh cm^−2^·µm	RT	1999	[593]
LiPON	60 nm	Li	3.5‐4.3	1 µA	500; 80%	–	60	2006	[595]
LiPON	1 um	Li	3.0‐4.3	0.5 C; 107.8 µAh	150; 95%	84.2 µAh (10 C)	25	2018	[596]
LiPON	450 nm	Li	3.3‐4.4	1 C; 121 mAh g^−1^	500; 90%	83 mAh g^−1^ (20 C)	RT	2018	[597]

**Table 5:** LNMO‐based ASSLIBs and their electrochemical performance.

Solid‐state electrolyte	Cathode loading [wt.%]	Anode	Voltage range [V vs Li/Li^+^]	Performance in the initial cycle (current density; initial discharge capacity; initial Coulombic efficiency)	Long cycling performance (cycles; capacity retention)	Rate capability	Temperature [°C]	Year	Refs.
Polymer solid‐state electrolytes									
PEO‐based	–	Li	3.3–5.0	0.1 C; 80 mAh g^−1^; 100%	150; 96%	–	60	2024	[249]
PMHS/HFBMA /PGAME	–	Li	3.6–4.9	0.5 C; 144 mAh g^−1^; 85%	30; 87.5%	–	60	2024	[251]
Polysiloxane‐based	80%	Li	3.5–4.9	0.1 C; 131.5 mAh g^−1^; 90%	50; 95.8%	–	30	2018	[226]
PMMA/PEG	75%	TiO2 nts	2.7–5.0	0.1 C; 169 mAh g^−1^; 65.59%	–	61 mAh g^−1^ (0.5 C)	–	2017	[280]
PMMA/PEG	75%	TiO2 nts	2.7–5.0	0.1C; 122 mAh g^−1^; 72%	–	47 mAh g^−1^ (0.5 C)	–	2019	[281]
Oxide solid‐state electrolytes									
Li_3_PO_4_	–	Li	3.8–4.8	1 C; 145 mAh g^−1^; ∼100%	100; ∼100%	73 mAh g^−1^ (3600 C)	70	2018	[370]
Li_3_PO_4_	40 nm	Li	2.5–4.8	1.4 C; 2 nA; 58%	50; 200%	–	–	2021	[371]
Li_3_PO_4_	60 nm	Li	3.5–4.8	1 C; 72 mAh g^−1^; 80%	90; 100%	8.1 mAh g^−1^ (1000 C)	–	2024	[372]
P(VDF‐HFP)‐Li_10_ /LATP	80%	Li	3.5–5.0	0.5 C; 126.8 mAh g^−1^; 99%	200; 75%	59.8 mAh g^−1^ (2 C)	RT	2022	[381]
PAALi/LAGP /PAALi	90%	RuO_2_	0.8–4.1	0.2 C; 87.5 mAh g^−1^; 20%	120; 74.2%	–	23.8	2019	[387]
Composite solid‐state electrolytes									
PES/LATP	80%	Li	3.5–5.0	0.1 C; 120 mAh g^−1^; 100%	50; 98%	25 mAh g^−1^ (1 C)	RT	2022	[424]
PES‐PVC‐PVDF /LATP	80%	Li	3.5–5.0	0.1 C; 122 mAh g^−1^; 94%	500; 82.5%	40 mAh g^−1^ (1 C)	RT	2022	[425]
PEO‐based	1 µm	Li	3.0–5.2	0.8 µA cm^−2^; 100 mAh g^−1^	–	–	60	2003	[470]
PEO‐MEEGE‐AGE/LPO	82%	Li	3.5–5.0	0.015 µA cm^−2^; 120 mAh g^−1^; 48%	–	–	60	2005	[471]
Sulfide solid‐state electrolytes									
Li_2_S‐P_2_S_5_	30%	In	3.5–4.8	0.064 mA cm^−2^; 62 mAh g^−1^; 100%	10; 100%	52 mAh g^−1^ (0.13 mA cm^−2^)	25	2016	[509]
Li_10_GeP_2_S_12_	40%	Li–In	3.5–5.0	0.05 C; 80 mAh g^−1^; 53%	20; 43.9%	–	25	2016	[521]
Li_10_GeP_2_S_12_	38.4%	Li–In	3.5–5.0	0.05 C; 90 mAh g^−1^; 75%	10; 44%	–	25	2016	[522]
Li_6_PS_5_Cl	70%	Li	3.5–5.0	0.1 C; 115 mAh g^−1^; 82.10%	20; 69.60%	–	–	2020	[498]
Li_6_PS_5_Cl	50%	Li	3.0–5.0	0.1 C; 77.9 mAh g^−1^	20; 87.50%	20 mAh g^−1^ (1 C) LTO anode	RT	2021	[524]
Li_6_PS_5_Cl	70%	Li–In	3.4–5.0	0.1 C; 105.5 mAh g^−1^; 86.50%	100; 62.10%	–	–	2022	[525]
Li_6_PS_5_Cl	70%	Li‐In	2.0–5.0	0.127 mA cm^−2^; 150 mAh g^−1^; 167%	–	–	25	2022	[526]
Li_10_GeP_2_S_12_	70%	Li‐In/LTO	2.5–5.5	0.3 C; 160 mAh g^−1^; 106.70%	100; 82%	–	RT	2020	[529]
Li_5.5_PS_4.5_Cl_1.5_	70%	Li‐Graphite	2.5–5.2	0.1 C; 85.5 mAh g^−1^; 90.53%	50; 85.2%	–	55	2022	[530]
Halide solid‐state electrolytes									
Li_3_YCl_6_	66%	Li‐In	3.5–4.85	7.5 mA/g; 91 mAh g^−1^; 91.20%	50; 46.15%	–	25	2022	[563]
Li_3_InCl_6_	65%	Li‐In	3.5–4.85	0.05 C; 94 mAh g^−1^; 85.40%	100; 68.10%	–	RT	2023	[571]
LiPON thin film solid‐state electrolytes									
LiPON	2.6 µm	Li	3.5–5.1	100 µA cm^−2^; 48 µAh cm^−2^; 80%	600	–	RT	2022	[602]
LiPON	–	Li	3.5–4.9	0.5 C; 110.4 mAh g^−1^; 55.00%	100; 97.6%	80 mAh g^−1^ (5 C)	–	2021	[610]
LiPON	100 nm	Li	3.5–4.9	0.5C; 276.9 mAh cm^−3^; 79.10%	500; 93.9% (1 C)	130 mAh cm^−3^ (5 C)	40	2022	[611]
LiPON	1 µm	Li	3.5–5.1	5 C; 125 mAh g^−1^; 75.70%	10 000; 90%	120 mAh g^−1^ (10 C)	RT	2014	[613]

Although various strategies have been developed for Mn‐based spinel cathodes in ASSLIBs, as we discussed in this review, some challenges remain at the material, interface, and electrode levels. We believe that the following directions hold promise for further improving the performance of Mn‐based spinel cathodes, thereby accelerating their practical applications in ASSLIBs:

### 4.1 Advancement of Mn‐Based Spinel Cathodes

#### 4.1.1 Enhancing Interfacial Contact

Unlike the excellent wettability of the liquid electrolytes in LIBs, one of the significant primary challenges in Mn‐based cathodes for ASSLIBs is the limited particle‐to‐particle contact between cathodes and SSEs. Future developments should prioritize improving interfacial contact. Reducing the particle size can significantly enlarge the contact area between the cathodes and SSEs, thereby reducing the Li‐ion diffusion length. Employing nanostructured cathodes, such as nanoparticles, nanowires, nanotubes, and nanoarrays, posed the drawback of low tap density in liquid LIBs but exhibited promise in ASSLIBs, particularly under pressurized operation.

#### 4.1.2 Optimizing Crystalline Morphology

Single‐crystal cathodes offer several advantages, including a larger specific contact surface area, lower tortuosity, reduced grain boundary, and more exposed facets. For instance, the function of the exposed crystal facets: (111), (110), and (100) in stabilizing the crystal structure and reducing the Mn dissolution in LIBs has been studied. The effectiveness of the truncated octahedral structure of Mn‐based spinel cathodes with designated exposed facets in the applications of ASSLIBs is still controversial and has not been thoroughly investigated.

Conversely, agglomerated polycrystalline cathodes possess reduced Li‐ion diffusion pathways between primary particles along with higher porosity. Optimizing the crystallinity of Mn‐based cathodes could combine the benefits of both morphologies, leading to enhanced performance.

#### 4.1.3 Facet Engineering

The exposed facets of the crystalline structure also play an important role in determining the electrochemical performance of the cathodes. Adopting truncated crystal structures can leverage the advantages of beneficial facets that have shown promise in liquid Mn‐based LIBs and should be considered within further designs of ASSLIBs cathodes.

#### 4.1.4 Suppressing Mn Diffusion/Dissolution

While existing strategies to mitigate the challenge of Mn diffusion/dissolution during prolonged cycling and elevated temperatures have shown some effectiveness, the challenge remains unresolved. The innovative modifications could include the strategies in synthesis. It was reported that utilizing the resorcinol‐formaldehyde route to prepare the LMO particles (bulk/nano) demonstrated a stable interface and suppressed dissolution.^[^ ^617^ ^]^ Furthermore, future research should prioritize the development of innovative multifunctional coatings, aliovalent doping, and surface reconstruction that are capable of completely preventing Mn from diffusing or dissolving both within the cathode structure and the cathode/SSE interface. For example, multifunction coatings should not only function as a protection layer to prevent the side reactions derived from cathode/SSE interfacial contact but also assist in suppressing the transition metal dissolution/diffusion. Aliovalent doping aims to maintain the average valence of Mn more than 3.5 to suppress the J‐T distortion. An electrochemically active cation doping (such as Ti^2+^, Ni^2+^) could further potentially enhance the overall cycling performance. Different from surface coating, which physically employs a barrier on the surface of the cathode, surface reconstruction,^[^ ^618^ ^]^ which involves modifications such as nano‐scale surface doping through chemical reaction evolution, is another strategy to tackle the challenge. The reconstructed surface is expected to maintain excellent electrochemical properties and is capable of reducing the TM dissolution. In addition, incorporating an electrolyte additive,^[^ ^619^ ^]^ which can form a protective layer on the surface of the cathodes through chemical or electrochemical reactions, is another approach.

#### 4.1.5 Widening Operating Voltage Ranges

While Mn‐based spinel cathodes offer the advantage of cost‐effectiveness, their energy density is lower than layered cathodes. Expanding the voltage range, particularly by lowering the lower cut‐off voltages, could unlock their potential for achieving higher capacities. However, this approach introduces challenges related to structural instability and irreversible spinel‐to‐rock‐salt phase transformations. To realize the possibility of expanding operating voltage ranges, innovative strategies need to be developed to stabilize the structure under the over‐discharge process, enabling Mn‐based cathodes to achieve high energy densities while maintaining long‐term cycling stability.

#### 4.1.6 Increasing CAM Loading

Realization of high‐loading cathodes in ASSLIBs remains a significant challenge due to issues such as limited interfacial Li‐ion/electron conductivity, as well as mechanical degradation associated with void formation caused by contact loss and volume change during the fabrication process and battery cycling. To address these challenges, a promising strategy is to develop binder‐free electrodes to facilitate fast and balanced Li‐ion/electron transportation. Additionally, constructing electrodes with 3D architectures is expected to mitigate mechanical stress, thereby improving the structural stability of ASSLIBs.

### 4.2 Design of Integrated SSEs

#### 4.2.1 Optimizing Low‐Temperature Ionic Conductivity

To ensure the SSEs are compatible with Mn‐based cathodes and suitable for EV applications. Obtaining satisfactory ionic conductivity (at subzero temperatures) is critically important. ASSLIBs require a minimum of 10−4 S cm^−1^ to support normal charge‐discharge processes. However, achieving a Li‐ion conductivity of at least 10−3 S cm^−1^ is crucial for maintaining long‐term cycling stability under relatively high current densities.

#### 4.2.2 Expanding the Electrochemical Stability Window

While most SSEs exhibit good electrochemical stability with LMO cathodes, challenges persist in all the cathode interfaces due to side reactions between high‐voltage LNMO cathodes and SSEs. Although surface coating is a widely used strategy to mitigate these side reactions, developing SSEs with inherently wide electrochemical stability windows offers a more direct and effective solution by addressing the issue at its root.

#### 4.2.3 Enhancing Mechanical Strength

Due to the inverse relationship between thickness and mechanical strength in SSEs, achieving a thick SSE layer with robust mechanical properties is critical yet challenging for realizing the targeted energy density of ASSLIBs. The pulverization caused by cracks severely deteriorates the Li‐ion transportation. Strategies for fabricating thin SSE layers should carefully address factors such as porosity, temperature, pressure, impurities, and stress distribution.

#### 4.2.4 Entropy‐Enthalpy Engineering

For polymer SSEs, the entropy‐enthalpy coordination disruption with the incorporation of poly(ionic liquid) demonstrates great potential in extending the electrochemical stability windows and enhancing ionic conductivities.^[^ ^620^ ^]^ High‐entropy oxide,^[^ ^621^, ^622^ ^]^ sulfide,^[^ ^544^, ^548^ ^]^ and halide^[^ ^623^, ^624^ ^]^ SSEs with generally increased configurational entropy through host lattice anion or cation disordering by halide or multicationic substitution demonstrate great potential in enhancing the physical, chemical, and electrochemical properties of the SSEs.

#### 4.2.5 Exploring Composite and Multilayer SSE Architectures

Developing composite SSEs or multilayer SSEs in the ASSLIBs with Mn‐based spinel cathodes, such as polymer/halide, sulfide/oxide, halide/oxide, and sulfide/halide, presents a promising approach to utilizing the unique advantages of each material. For example, polymer SSEs offer flexibility, oxide SSEs provide robust mechanical strength, sulfide SSEs exhibit superior room‐temperature ionic conductivity, and halide SSEs feature a wide electrochemical stability window. Composite polymer SSEs with inert/active fillers have demonstrated promising effectiveness in enhancing their electrochemical properties. Applying oxide electrolytes as active filler, it is extremely essential to select the proper plasticizer that possesses a higher dielectric constant than the electrolytes to achieve the effectiveness of improving the ionic conductivity. Additionally, incorporating ternary composites could further enhance performance through rational design, leveraging the synergistic properties of multiple SSE types

### 4.3 Integration of Advanced Characterization Techniques

Advanced characterization techniques are crucial for elucidating the intricate bulk and interfacial behavior of Mn‐based spinel cathodes in ASSLIBs, offering fundamental insights into the underlying mechanisms. However, the buried nature of cathode interfaces, which is combined with their dynamic, chemically reactive, and structurally complex characteristics, poses significant challenges to direct observation and analysis. Although a typical composite cathode consists of Mn‐based active materials, SSEs, and conductive additives, this system becomes far more intricate during practical electrochemical redox reactions. Both electrochemical and chemical reactions can trigger the degradation of cathodes and/or SSEs, and lead to the continuous formation of new interfacial phases and insulating side products.

To decipher these intricacies, advanced characterization techniques are extremely beneficial in probing critical phenomena, such as Li‐ion diffusion kinetics, Mn valence evaluation, CEI formation, and mechanical degradation. These insights are essential for improving the long‐term performance and stability of ASSLIBs.

#### 4.3.1 Capabilities and Resolution of Key Characterization Techniques

A comprehensive understanding of the structural and electrochemical evolution in ASSLIBs requires a multi‐scale approach using techniques that combine spatial and chemical sensitivity. i) Synchrotron‐based techniques. Advanced synchrotron tools such as X‐ray absorption spectroscopy (XAS), X‐ray diffraction (XRD, transmission X‐ray microscopy (TXM), and X‐ray computed tomography (X‐CT) enable operando and high‐resolution investigations across length scales from individual particles to full electrodes. These methods provide critical insights into redox state evolution, lattice structural changes, and morphological transformations during cycling. ii) Transmission electron microscopy (TEM) and scanning TEM (STEM). TEM/STEM, especially when integrated with electron energy loss spectroscopy (EELS), atomic‐resolution high‐angle annular dark field (HAADF) and bright field (BF) imaging, and 4D‐STEM, delivers atomic‐level information. These techniques are indispensable for visualizing lattice distortions, interfacial phase transformations, and structural evolution at the atomic scale. iii) Solid‐state nuclear magnetic resonance (NMR). Solid‐state NMR provides atomically resolved insights into local chemical environments, lithium‐ion mobility, and short‐range disorder in both cathode and SSE phases, offering valuable information on ionic transport mechanisms. iv) Time‐of‐flight secondary ion mass spectrometry (TOF‐SIMS) and X‐ray photoelectron spectroscopy (XPS). TOF‐SIMS and depth profiled XPS enable spatially resolved chemical mapping of interfaces, revealing interfacial reactions, elemental diffusion gradients, and the composition of CEI.

#### 4.3.2 Unresolved Scientific Questions in Mn‐Based Spinel Cathode Systems

Despite recent progress, several fundamental questions regarding the interfacial chemistry and degradation mechanisms of Mn‐based spinel cathodes within ASSLIBs remain unraveled. i) Mechanism of Mn transport. It remains unclear whether Mn migration in solid‐state systems follows a mechanism analogous to Mn dissolution observed in liquid electrolytes or whether it is governed by distinct interfacial redox reactions and solid‐state diffusion pathways associated with phase transformations. ii) Valence state and impact of migrating Mn. Although Mn diffusion into sulfide‐based SSEs has been consistently observed and reported, the valence state of the migrating Mn species remains uncertain. Is Mn^2+^, noted for its high mobility, the predominant species driving interfacial diffusion and degradation? Moreover, the mechanistic pathway by which Mn ions trigger or accelerate SSE decomposition is still poorly understood. iii) Influence of local gradients and chemical environment. The extent to which local electrochemical potential gradients and variations in chemical environment govern selective ion migration, CEI formation, and the development of interfacial impedance is still an open question. iv) Role of oxygen loss and structural instability. The contribution of oxygen loss and structural destabilization within Mn‐based cathodes to be initiation or acceleration of interfacial degradation processes remains inadequately explored.

These unresolved questions underscore the critical need for advanced characterization techniques that offer spatial resolution, chemical specificity, and temporal sensitivity, enabling clear differentiation between bulk processes and interfacial phenomena.

#### 4.3.3 Current Opportunities and Challenges in Applying Advanced Characterization Techniques to ASSLIBs

i) Material sensitivity and handling: Many SSEs, particularly sulfide‐ and halide‐based types, are extremely sensitive to air and moisture. This necessitates complex sample preparation, controlled transfer procedures, and measurements in inert, vacuum, or synchrotron beamline environments. ii) Mechanical stability and interface integrity: Maintaining uniform pressure and mechanical contact throughout electrochemical cycling and measurement is essential to preserve realistic solid‐state interfaces. Inconsistent pressure can significantly alter interfacial behavior and data reliability. iii) Cell design compatibility: Developing electrochemically functional mode cells that are compatible with X‐ray or electron beam setups requires substantial engineering. Challenges include optimizing material thickness, ensuring adequate beam transmission, and achieving robust sealing without compromising electrochemical performance. iv) Data correlation and reproducibility: Although in situ and operando techniques enable real‐time observation of morphological changes, stress evolution, and charge/discharge dynamics, correlating these observations with consistent electrochemical performance remains difficult. Variability in interfacial contact, pressure distribution, and cell configuration often introduces measurement inconsistencies and hinders reproducibility.

### 4.4 Opportunity for the Anodes Beyond Li Metals

The use of Li metal as anodes in ASSLIBs continues to face significant challenges, including lithium dendrite growth and the formation of undesirable SEI layers. These issues deteriorate the performance of ASSLIBs in long cycling and can even cause short circuits. The high discharge voltage of LNMO cathodes offers an opportunity to employ alternative anodes, such as TM oxides (V_2_O_5_, RuO_2_, TiO_2_, MnO_2_, etc.) and zero‐strain Li_4_Ti_5_O_12_, which enable excellent electrochemical and mechanical stability with SSEs. Developing novel metal oxide‐based anode materials with high specific capacities and low electrochemical potentials holds significant promise. These materials offer the potential to enhance the cycling stability of ASSLIBs while addressing the limitations associated with lithium metal anodes.

### 4.5 Exploiting Novel Technologies in ASSLIB Fabrication

#### 4.5.1 3D printing for Custom and Complex Designs

Versatile 3D printing techniques, with their high accuracy and efficiency, hold great promise for creating customized and complex designs that are difficult or impossible to attain through conventional fabrication methods. The successful fabrication of various composite cathodes using 3D printing demonstrated its feasibility in preparing engineering‐designed composite cathodes. Future advancements are anticipated to explore the potential of 3D printing to produce ASSLIBs with innovative and diversified structural designs, unlocking new possibilities for performance enhancement and material optimization.

#### 4.5.2 Integration of Artificial Intelligence (AI)

The integration of AI technologies, machine learning algorithms, and robotic synthesis could revolutionize the development of high‐performance ASSLIBs. These advanced tools can accelerate material discovery, optimize fabrication processes, and enhance precision in structural engineering. By leveraging AI‐driven insights, future development can achieve unprecedented advancements in the preparation and scalability of ASSLIBs with tailored properties and exceptional performance.

#### 4.5.3 Interfacial Segregation and Softening

During high‐energy mixing or mechanical milling, localized heating and shearing can induce partial decomposition or reorganization of SSEs, leading to nanoscale segregation of electrolyte phases (e.g., LiCl segregated from LPSCl). These segregated domains exhibit better conformal contact and improved ionic percolation at the particle level. Moreover, sulfide‐based SSEs like glassy Li_3_PS_4_ have relatively low glass transition temperatures (≈150–200 °C), which allow local softening or quasi‐melting. This behavior facilitates melt infiltration, acting as a quasi‐wetting mechanism that improves interface contact without compromising electrochemical stability.

#### 4.5.4 Gradient Composition or Functional Interlayers

A compositional gradient—engineered by inserting a ductile or compliant interlayer between cathode and SSE (e.g., a polymer‐sulfide composite or soft‐glassy Li^3^PS^4^)—can buffer the mechanical mismatch and reduce interfacial stress. These interlayers also help accommodate volume changes during cycling and prevent crack formation while maintaining ionic conductivity. Such gradients may also suppress interfacial reactions by limiting direct chemical contact.

#### 4.5.5 Cold Pressing and Low‐Temperature Sintering

Cold or moderate‐temperature pressing, especially when combined with pre‐synthesized dense pellet components, can enhance particle‐to‐particle adhesion without triggering unwanted interdiffusion at the interface (e.g., of Mn, Ti, or Al). In some SPS‐derived bilayer architectures, cold pressing has yielded sharp, clean interfaces with minimal void formation and low interfacial impedance.

Lastly, despite the fact that the current ASSLIBs are still associated with various challenges in achieving widespread applications in EVs, tremendous efforts are being made to seek the most promising cathode candidates that possess the advantages, including being feasible, affordable, sustainable, stable, and having long lifecycles. Co‐free and low‐Ni Mn‐based spinel cathodes have shown their strong capabilities as promising contenders, met these expectations, and demonstrated potential in ASSLIBs for future practical applications in EVs and beyond.

## Conflict of Interest

The authors declare no conflict of interest.
